# Metal- and photocatalyst-free synthesis of 3-selenylindoles and asymmetric diarylselenides promoted by visible light†

**DOI:** 10.1039/c9ra03642c

**Published:** 2019-07-23

**Authors:** Ignacio D. Lemir, Willber D. Castro-Godoy, Adrián A. Heredia, Luciana C. Schmidt, Juan E. Argüello

**Affiliations:** INFIQC-CONICET-UNC, Departamento de Química Orgánica, Facultad de Ciencias Químicas, Universidad Nacional de Córdoba, Ciudad Universitaria X5000HUA Córdoba Argentina aheredia@fcq.unc.edu.ar jea@fcq.unc.edu.ar

## Abstract

A novel and sustainable procedure was developed for the synthesis of 3-selenylindoles employing diorganyl diselenides and indoles or electron-rich arenes as starting materials. Visible blue light was used to promote the reaction without employing transition metal complexes or organic photocatalysts as sensitizers. Additives such as strong oxidants or bases were not required. Moreover, ethanol was employed as a benign solvent under mild reaction conditions. Through this easy and eco-friendly approach, several 3-selenylindoles and a number of asymmetric diarylselenides were obtained in good to excellent isolated yields.

## Abstract 2

A novel and sustainable procedure for the synthesis of 3-selenylindoles employing diorganyl diselenides and indoles or electron-rich arenes and promoted by visible light was developed.

## Graphical abstract

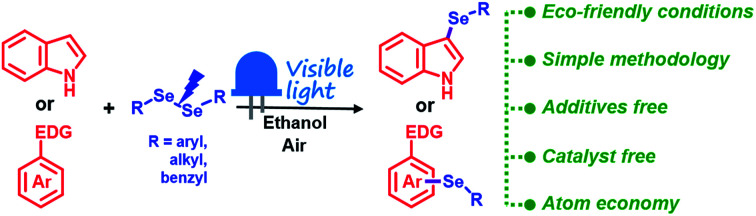

## Introduction

Pharmaceutical chemistry and materials science have been showing a growing interest in selenium-containing compounds due to their important biological activities,^1^ photo-physical properties^2^ and synthetic applications.^3^ Related to the above-mentioned issues, the development of new synthetic methodologies – which involve the selective formation of C–Se bonds – is of great importance in organic synthesis. Also, the indole moiety is found in several biomolecules which fulfil vital roles in living organisms such as the tryptophan amino acid and the serotonin neurotransmitter.^4^ Its active role in the normal development of living beings implies that many molecules which have this heterocycle in their structure could demonstrate biological activity, from enzymatic inhibitors or anti-inflammatory effects to antiviral and anticancer agents.^5^ In recent years, the synthesis of 3-chalcogenylindoles received a remarkable interest due to their palliative effects in diseases such as allergies, AIDS, obesity and cancer.^6^ In particular cases, 3-selenylindoles showed an important antitumor activity as well as an inhibitory activity of tubulin polymerization.^7^

Owing to their therapeutic potential, several synthetic methods have been reported to obtain 3-selenylindoles. Some of the less practical methodologies involve the use of 2-alkynylanilines, which are not commercially available, as starting materials being necessary their previous synthesis. Some procedures involve an organic diselenide as a selenium source in the presence of a transition metal, and an oxidant to activate both the triple and the Se–Se bonds, respectively.^8^ Other examples employ an electrophilic selenium reagent (RSeX),^9^ or even H_2_O_2_ UV-light activated to promote the ring formation step and subsequent reaction with an organic diselenide.^10^ Thus, the most convenient methodologies involve the use of commercially available indole or its derivatives as starting materials. Transition metals catalysis using organic diselenides^11^ or elemental selenium as a selenium source is also well described.^12^ The most common examples include the reaction between indoles and selenium electrophilic species,^13^ where the latter can be *in situ* generated from the corresponding diselenides using an oxidant in stoichiometric^14^ or catalytic^15^ amounts. On the other hand, procedures using bases such as K_2_CO_3_,^16^ Cs_2_CO_3_ ^17^ or *t*-BuOK^18^ assisting the formation of 3-selenylindoles are also reported.

In the last decade, there has been a surprising surge among the scientific community in the use of visible light to promote organic transformations.^19^ Visible light has relatively low energy, ensuring selectivity, and involves the possibility of the use of sunlight as an everlasting energy source. Recently, visible-light assisted photo-redox catalysis has been applied to numerous organic transformations.^20^ Many reports involve the use of transition metal complexes based on iridium (Ir) or ruthenium (Ru) due to their excellent photophysical and photochemical properties.^21^ However, their application in pharmaceutical and medicinal chemistry is limited because of economic, sustainable, toxicity and scale-up reasons. In contrast, inexpensive and less toxic organic dyes have emerged as an attractive alternative to catalysts based on rare metals.^22^ Despite the spectacular applications and advantages of applying photo-redox catalysis with transition metal complexes or dyes, in recent years organic transformations assisted by visible light in the absence of any photocatalysts have begun to attract greater attention, being the cost reduction derived from the elimination of the catalyst separation steps its main advantage.^23^ In this particular case, 3-selenylindoles have been successfully obtained using photo-redox catalysis and visible light, employing transition metal complexes^24^ and dyes.^25^ It is worth mentioning that methodologies tending to obtain 3-selenylindoles in the absence of both photocatalysts and additives have not been deeply explored yet.^23*b*,26^ In this work, visible light was used to promote the selenization reaction *via* a C_sp^2^_–H activation from indole and organic diselenides as starting materials in the absence of photocatalysts, additives and under eco-friendly reaction conditions. In parallel, during the drafting of this manuscript, Kumar *et al.* published their findings about the synthesis of chalcogenyl indoles employing visible light,^27^ even though our results are quite similar, we extended the methodology to dialkyl and dibenzyl selenides. Moreover, our experiment findings lead us to a completely different mechanistic scenario.

## Results and discussion

**Scheme 1:**
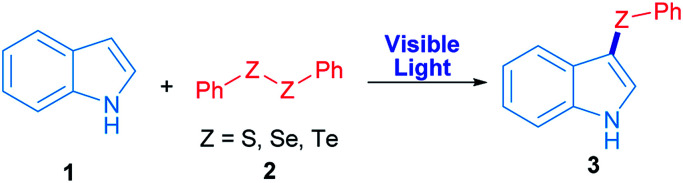
Synthesis of 3-chalcogenylindole employing visible light.

Radicals centred on S, Se and Te are frequently used in organic molecules functionalization. In the case of these chalcogens, they can be easily produced by direct irradiation of diorganyl dichalcogenides.^28^ As a proof of concept, in this work phenyl chalcogenide radical generation was proposed (Scheme 1) starting from the corresponding indole heterocycle (1) as a radical acceptor and diphenyl dichalcogenide (2) to give the corresponding 3-chalcogenylindole (3).

Diphenyl diselenide reacted with indole under 3 W blue LED photo-irradiation under aerobic conditions (see Table S1†). Considering these preliminary results, the corresponding optimization and control reactions were carried out in order to obtain the 3-phenylselenylindole (3a).

The optimization and control reactions are shown in Table 1. As starting settings, the reactions were performed for 24 h under air atmosphere, and employing a 3 W blue LED lamp as an irradiation source. In a non-polar solvent such as toluene, compound 3a was obtained in a very poor yield (Table 1, entry 1). In polar non-protic solvents, better results were observed for 3a (Table 1, entries 2–7), where ethyl acetate and acetone showed the best results, 84% and 91% yield respectively. In protic polar solvents such as PEG200, glycerol and water, 20%, 5% and 57% yields of 3a were obtained (Table 1, entries 8–10); however, 3a was quantitatively obtained when methanol and ethanol were used as solvents (Table 1, entries 11 and 12). As a result, ethanol was chosen as a less toxic and environmentally friendly solvent to continue the control experiments. When the reaction was performed in the absence of irradiation, the whole starting materials were recovered and 3a was not detected (Table 1, entry 13); therefore, visible light is essential to promote the synthesis of 3-selenylindoles. In addition, atmosphere screening was also explored: under nitrogen inert atmosphere the reaction was inhibited and 3a was only obtained in a 20% yield (Table 1, entry 14). Moreover, under oxygen saturated atmosphere a complete conversion was observed at the same reaction time (Table 1, entry 15). In order to use stoichiometric amounts of reagents and decrease the reaction time, 0.5 equiv. of 2a and 18 h irradiation was used. In this condition, 3a was obtained quantitatively under aerobic conditions (Table 1, entries 16 and 17). Finally, the reaction was not completed by lowering the reaction time to 12 h, where 3a was obtained in 61% and 94% yield when the reaction was carried out under air and oxygen atmospheres, respectively (Table 1, entries 18 and 19). These results suggest that oxygen plays a fundamental role in the reaction mechanism. However, in order to broaden this methodology, we have decided to employ 18 h under air atmosphere as optimized reaction conditions.

**Table 1:** Optimization and control reactions^a^

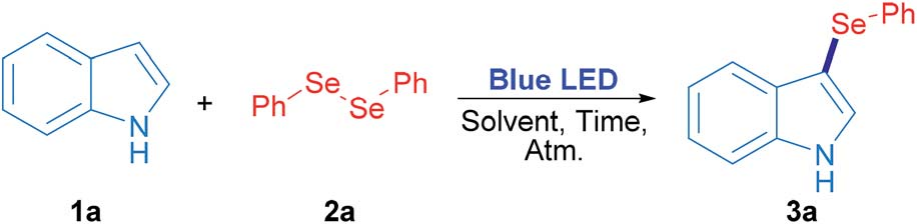				
Entry	Solvent	Atm.	Time (h)	Yield^b^ (%)
1	Toluene	Air	24	9
2	THF	Air	24	54
3	DMSO	Air	24	23
4	DMF	Air	24	52
5	EtOAc	Air	24	84
6	DCM	Air	24	56
7	Acetone	Air	24	91
8	PEG200	Air	24	20
9	Glycerol	Air	24	5
10	Water	Air	24	57
11	Methanol	Air	24	100
12	Ethanol	Air	24	100
13^c^	Ethanol	Air	24	0
14	Ethanol	Nitrogen	24	20
15	Ethanol	Oxygen	24	100
**16** d	**Ethanol**	**Air**	**18**	**100 (89)** e
17^d^	Ethanol	Oxygen	18	100
18^d^	Ethanol	Air	12	61
19^d^	Ethanol	Oxygen	12	94

a Reaction conditions: 1a (0.15 mmol), 2a (0.75 equiv.), solvent (2 mL) at room temperature irradiated with 3 W blue LED (467 nm).

b Yields obtained by GC using internal standard method.

c Reaction performed under dark.

d 0.5 equiv. of 2a were used.

e Isolated yield.

**Scheme 2:**
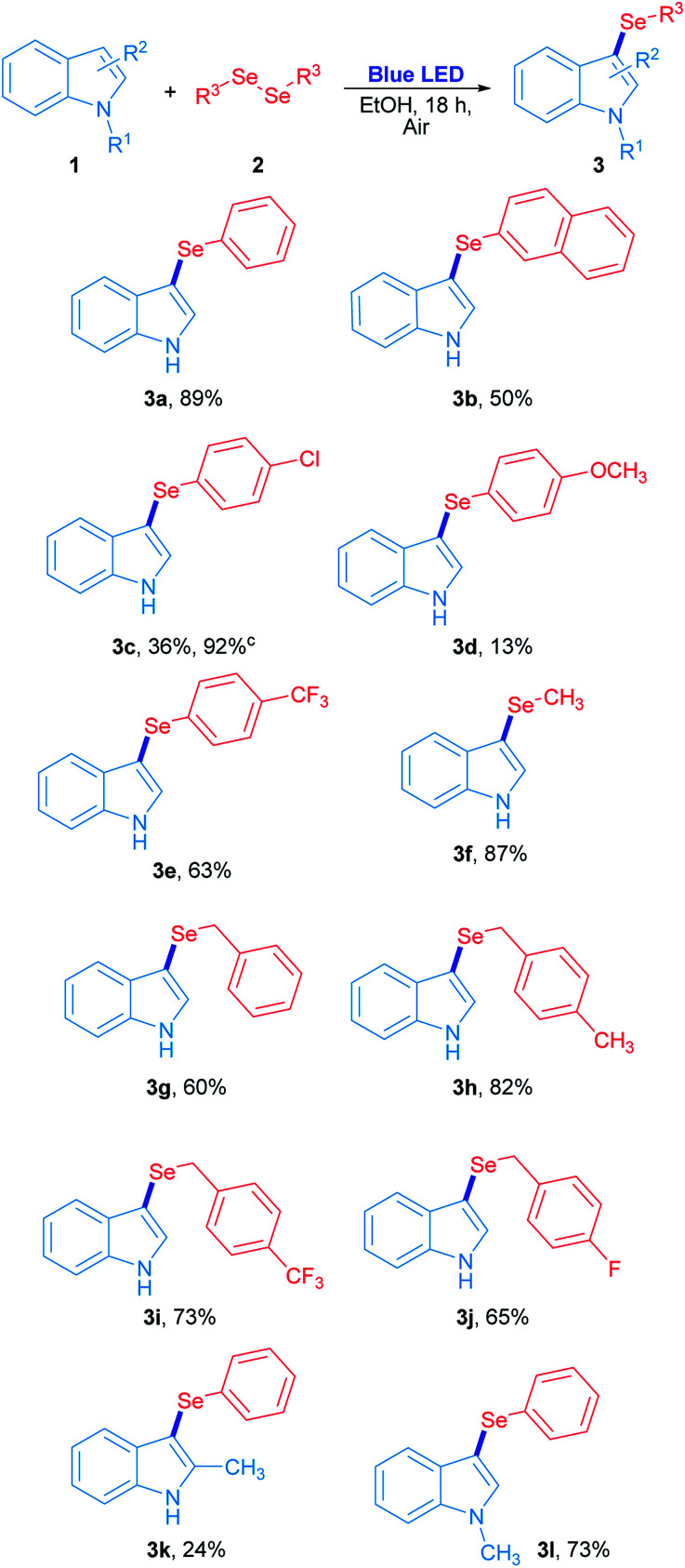
Synthesis of 3-selenylindoles using visible light.^*a*,*b a*^Reaction conditions: 1 (0.15 mmol) and 2 (0.5 equiv.) in ethanol (2 mL) irradiated for 18 h with 3 W blue LED (467 nm) at room temperature under air atmosphere. ^*b*^Isolated yields. ^*c*^Reaction irradiated for 36 h.

The scope study for the synthesis of 3-selenylindoles using visible light was carried out using many substituted indoles and diaryl, dibenzyl and dialkyl diselenides under optimized reaction conditions (Scheme 2). The 3-selenylindole 3a was obtained in 89% isolated yield when 2a was employed. On the other hand, a change in the electronic character of the aromatic ring attached to selenium atom affected the yields of the 3-selenylindoles obtained. When bis(2-naphthyl)diselenide 2b was used, the corresponding 3-selenylindole 3b was achieved in 50% yield. The drop in the yield of 3b can be explained by an increase in the stability of the 2-selenylnaphthyl radical compared to the selenylphenyl radical, lowering its reactivity. When bis(4-chlorophenyl)diselenide was used, the corresponding 3-selenylindole 3c was found in 36% yield. Nevertheless, the performance was considerably improved when the reaction time was extended to 36 h, obtaining 3c in 92% isolated yield. Compound 3d was obtained in only 13% when bis(4-methoxyphenyl)diselenide was employed. Unfortunately, this result could not be improved even under an oxygen-saturated atmosphere or increased reaction time. Conversely, when a good electron-withdrawing group attached to the diaryl selenide reagent, such as trifluoromethyl, was used, the corresponding selenylindole 3e was obtained in 63% isolated yield. Alkyl and benzyl diselenides were also effective: the 3-methylselenylindole 3f was isolated in 87% yield when dimethyl diselenide was used. In addition, benzylic diselenides also showed a very good reactivity in this system. When dibenzyl diselenide was used, 3g was obtained in 60% yield. No significative effects in reaction performance were observed by changing the substituents from *p*-methyl (2h), *p*-trifluoromethyl (2i) to *p*-fluorine (2j) groups in the dibenzyl diselenide substrates, which resulting in 82% (3h), 73% (3i) and 65% (3j) isolated yield of the corresponding 3-selenylindoles. Besides, this study was extended to several substituted indoles where only 2-methylindole and *N*-methylindole gave their corresponding 3-phenylselenylindole 3k and 3l in 24% and 73% yield, respectively, in the presence of diphenyl diselenide. On the contrary, there is no reaction for indoles bearing electron-withdrawing groups.^29^ All these results suggest that the indole heterocycle would be involved in an electrophilic substitution reaction with an electron-poor selenium species, which is produced by visible light irradiation.

**Scheme 3:**
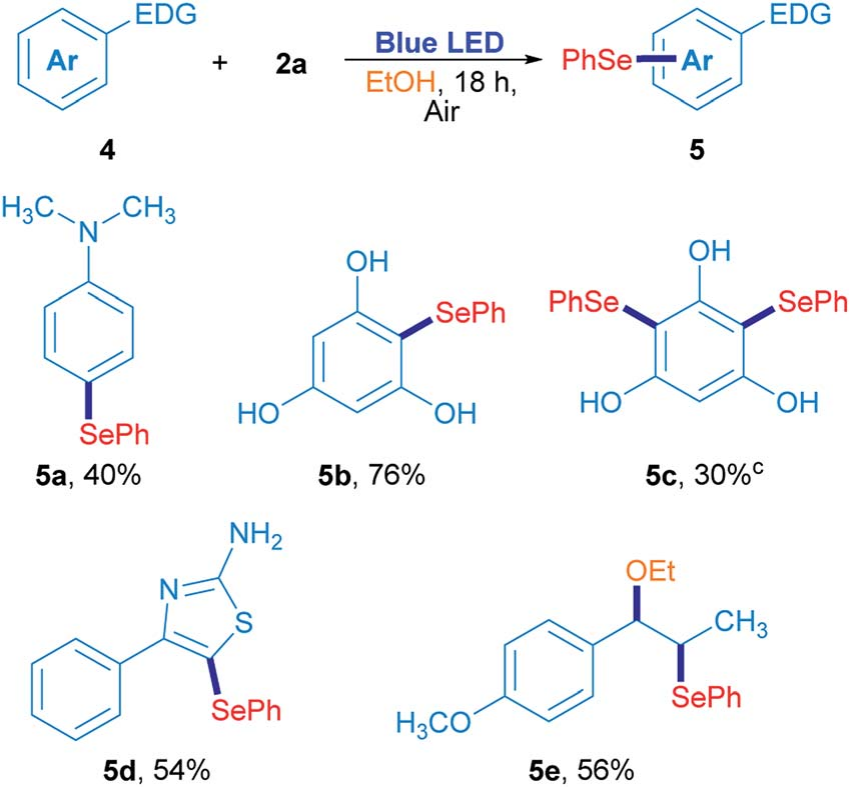
Photoinduced arylselenation of electron-rich arenes.^*a*,*b a*^Reaction conditions: 4 (0.15 mmol) and 2a (0.5 equiv.) in ethanol (2 mL) irradiated for 18 h with 3 W blue LED (467 nm) at room temperature under air atmosphere. ^*b*^Isolated yields. ^*c*^Reaction performed with 1 equiv. of 2a, irradiated for 36 h.

In order to confirm this hypothesis, some reactions were carried out employing electron-rich arenes, using diphenyl diselenide as a selenium source under the optimized reaction conditions; results are shown in Scheme 3. *N*,*N*-Dimethyl-4-(phenylselanyl)aniline 5a was obtained in 40% isolated yield when 2a reacted with *N*,*N*-dimethylaniline. Similarly, phloroglucinol was also reactive with 2a, giving the corresponding asymmetric selenide 5b in 76% yield. For further substitution, phloroglucinol was placed to react with an excess of 2a (2a/phloroglucinol ratio of 2) and the mixture was irradiated for 36 h. Under these conditions, 5c was obtained in 30% yield. The heterocycle 4-phenylthiazole-2-amine was also used as a substrate and the corresponding 5-selenylthiazole 5d was obtained in 54% isolated yield (Scheme 3). Finally, *trans*-anethole was used as an electron-rich arene. Surprisingly, substitution at the alkenyl C=C double bond was preferred to the anisyl moiety and the compound 5e was obtained in 56% isolated yield. In this particular case, the phenylselenyl moiety was attached at C-β from *trans*-anethole, while an ethoxy group from the solvent was located at the C-α.^30^ These results suggest that a sort of electrophilic substitution mechanism, which involves an electrophilic selenium intermediate that reacts selectively with electron-rich arenes namely, indoles, thiazoles, anilines and phenols.

**Fig. 1:**
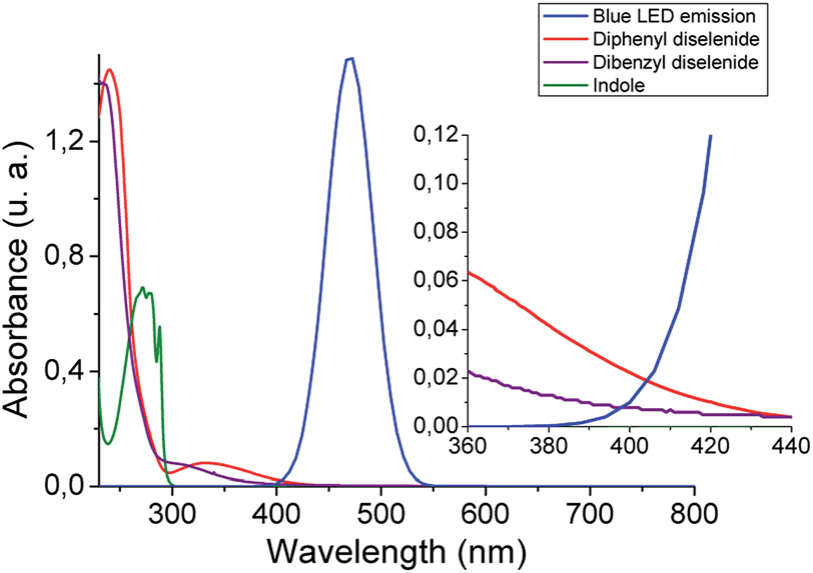
Absorption spectra of diselenides (0.06 mM) and indole (0.06 mM) in ethanol, and blue LED emission. Inset: zoom from 360 to 440 nm spectrum zone.

With the aim of getting a deeper insight into the reaction mechanism spectroscopic, switch on–off, non-kinetic, and transient absorption experiments were carried out. The absorption spectra of organic diselenides were obtained to confirm their blue absorption from the LED source (Fig. 1). The diphenyl diselenide absorption spectrum (0.06 mM) was taken as an arylic example, and dibenzyl diselenide (0.06 mM) as a benzylic/alkylic one, using ethanol as a solvent. Also, the spectral emission from blue LED used for the reactions was overlapped in the Fig. 1. In the spectroscopy study, the reagents concentration was considerably lower than in our reaction conditions (see inset Fig. 1). Nevertheless, the absorption of diselenides overlaps the blue LED emission, allowing the homolytic Se–Se bond breaking. Furthermore, the absorption spectrum of the indole solution in ethanol (0.06 mM) is also measured, showing no absorbance at wavelengths above 300 nm. Thus, blue LED emission does not overlap the indole absorption spectrum, ensuring that no excited species coming from the heterocycle are formed. No photo-products through direct indole irradiation can be formed, which could probably occur when a shorter wavelengths irradiation source is used.

**Fig. 2:**
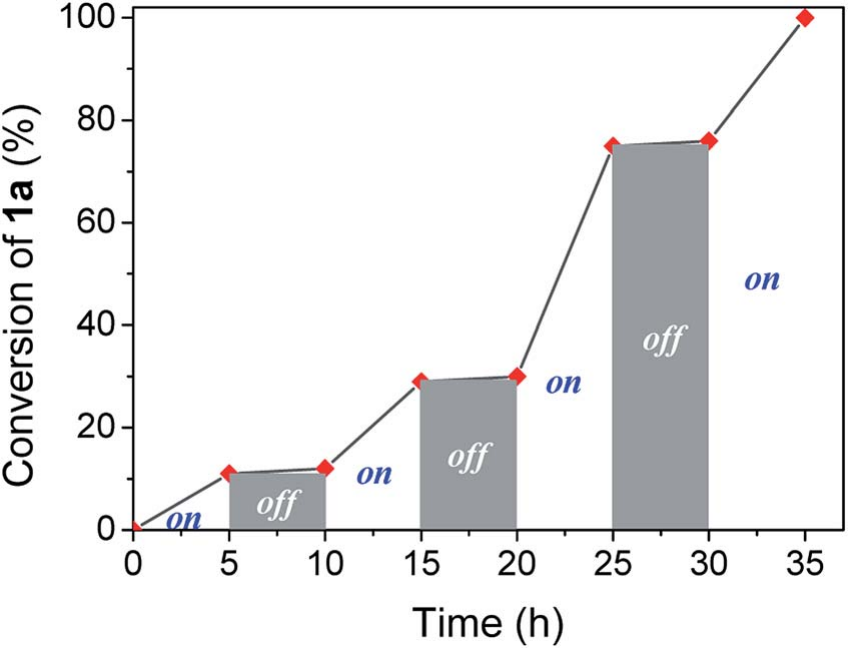
Profile of switching on–off experiment by following indole conversion along light on–off periods of time.

Visible light irradiation is essential to carry out the reaction. However, photons can be involved in an initiation step, starting a chain reaction, or a permanent assistance, where the photons are needed to excite molecules during all the reaction time. In order to study the irradiation effect, a switched on–off experiment was performed (Fig. 2). Indole conversion was only observed when the system was exposed to photo-irradiation, while the reaction was transitionally stopped during the dark spans, disregarding a light initiated chain mechanism.

**Scheme 4:**
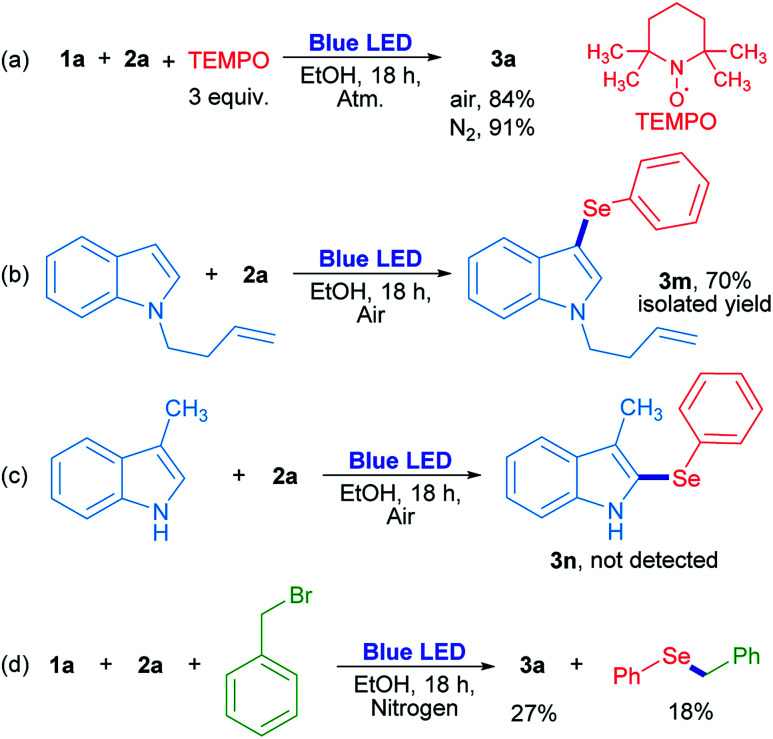
Control reactions.

In order to evaluate the chemical nature of the intermediates, non-kinetic experiments were done (Scheme 4). The compound 3a was obtained in 84% and 91% yield when the reaction was carried out in the presence of 3 equiv. of 2,2,6,6-tetramethyl-1-piperidinyloxy (TEMPO) under air and nitrogen atmosphere, respectively (Scheme 4a). This result suggests that TEMPO acts as an oxidant rather than as a radical trapping reagent. In this case, TEMPO plays the role of oxygen when the latter is absent. On the other hand, *N*-3-butenylindole was used as a “clock” substrate, where a hypothetical radical intermediate, produced by a radical addition reaction, progressed towards a cyclization product formation.^31^ However, the 3-selenylindole derivative 3m was obtained in 70% isolated yield where the alkenyl moiety remained unchanged (Scheme 4b). This result indicates that the selenium reactive intermediate shows selectivity towards indole heterocycle against the nearby C=C moiety. The absence of a cyclized product indicates that the rearomatization process is faster than a plausible cyclization reaction. For indole compounds, C-3 is the most reactive position in an electrophilic substitution reaction.^32^ When position 3 was blocked with a methyl group, neither the corresponding 2-selenylindole, nor any other isomer were detected (Scheme 4c). At this stage, these results suggest that a purely radical pathway way would not be operating in these reactions.

The role of oxygen and the trapping of any reactive intermediate were also investigated. The presence of hydrogen peroxide as the ultimate reaction product from oxygen was proved by a positive test with I_2_/starch indicator from the aqueous layer by the end of the reaction. Moreover, the reaction evolved to a 20% yield in the absence of oxygen (Table 1, entry 14), but when benzyl bromide was added to the reaction, 3a was obtained in a similar 27% yield together with benzyl phenyl selenide (18% determined by GC-MS). The latter can be formed by a nucleophilic substitution reaction between benzeneselenol as a reaction intermediate and benzyl bromide (Scheme 4d).

**Scheme 5:**
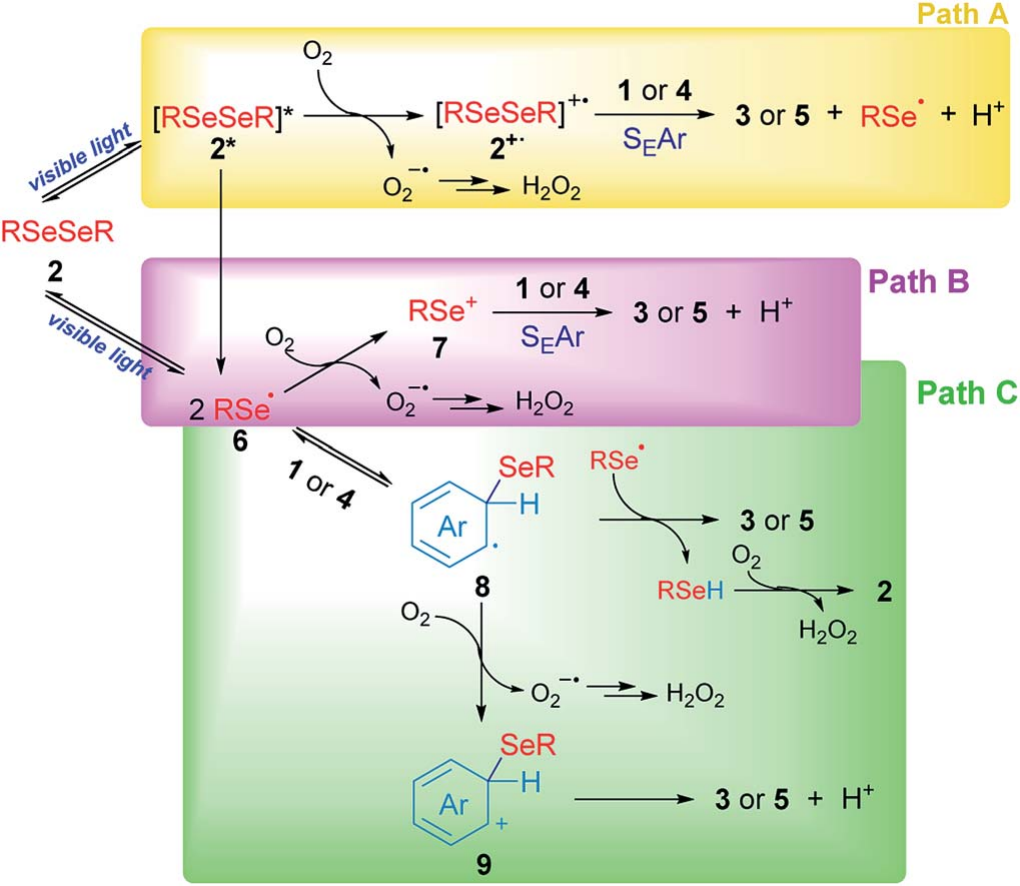
Proposed mechanisms for the synthesis of selenium compounds.

Based on our experimental results, three different pathways – shown in Scheme 5 – can be proposed as reaction mechanisms for the 3-substituted selenylindoles (3a–m) and asymmetric diaryl selenides (5a–d) formation.

In Scheme 5, path A, visible light excites diselenide 2 to form its electronically excited species 2*, which can lead to a Se–Se homolytic cleavage to give selenyl radicals 6, 2* may react with oxygen to produce the electrophilic species 2^+^˙ and superoxide anion (O_2_^−^˙). The intermediate 2^+^˙ would be able to react with aromatic rings such as indole 1 or arenes 4 through S_E_Ar to obtain the aryl selenides observed in 3 and 5, together with selenyl radicals, which dimerize to produce 2 and protons (H^+^) and the superoxide anion would account for the hydrogen peroxide formation. However, this mechanism can be ruled out for two reasons: first the low-lying excited state of diselenides is dissociative which indicates that a reaction between excited 2, if exists, and oxygen, is very unlikely. This was further confirmed by transient absorption spectroscopy studies (see Fig. S2 and S3†). Secondly, this pathway is totally dependent on oxygen concentration and some reaction is observed even in the very absence of oxygen (Table 1, entry 14).

Another possible operating mechanism is shown in Scheme 5, path B. Here, photo-homolytically formed selenyl radicals 6 was oxidized by an oxygen molecule, where superoxide anion (O_2_^−^˙) and electrophilic selenium species 7 are formed. Arylselenyl cation then is able to react by S_E_Ar with indole or electron-rich arenes to account for the observed products. Even, the electronic deficiency of the intermediaries 2^+^˙, 6 and 7 could be mitigated through their interaction with a diselenide molecule in its neutral form.^33^ However, it has been well established that, unlike carbon centred radicals, selenium centred radical are unreactive towards oxygen, which indicates that this pathway is also unlikely.^34^ This was further confirmed by transient absorption spectroscopy where only a phenylselenyl radical was observed upon laser excitation from a sample of Ph_2_Se_2_, regardless of the source of excitation (266 or 355 nm) or the solvent used (CH_3_CN or ethanol). Furthermore, neither the top optical density (maximum absorption at the laser excitation pulse), nor the PhSe˙ lifetime monitored at 490 nm were affected by the presence of oxygen. For further details see Fig. S1–S3 and Table S2 in the ESI.† Besides, an indole spectroscopic study was also carried out: its fluorescence quenching experiment with diselenide as quencher proved to be static, since their absorption spectra significantly overlap (see Fig. S4–S6 and Table S3†). Even though the formation of excited indole is unlikely under our experimental conditions, its triplet excited state is considered a well-known sensitizer for singlet oxygen formation^35^ rather than electron transfer donor.^27^

Finally, another possible scenario is shown in Scheme 5, path C. In this case, the photolytically formed selenyl radical 6 reacts with the aromatic rings to generate the radical intermediates 8. To restore the aromaticity, an intermolecular hydrogen atom abstraction can be proposed between the remaining phenylselenyl radical and 8, giving the corresponding asymmetric diaryl selenide and benzeneselenol, the latter being oxidized in the presence of molecular oxygen to give H_2_O_2_ and regenerate 2. The mediation of benzeneselenol was determined by the experiment under an inert atmosphere and in the presence of benzyl bromide (Scheme 4d). In this case, a strong inhibition of the reaction was again observed, and benzyl phenyl selenide was found as proof of PhSeH presence. Undoubtedly, this condition is not efficient in order to obtain 3 and 5, but is the only one that was able to operate under anaerobic conditions. However, the formation of a cationic species 9 was also proposed, generated by oxidation of intermediate 8 rendering the well stablished Wheland's intermediate, which rapidly aromatizes by a proton lost. This is more likely the case of anethol where the benzyl cation intermediate is trapped by the solvent giving 5e as a final product (Scheme 3).

Summing up, according to our experimental findings, the more likely reaction mechanism resulted in path C from Scheme 5, where Ar_2_Se_2_ is the only reagent able to absorb visible light giving arylselenyl radicals which selectively react with electron-rich aromatic compounds. Also, oxygen is essential to renew Ar_2_Se_2_ at the expense of ArSeH formed in the reaction media.

## Conclusions

Summarizing, we have developed a novel synthetic methodology for the regioselective obtention of several 3-selenylindoles from indole and organic diselenides promoted by visible light in good to excellent isolated yields. This could be a greener synthetic strategy than those currently reported, since atomic economy is fulfilled and transition metal complexes photocatalysts and organic dyes, bases or strong oxidants are not required. The reaction is carried out under mild reaction conditions, at room temperature and oxygen from air is used as a green oxidizing agent. Furthermore, taking advantage of the electrophilic properties of the involved intermediates, some asymmetric diaryl selenides were synthesized employing electron-rich arenes under optimized conditions.

## Experimental section

### Materials and methods

Indoles, *N*,*N*-dimethylaniline, phloroglucinol, *trans*-anethol, diphenyl diselenide and dimethyl diselenide were all high-purity commercial samples used without further purification. PEG200, DMSO, acetonitrile, glycerol, toluene and ethanol were used without further purification. THF, ethyl acetate, acetone and dichloromethane were distilled and stored over activated 4 Å molecular sieves. Dinaphthyl, bis(4-chlorophenyl) and bis(4-methoxyphenyl)diselenides were synthetized according to reported procedures from their corresponding Grignard reagents and selenium powder. Dibenzyl diselenides were synthetized by a nucleophilic substitution reaction between benzyl halides and diselenide anion (Se_2_^2−^) obtained with selenium powder, sodium borohydride and ethanol in DMF. *N*-3-butenylindole was obtained by nucleophilic substitution reaction between indole and 3-butenyl bromide with K_2_CO_3_ as a base in DMF. The heterocycle 4-phenylthiazole-2-amine was synthetized from 2-bromoacetophenone and thiourea in ethanol. The reaction products were isolated by flash column chromatography (silica gel, eluting with 8 : 2 hexanes/ethyl acetate) from the reaction mixture and characterized by ^1^H, ^13^C and ^77^Se NMR and mass spectrometry. ^1^H, ^13^C, ^19^F and ^77^Se NMR spectra were recorded at 400, 101, 377 and 76 MHz respectively on a Bruker 400 spectrometer with CDCl_3_ as a solvent. All spectra were reported in *δ* (ppm) relative to residual solvent signal (*δ*_H_ (CHCl_3_) = 7.26 ppm). The chemical shifts in ^77^Se spectra were given in ppm using diphenyl diselenide (PhSeSePh) diluted in CDCl_3_ as an external standard (*δ*_Se_ (CDCl_3_) = 463 ppm). Gas chromatographic analyses were performed on Agilent 5890 with a flame-ionization detector, on a 30 m capillary column of 0.32 mm × 0.25 μm film thickness, with a 5% phenylpolysiloxane phase. GC-MS analyses were conducted on Agilent 7890 employing a 30 m × 0.25 mm × 0.25 μm with a 5% phenylpolysiloxane phase column. Ionization was achieved by electronic impact (70 eV) and detection set up positive mode. HRMS spectra were recorded on a GCT Premie orthogonal acceleration time-of-flight (oa-TOF) mass spectrometer. Ionization was achieved by electrospray and detection set on positive mode.

### Spectroscopic measurements

Laser flash photolysis experiments were performed using the frequency-tripled output (355 nm) of a Surelite II-10 Nd:YAG laser as an excitation source. Transient species were monitored at a right angle to the laser beam (5 ns duration pulses) using an LP900 Flash Photolysis System (Edinburgh Analytical Instruments). Data were collected using a commercial oscilloscope. Signal averaging was routinely performed to increase the signal-to-noise ratio. Samples, contained in 1 cm path length quartz cells, were bubbled with the corresponding atmosphere prior to irradiation.

### Synthetic procedures

#### Experimental procedure for the synthesis of 3-selenylindoles and diaryl selenides

The reactions were carried out in a 10 mL glass vial, equipped with a rubber septum and a magnetic stirrer. Indoles (1, 0.15 mmol) or electron-rich arenes (4, 0.15 mmol), diorganyl diselenide (2, 0.075 mmol) were dissolved in ethanol (2 mL), and the mixture was irradiated with 3 W blue-LED (467 nm) and stirred under air atmosphere for 18 h. Then, reaction crude was placed into a separatory funnel and ethyl acetate (15 mL) and water (15 mL) were added. The mixture was then stirred. The organic layer was separated, and the aqueous layer was extracted with ethyl acetate (2 × 15 mL). The combined organic extract was dried over anhydrous Na_2_SO_4_. Finally the product was isolated by column chromatography (silica gel, eluent: 8 : 2 hexanes/ethyl acetate). The identity of all the products was confirmed by ^1^H, ^13^C, ^19^F and ^77^Se NMR.

### Spectroscopic characterization of all synthetized compounds

#### 3-(Phenylselanyl)-1*H*-indole (3a)^25^

Following the general procedure for the reaction in one step, indole (17.6 mg, 0.15 mmol) and diphenyl diselenide (31.4 mg, 0.075 mmol) were placed in a glass vial with 2 mL of ethanol. Then, the mixture was irradiated with 3 W blue-LED (467 nm), stirring for 18 h. Purification was performed by flash column chromatography affording 3a as a white solid (36.3 mg, 89% yield). ^1^H NMR (400 MHz, CDCl_3_) *δ* 8.41 (s, 1H), 7.65 (d, *J* = 7.8 Hz, 1H), 7.52–7.39 (m, 2H), 7.35–7.02 (m, 7H). ^13^C NMR (101 MHz, CDCl_3_) *δ* 136.56, 133.94, 131.32, 130.15, 129.09, 128.86, 125.75, 123.10, 121.02, 120.55, 111.48, 98.33. ^77^Se (76 MHz, CDCl_3_) *δ* 210.8.

#### 3-(Naphthalen-2-ylselanyl)-1*H*-indole (3b)^25^

Following the general procedure for the reaction in one step, indole (17.6 mg, 0.15 mmol) and dinaftyl diselenide (30.9 mg, 0.075 mmol) were placed in a glass vial with 2 mL of ethanol. Then, the mixture was irradiated with 3 W blue-LED (467 nm), stirring for 18 h. Purification was performed by flash column chromatography affording 3b as a brown oil (24.2 mg, 50% yield). ^1^H NMR (400 MHz, CDCl_3_) *δ* 8.45 (s, 1H), 7.76–7.68 (m, 2H), 7.66 (d, *J* = 7.8 Hz, 1H), 7.60 (dd, *J* = 8.7, 5.1 Hz, 2H), 7.54 (d, *J* = 2.6 Hz, 1H), 7.46 (d, *J* = 8.3 Hz, 1H), 7.40–7.33 (m, 3H), 7.31–7.23 (m, 1H), 7.17 (t, *J* = 7.4 Hz, 1H). ^13^C NMR (101 MHz, CDCl_3_) *δ* 136.62, 134.16, 131.92, 131.47, 131.34, 130.18, 128.42, 127.87, 127.19, 127.13, 127.06, 126.40, 125.44, 123.15, 121.08, 120.58, 111.51, 98.48. ^77^Se NMR (76 MHz, CDCl_3_) *δ* 214.7.

#### 3-((4-Chlorophenyl)selanyl)-1*H*-indole (3c)^25^

Following the general procedure for the reaction in one step, indole (17.6 mg, 0.15 mmol) and 1,2-bis(4-chlorophenyl)diselenide (28.6 mg, 0.075 mmol) were placed in a glass vial with 2 mL of ethanol. Next, the mixture was irradiated with 3 W blue-LED (467 nm), stirring for 36 h. Purification was performed by flash column chromatography affording 3c as a brown oil (42.3 mg, 92% yield). ^1^H NMR (400 MHz, CDCl_3_) *δ* 8.64 (s, 1H), 7.72 (d, *J* = 8.3 Hz, 1H), 7.61 (d, *J* = 7.8 Hz, 1H), 7.48–7.40 (m, 2H), 7.31–7.25 (m, 2H), 7.18 (dd, *J* = 17.6, 8.0 Hz, 3H), 7.09 (d, *J* = 8.5 Hz, 2H). ^13^C NMR (101 MHz, CDCl_3_) *δ* 136.59, 132.22, 131.71, 131.43, 130.10, 129.84, 129.13, 128.30, 123.86, 123.17, 121.10, 120.30, 112.64, 111.62. ^77^Se NMR (76 MHz, CDCl_3_) *δ* 213.47.

#### 3-((4-Methoxyphenyl)selanyl)-1*H*-indole (3d)^25^

Following the general procedure for the reaction in one step, indole (17.6 mg, 0.15 mmol) and 1,2-bis(4-methoxyphenyl)diselenide (27.91 mg, 0.075 mmol) were placed in a glass vial with 2 mL of ethanol. Next, the mixture was irradiated with 3 W blue-LED (467 nm), stirring for 18 h. Purification was performed by flash column chromatography affording 3d as a yellow solid (5.9 mg, 13% yield). ^1^H NMR (400 MHz, CDCl_3_) *δ* 8.33 (s, 3H), 7.65 (d, *J* = 7.8 Hz, 3H), 7.47–7.35 (m, 6H), 7.23 (d, *J* = 10.1 Hz, 7H), 7.16 (t, *J* = 7.4 Hz, 3H), 6.70 (d, *J* = 8.7 Hz, 6H), 3.71 (s, 10H). ^13^C NMR (101 MHz, CDCl_3_) *δ* 158.50, 136.49, 131.42, 130.71, 130.03, 123.53, 122.97, 120.88, 120.49, 114.91, 111.43, 99.4, 55.40. ^77^Se NMR (76 MHz, CDCl_3_) *δ* 201.50.

#### 3-((4-(Trifluoromethyl)phenyl)selanyl)-1*H*-indole (3e)^25^

Following the general procedure for the reaction in one step, indole (17.6 mg, 0.15 mmol) and 1,2-bis(4-trifluoromethylphenyl)diselenide (33.6 mg, 0.075 mmol) were placed in a glass vial with 2 mL of ethanol. Next, the mixture was irradiated with 3 W blue-LED (467 nm), stirring for 18 h. Purification was performed by flash column chromatography affording 3e as a colorless oil (32.1 mg, 63% yield). ^1^H NMR (400 MHz, CDCl_3_) *δ* 8.53 (s, 1H), 7.58 (d, *J* = 8.2 Hz, 1H), 7.53 (d, *J* = 2.6 Hz, 1H), 7.48 (d, *J* = 8.2 Hz, 1H), 7.35 (d, *J* = 8.2 Hz, 2H), 7.32–7.27 (m, 3H), 7.20 (t, *J* = 8.2 Hz, 1H). ^13^C NMR (101 MHz, CDCl_3_) *δ* 136.77, 133.79, 129.14, 128.14, 127.95 (q, *J*_C–F_ = 287.8 Hz), 127.26 (q, *J*_C–F_ = 34.8 Hz), 125.83, 123.45 (q, *J*_C–F_ = 3.4 Hz), 121.22, 119.24, 118.24, 110.05, 96.15. ^77^Se NMR (76 MHz, CDCl_3_) *δ* 224.40. ^19^F NMR (377 MHz, CDCl_3_) *δ* −62.44.

#### 3-(Methylselanyl)-1*H*-indole (3f)^36^

Following the general procedure for the reaction in one step, indole (17.6 mg, 0.15 mmol) and 1,2-dimethyldiselenide (7.12 μL, 0.075 mmol) were placed in a glass vial with 2 mL of ethanol. Next, the mixture was irradiated with 3 W blue-LED (467 nm), stirring for 18 h. Purification was performed by flash column chromatography affording 3f as a pale brown oil (27.4 mg, 87% yield). ^1^H NMR (400 MHz, CDCl_3_) *δ* 8.22 (s, 1H), 7.76 (d, *J* = 7.3 Hz, 1H), 7.33 (d, *J* = 2.4 Hz, 1H), 7.29–7.19 (m, 2H), 2.24–2.19 (m, 3H). ^13^C NMR (101 MHz, CDCl_3_) *δ* 136.44, 129.92, 129.19, 122.80, 120.54, 120.23, 111.44, 100.44, 9.36. ^77^Se NMR (76 MHz, CDCl_3_) *δ* 10.56.

#### 3-(Benzylselanyl)-1*H*-indole (3g)^25^

Following the general procedure for the reaction in one step, indole (17.6 mg, 0.15 mmol) and 1,2-dibenzyldiselenide (25.51 mg, 0.075 mmol) were placed in a glass vial with 2 mL of ethanol. Next, the mixture was irradiated with 3 W blue-LED (467 nm), stirring for 18 h. Purification was performed by flash column chromatography affording 3g as a brown oil (25.7 mg, 60% yield). ^1^H NMR (400 MHz, CDCl_3_) *δ* 8.21 (s, 1H), 7.68 (d, *J* = 7.7 Hz, 1H), 7.39 (d, *J* = 7.9 Hz, 1H), 7.29–7.14 (m, 4H), 7.05 (d, *J* = 2.5 Hz, 2H), 3.88 (s, 2H). ^13^C NMR (101 MHz, CDCl_3_) *δ* 139.93, 136.33, 134.95, 130.75, 128.93, 128.31, 126.59, 122.74, 122.04, 120.63, 120.31, 118.71, 111.35, 111.03, 77.16, 32.31. ^77^Se NMR (76 MHz, CDCl_3_) *δ* 191.50.

#### 3-((4-Methylbenzyl)selanyl)-1*H*-indole (3h)^36^

Following the general procedure for the reaction in one step, indole (17.6 mg, 0.15 mmol) and 1,2-bis(4-methylbenzyl)diselane (27.6 mg, 0.075 mmol) were placed in a glass vial with 2 mL of ethanol. Next, the mixture was irradiated with 3 W blue-LED (467 nm), stirring for 18 h. Purification was performed by flash column chromatography affording 3h as a pale yellow solid (36.9 mg, 82% yield). ^1^H NMR (400 MHz, CDCl_3_) *δ* 8.21 (s, 2H), 7.71 (d, *J* = 7.6 Hz, 2H), 7.47–7.33 (m, 4H), 7.25–7.15 (m, 5H), 7.11–7.06 (m, 3H), 6.99 (dd, *J* = 19.5, 7.8 Hz, 10H), 3.87 (s, 5H), 2.31 (s, 8H). ^13^C NMR (101 MHz, CDCl_3_) *δ* 136.80, 136.35, 136.21, 130.66, 130.32, 129.03, 128.79, 122.70, 122.01, 120.57, 120.33, 111.34, 99.38, 77.16, 32.15, 21.22. ^77^Se NMR (76 MHz, CDCl_3_) *δ* 188.32.

#### 3-((4-(Trifluoromethyl)benzyl)selanyl)-1*H*-indole (3i)^25^

Following the general procedure for the reaction in one step, indole (17.6 mg, 0.15 mmol) and 1,2-bis(4-(trifluoromethyl)benzyl)diselenide (28.21 mg, 0.075 mmol) were placed in a glass vial with 2 mL of ethanol. Then, the mixture was irradiated with 3 W blue-LED (467 nm), stirring for 18 h. Purification was performed by flash column chromatography affording 3i as a white solid (38.9 mg, 73% yield). ^1^H NMR (400 MHz, CDCl_3_) *δ* 8.25 (s, 1H), 7.59 (d, *J* = 7.8 Hz, 1H), 7.46–7.31 (m, 3H), 7.23 (ddd, *J* = 28.4, 9.3, 5.6 Hz, 2H), 7.04 (dd, *J* = 10.9, 5.1 Hz, 3H), 3.86 (s, 2H). ^13^C NMR (101 MHz, CDCl_3_) *δ* 144.28, 136.37, 131.04, 130.99, 130.92 (q, *J*_C–F_ = 24.6 Hz), 130.22, 129.11, 127.2 (q, *J*_C–F_ = 281.6 Hz), 125.18 (q, *J*_C–F_ = 3.7 Hz), 122.20, 120.82, 120.12, 111.45, 31.36. ^77^Se NMR (76 MHz, CDCl_3_) *δ* 206.44. ^19^F NMR (377 MHz, CDCl_3_) *δ* −62.36.

#### 3-((4-Fluorobenzyl)selanyl)-1*H*-indole (3j)^25^

Following the general procedure for the reaction in one step, indole (17.6 mg, 0.15 mmol) and 1,2-bis(4-fluorobenzyl)diselenide (35.71 mg, 0.075 mmol) were placed in a glass vial with 2 mL of ethanol. Next, the mixture was irradiated with 3 W blue-LED (467 nm), stirring for 18 h. Purification was performed by flash column chromatography affording 3j as a brown oil (29.6 mg, 65% yield). ^1^H NMR (400 MHz, CDCl_3_) *δ* 8.23 (s, 1H), 7.65 (d, *J* = 7.8 Hz, 1H), 7.40 (d, *J* = 7.8 Hz, 1H), 7.26 (dd, *J* = 8.2, 6.4 Hz, 1H), 7.20 (t, *J* = 7.3 Hz, 1H), 7.03 (d, *J* = 2.6 Hz, 1H), 6.95 (dt, *J* = 8.7, 4.5 Hz, 2H), 6.85 (t, *J* = 3.1 Hz, 2H), 3.84 (s, 2H). ^13^C NMR (101 MHz, CDCl_3_) *δ* 161.75 (d, *J*_C–F_ = 240.9 Hz), 136.34, 135.74 (d, *J*_C–F_ = 3.1 Hz), 130.85, 130.40 (d, *J*_C–F_ = 8.0 Hz), 130.29, 130.22, 122.82, 120.70, 120.21, 115.05 (d, *J*_C–F_ = 21.2 Hz), 111.40, 98.90, 31.25. ^77^Se NMR (76 MHz, CDCl_3_) *δ* 197.29. ^19^F NMR (377 MHz, CDCl_3_) *δ* −116.36.

#### 2-Methyl-3-(phenylselanyl)-1*H*-indole (3k)^25^

Following the general procedure for the reaction in one step, 2-methyl indole (17.67 mg, 0.15 mmol) and diphenyl diselenide (31.4 mg, 0.075 mmol) were placed in a glass vial with 2 mL of ethanol. Next, the mixture was irradiated with 3 W blue-LED (467 nm), stirring for 18 h. Purification was performed by flash column chromatography affording 3k as a yellow oil (10.3 mg, 24% yield). ^1^H NMR (400 MHz, CDCl_3_) *δ* 8.27 (s, 1H), 7.56 (d, *J* = 7.8 Hz, 1H), 7.34 (d, *J* = 7.8 Hz, 1H), 7.24–7.00 (m, 7H), 2.56 (s, 3H). ^13^C NMR (101 MHz, CDCl_3_) *δ* 140.91, 135.97, 134.08, 131.46, 129.07, 128.56, 125.51, 122.29, 120.81, 119.99, 110.58, 13.35. ^77^Se NMR (76 MHz, CDCl_3_) *δ* 185.29.

#### 1-Methyl-3-(phenylselanyl)-1*H*-indole (3l)^25^

Following the general procedure for the reaction in one step, 1-methyl indole (18.74 μL, 0.15 mmol) and diphenyl diselenide (31.4 mg, 0.075 mmol) were placed in a glass vial with 2 mL of ethanol. Then, the mixture was irradiated with 3 W blue-LED (467 nm), stirring for 18 h. Purification was performed by flash column chromatography affording 3l as a yellow solid (31.3 mg, 73% yield). ^1^H NMR (400 MHz, CDCl_3_) *δ* 7.64 (d, *J* = 7.9 Hz, 1H), 7.42–7.36 (m, 1H), 7.36–7.27 (m, 2H), 7.24 (d, *J* = 1.0 Hz, 2H), 7.21–7.16 (m, 1H), 7.15–7.07 (m, 3H), 3.86 (s, 3H). ^13^C NMR (101 MHz, CDCl_3_) *δ* 137.66, 135.74, 134.35, 133.99, 132.72, 129.54, 129.40, 129.05, 128.79, 125.66, 122.59, 120.65, 120.57, 109.65, 33.18. ^77^Se NMR (76 MHz, CDCl_3_) *δ* 208.2.

#### 1-(Bu*t*-3-en-1-yl)-3-(phenylselanyl)-1*H*-indole (3m)

Following the general procedure for the reaction in one step 1-allyl indole (23.58 mg, 0.15 mmol) and diphenyl diselenide (31.4 mg, 0.075 mmol) were placed in a glass vial with 2 mL of ethanol. Next, the mixture was irradiated with 3 W blue-LED (467 nm), stirring for 18 h. Purification was performed by flash column chromatography affording 3m as a yellow oil (34.3 mg, 70% yield). ^1^H NMR (400 MHz, CDCl_3_) *δ* 7.64 (d, *J* = 7.9 Hz, 1H), 7.45–7.36 (m, 2H), 7.29 (d, *J* = 7.2 Hz, 1H), 7.24–7.16 (m, 2H), 7.16–7.06 (m, 2H), 5.80 (ddt, *J* = 13.7, 9.6, 6.9 Hz, 1H), 5.14–5.01 (m, 2H), 4.25 (t, *J* = 7.1 Hz, 2H), 2.63 (q, *J* = 7.0 Hz, 2H). ^13^C NMR (101 MHz, CDCl_3_) *δ* 136.85, 134.85, 134.37, 134.03, 132.68, 131.00, 129.40, 129.03, 128.65, 125.60, 122.52, 120.79, 120.58, 118.00, 109.83, 96.27, 46.38, 34.55. ^77^Se NMR (76 MHz, CDCl_3_) *δ* 208.04. HRMS EI [M^+^] calcd for C_18_H_17_NNaSe: 350.0419, found 350.0430.

#### *N*,*N*-Dimethyl-4-(phenylselanyl)aniline (5a)^25^

Following the general procedure for the reaction in one step, *N*,*N*-dimethylaniline (18.2 mg, 0.15 mmol) and diphenyl diselenide (31.4 mg, 0.075 mmol) were placed in a glass vial with 2 mL of ethanol. Next, the mixture was irradiated with 3 W blue-LED (467 nm), stirring for 18 h. Purification was performed by flash column chromatography affording 3h as a yellow solid (16.6 mg, 40% yield). ^1^H NMR (400 MHz, CDCl_3_) *δ* 7.65–7.40 (m, 5H), 7.24 (dd, *J* = 5.4, 3.0 Hz, 4H), 3.80 (s, 6H). ^13^C NMR (101 MHz, CDCl_3_) *δ* 151.84, 136.50, 113.49, 108.04, 104.72, 40.26. ^77^Se NMR (76 MHz, CDCl_3_) *δ* 302.5.

#### 2-(Phenylselanyl)benzene-1,3,5-triol (5b)

Following the general procedure for the reaction in one step, benzene-1,3,5-triol (9.46 mg, 0.15 mmol) and diphenyl diselenide (31.4 mg, 0.075 mmol) were placed in a glass vial with 2 mL of ethanol. Next, the mixture was irradiated with 3 W blue-LED (467 nm), stirring for 18 h. Purification was performed by flash column chromatography affording 5b as a white solid (32.1 mg, 76% yield). ^1^H NMR (400 MHz, CDCl_3_) *δ* 7.32–7.25 (m, 2H), 7.20–7.15 (m, 2H), 6.28 (s, 2H), 6.25–6.14 (m, 3H), 5.08 (s, 1H). ^13^C NMR (101 MHz, CDCl_3_) *δ* 160.29, 158.59, 133.77, 129.90, 129.54, 128.78, 127.39, 94.95. ^77^Se NMR (76 MHz, CDCl_3_) *δ* 89.90. HRMS EI [M^+^] calcd for C_12_H_10_O_3_Se: 280.9712, found 280.9713.

#### 2,4-Bis(phenylselanyl)benzene-1,3,5-triol (5c)

Following the general procedure for the reaction in one step, benzene-1,3,5-triol (9.46 mg, 0.15 mmol) and diphenyl diselenide (62.8 mg, 0.15 mmol) were placed in a glass vial with 2 mL of ethanol. Next, the mixture was irradiated with 3 W blue-LED (467 nm), stirring for 18 h. Purification was performed by flash column chromatography affording 5c as a yellow solid (19.6 mg, 30% yield). ^1^H NMR (400 MHz, CDCl_3_) *δ* 7.35–7.15 (m, 10H), 6.77 (t, *J* = 13.5 Hz, 3H), 6.50 (d, *J* = 6.1 Hz, 1H). ^13^C NMR (101 MHz, CDCl_3_) *δ* 160.86, 159.46, 133.68, 133.59, 130.00, 129.96, 129.74, 129.59, 129.19, 127.29, 94.41, 94.10. ^77^Se NMR (76 MHz, CDCl_3_) *δ* 138.40. HRMS EI [M^+^] calcd for C_18_H_14_O_3_Se_2_: 436.9193, found 436.9160.

#### 4-Phenyl-5-(phenylselanyl)thiazol-2-amine (5d)

Following the general procedure for the reaction in one step, 4-phenylthiazol-2-amine (26.43 mg, 0.15 mmol) and diphenyl diselenide (31.4 mg, 0.075 mmol) were placed in a glass vial with 2 mL of ethanol. Next, the mixture was irradiated with 3 W blue-LED (467 nm), stirring for 18 h. Purification was performed by flash column chromatography affording 5d as a pale yellow oil (26.8 mg, 54% yield). ^1^H NMR (400 MHz, CDCl_3_) *δ* 7.78–7.71 (m, 3H), 7.39–7.30 (m, 5H), 7.28–7.15 (m, 4H), 5.52 (s, 1H). ^13^C NMR (101 MHz, CDCl_3_) *δ* 170.53, 156.74, 134.48, 133.60, 129.51, 129.28, 129.10, 128.55, 128.18, 126.76, 126.39. ^77^Se NMR (76 MHz, CDCl_3_) *δ* 279.47. HRMS EI [M^+^] calcd for C_15_H_13_N_2_SSe: 332.9959, found 332.9960.

#### (1-Ethoxy-1-(4-methoxyphenyl)propan-2-yl)(phenyl)selane (5e)

Following the general procedure for the reaction in one step, *trans*-anetole (22.2 mg, 0.15 mmol) and diphenyl diselenide (31.4 mg, 0.075 mmol) were placed in a glass vial with 2 mL of ethanol. Next, the mixture was irradiated with 3 W blue-LED (467 nm), stirring for 18 h. Purification was performed by flash column chromatography affording 3e as a pale yellow oil (29.3 mg, 56% yield). ^1^H NMR (400 MHz, CDCl_3_) *δ* 7.56–7.43 (m, 12H), 7.29 (d, *J* = 4.5 Hz, 6H), 7.26–7.15 (m, 22H), 6.84 (t, *J* = 8.1 Hz, 11H), 4.45 (d, *J* = 5.0 Hz, 4H), 3.80 (s, 17H), 3.55–3.28 (m, 13H), 1.93–1.80 (m, 4H), 1.37 (d, *J* = 7.0 Hz, 12H), 1.19 (t, *J* = 7.0 Hz, 14H). ^13^C NMR (101 MHz, CDCl_3_) *δ* 159.07, 134.46, 132.87, 132.56, 132.44, 130.34, 130.21, 129.48, 128.83, 128.16, 127.15, 126.87, 123.49, 113.91, 113.52, 84.20, 77.33, 77.01, 76.70, 64.82, 55.23, 46.06, 16.94, 15.21. ^77^Se NMR (76 MHz, CDCl_3_) *δ* 395.24. HRMS EI [M^+^] calcd for C_18_H_22_NaO_2_Se: 373.0678, found 373.0645.

## Conflicts of interest

There are no conflicts to declare.

## Supplementary Material

RA-009-C9RA03642C-s001
