# Supplementary material for: Metal- and photocatalyst-free synthesis of 3-selenylindoles and asymmetric diarylselenides promoted by visible light
Source: RSC Adv. 2019 Jul 23;9(39):22685–94. doi: 10.1039/c9ra03642c (PMC9067025; doi:10.1039/c9ra03642c)

Electronic Supplementary Material (ESI) for RSC Advances.
This journal is © The Royal Society of Chemistry 2019

Supporting Information

Metal- and Photocatalyst-free Synthesis of 3-Selenylindoles and Asymmetric Diarylselenides Promoted by Visible Light

Ignacio D. Lemir, Willber D. Castro-Godoy, Adrián A. Heredia, Luciana C. Schmidt and Juan E. Argüello

Table of contents

Table S1. Obtention of 3-chalcogenylindoles employing visible light 2
Laser flash photolysis experiments 3
Steady-state Fluorescence Study 5
Time-Resolving Fluorescence Study 6
^{1}H, ^{13}C, ^{19}F, ^{77}Se Spectra of Synthesized Products 8
3-(phenylselanyl)-1*H*-indole (**3a**) 9
3-(naphthalen-2-ylselanyl)-1*H*-indole (**3b**) 12
3-((4-chlorophenyl)selanyl)-1*H*-indole (**3c**) 15
3-((4-methoxyphenyl)selanyl)-1*H*-indole (**3d**) 18
3-((4-(trifluoromethyl)phenyl)selanyl)-1*H*-indole (**3e**) 21
3-((4-methylselanyl)-1*H*-indole (**3f**) 25
3-(benzylselanyl)-1*H*-indole (**3g**) 28
3-((4-methylbenzyl)selanyl)-1*H*-indole (**3h**) 31
3-((4-(trifluoromethyl)benzyl)selanyl)-1*H*-indole (**3i**) 34
3-((4-fluorobenzyl)selanyl)-1*H*-indole (**3j**) 38
2-methyl-3-(phenylselanyl)-1*H*-indole (**3k**). 42
1-methyl-3-(phenylselanyl)-1*H*-indole (**3l**). 45
1-(but-3-en-1-yl)-3-(phenylselanyl)-1*H*-indole (**3m**). 48
N,N-dimethyl-4-(phenylselanyl)aniline (**5a**) 51
2-(phenylselanyl)benzene-1,3,5-triol (**5b**). 54
2,4-bis(phenylselanyl)benzene-1,3,5-triol (**5c**). 57
4-phenyl-5-(phenylselanyl)thiazol-2-amine (**5d**). 60
(1-ethoxy-1-(4-methoxyphenyl)propan-2-yl)(phenyl)selane (**5e**) 63

Table S1. Obtention of 3-chalcogenylindoles employing visible light.[a]

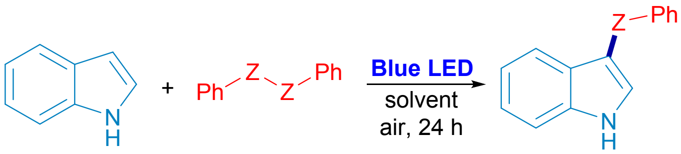

1 **2** **3**

Entry	Z	Solvent	Obtention of 3[b]
1	S	MeCN	✘
2	S	Toluene	✘
3	S	EtOH	✘
4	S	Acetone	✘
5	**Se**	**MeCN**	✓
6	**Se**	**EtOH**	✓
7	**Se**	**Acetone**	✓
8	Te	MeCN	✘
9	Te	EtOH	✘
10	Te	Acetone	✘

[a] Reaction conditions: **1** (0.15 mmol), **2** (0.75 equiv.), solvent (2 mL) at room temperature irradiated with 3 W blue LED (467 nm) for 24 h. [b] Detection of **3** by GC, where a red cross and a green thick means not detected and observed, respectively.

Laser flash photolysis experiments

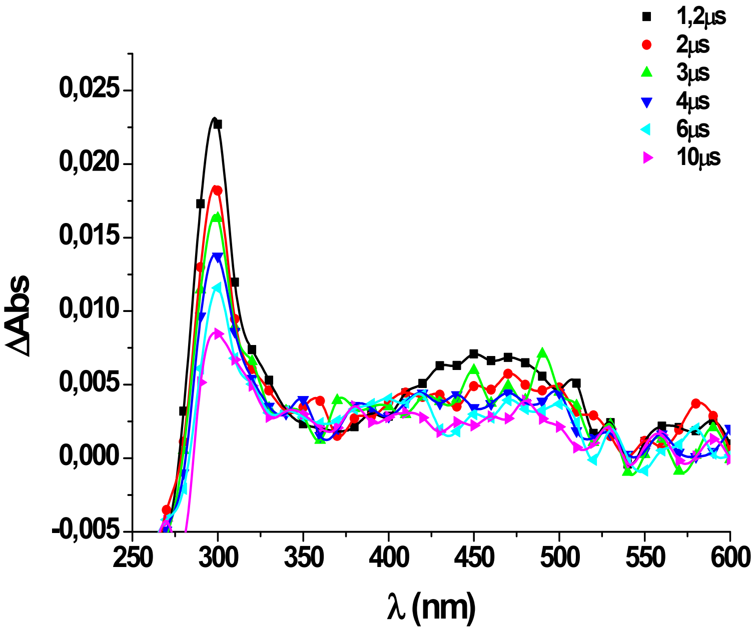

Figure S1. Transient absorption spectrum of Ph_2Se_2 (0.1 mM) excitation at 266 nm in MeCN. Spectrum taken at 1.2, 2, 3, 4, 6, 10 μs after the laser pulse.

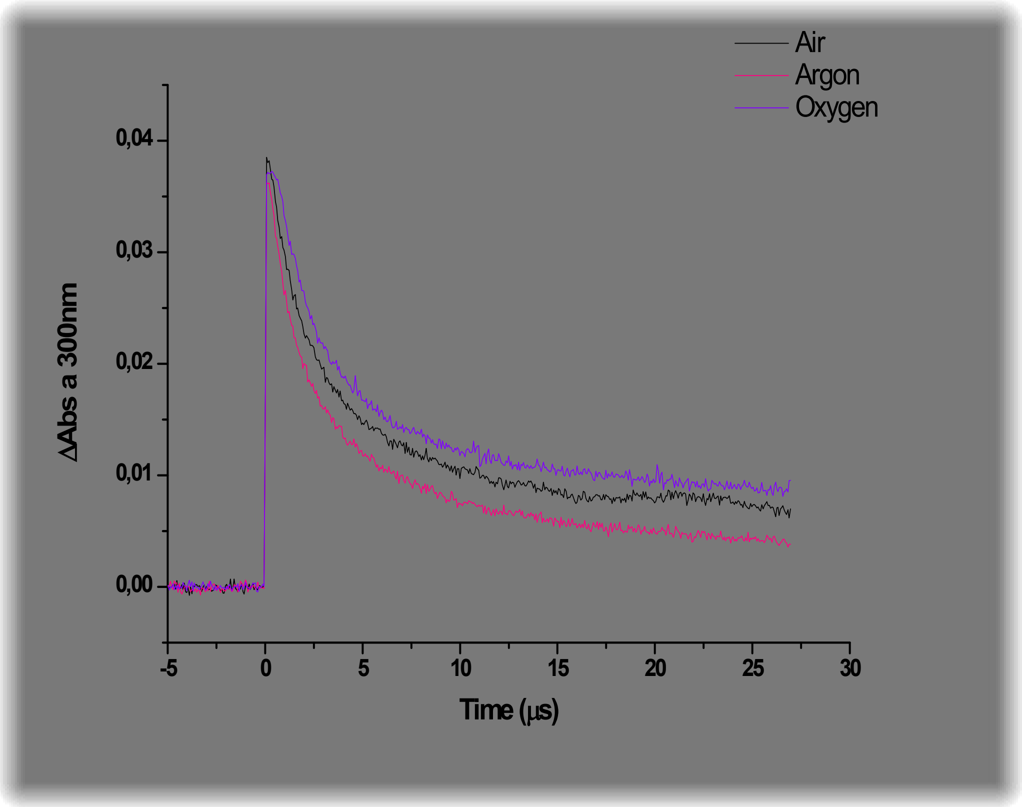

Figure S2. Decay trace monitored at 300 nm under air ( ), argon ( ), oxygen ( ) atmosphere excitation at 266nm in MeCN

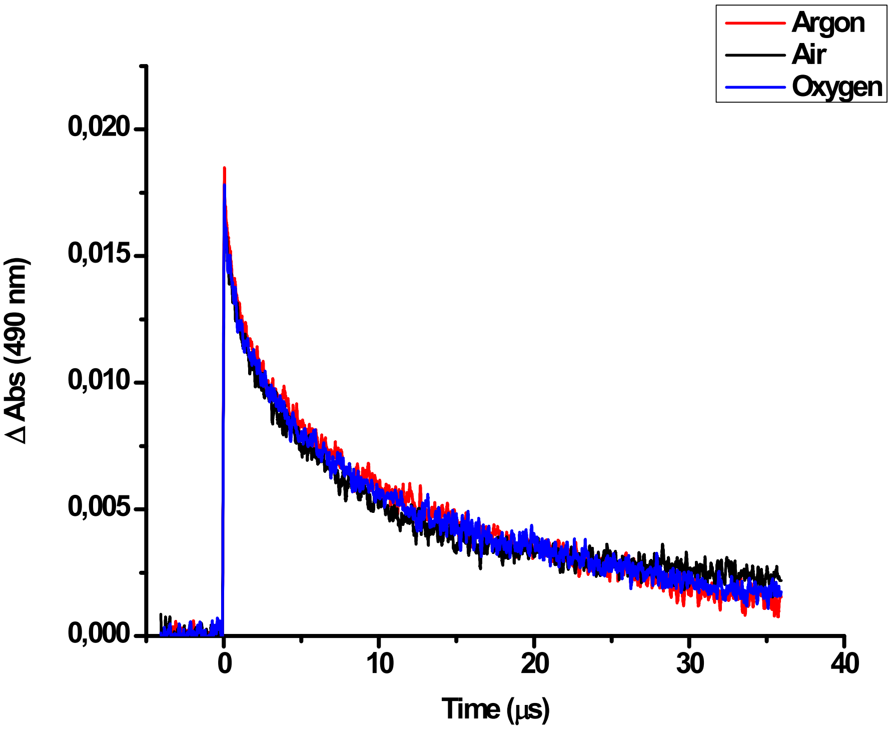

Figure S3. Decay trace monitored at 490 nm under air ( ), argon ( ), oxygen ( ) atmosphere excitation at 355nm in MeOH.

Table S2. Decay profile of photogenerated PhSe˙ radical monitored at 490 nm.

$y = A_1 e^{(-x/\tau_1)} + y_0$						
**Conditions**	**τ1 (μs)**	**Error (τ_1)**	**A_1**	**Error (A_1)**	**y_0**	**Error (y_0)**
**Argon**	**9.79**	**0.09**	**0.01217**	**0.0004**	**0.00154**	**0.00003**
**Air**	**6.69**	**0.06**	**0.01126**	**0.0005**	**0.00256**	**0.00002**
**Oxygen**	**8.74**	**0.09**	**8.74575**	**0.0004**	**0.00196**	**0.00002**

According to literature,[1] quenching of phenyl selenyl radical in presences of oxygen was not observed, since selenyl radical life time (τ_1) did not significantly change at different atmospheres.

1. Alam, M. M., Ito, O., Koga, Y. & Ouchi, A. *Int. J. Chem. Kinet.* 1998, **30**, 193–200.

Steady-state Fluorescence Study

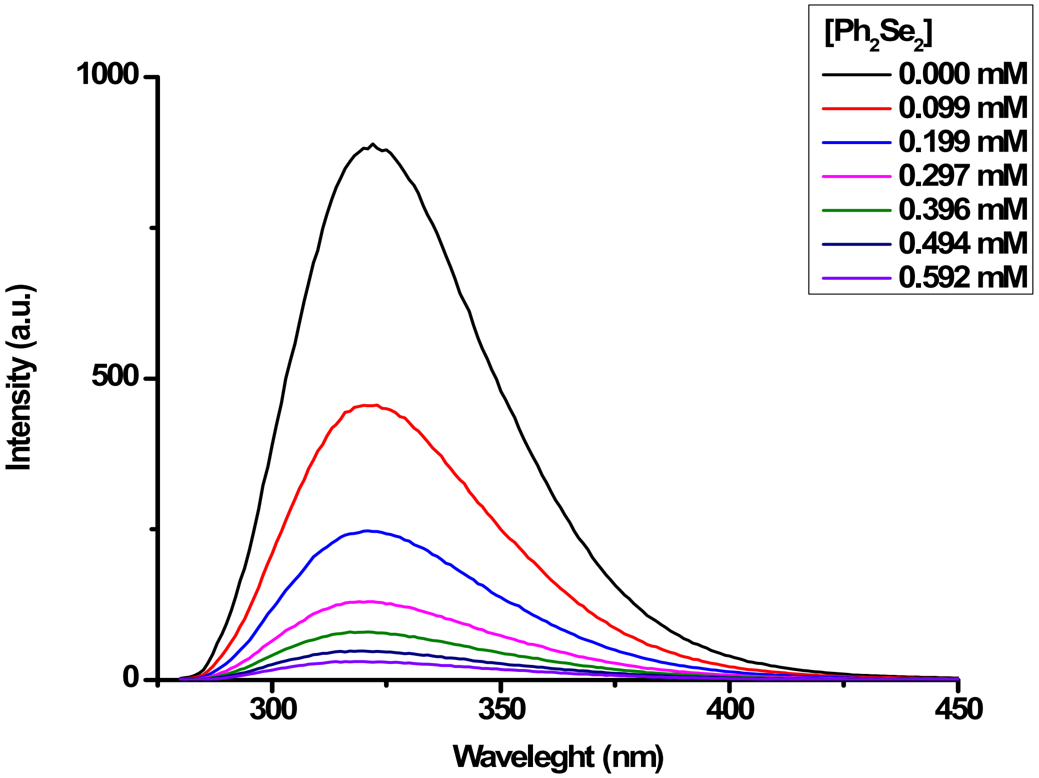

Figure S4. Indole fluorescence quenching with different concentrations of Ph_2Se_2 in MeCN. *[Indole] = 0.01 mM.*

Time-Resolving Fluorescence Study

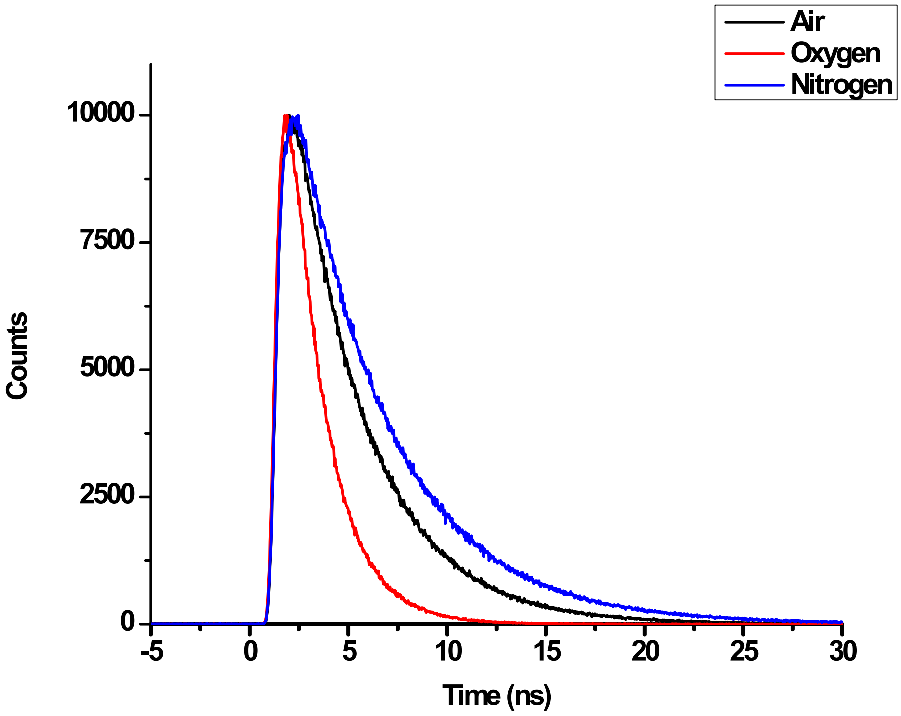

Figure S5. Time-resolved fluorescence of indole under different atmospheres: air (-), oxygen (-) and nitrogen (-).

Table S3. Decay profile of fluorescence lifetime of indole under different atmospheres.

$y = A_1 e^{\left(-x/\tau_1\right)} + y_0$						
**Conditions**	**τ1 (ns)**	**Error (τ_1)**	**A_1**	**Error (A_1)**	**y_0**	**Error (y_0)**
**Nitrogen**	3.85	0.01	18279	40	-14	4
**Air**	3.78	0.01	18806	31	-5	3
**Oxygen**	2.03	0.01	27573	165	-38	7

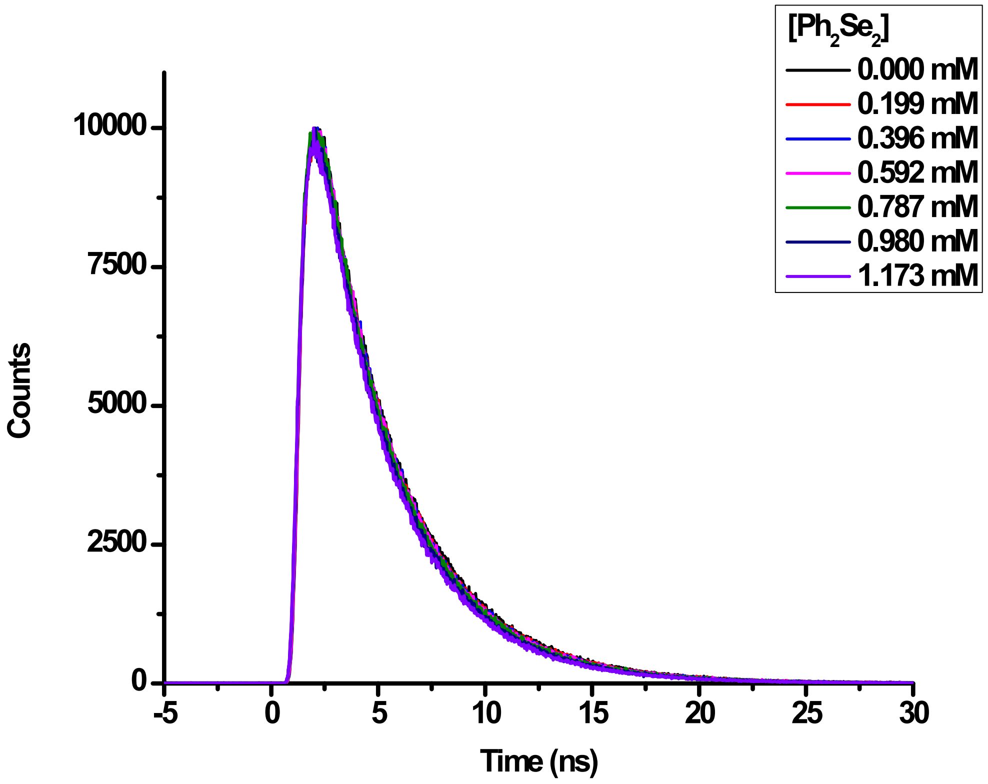

Figure S6. Time-resolved fluorescence of indole with different concentrations of Ph_2Se_2 in MeCN. *[Indole]=0.01 mM.*

^{1}H, ^{13}C, ^{19}F, ^{77}Se Spectra of Synthesized Products

^{1}H NMR - 3-(phenylselanyl)-1H-indole (3a)

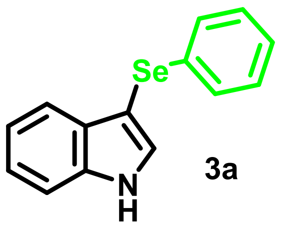

3a

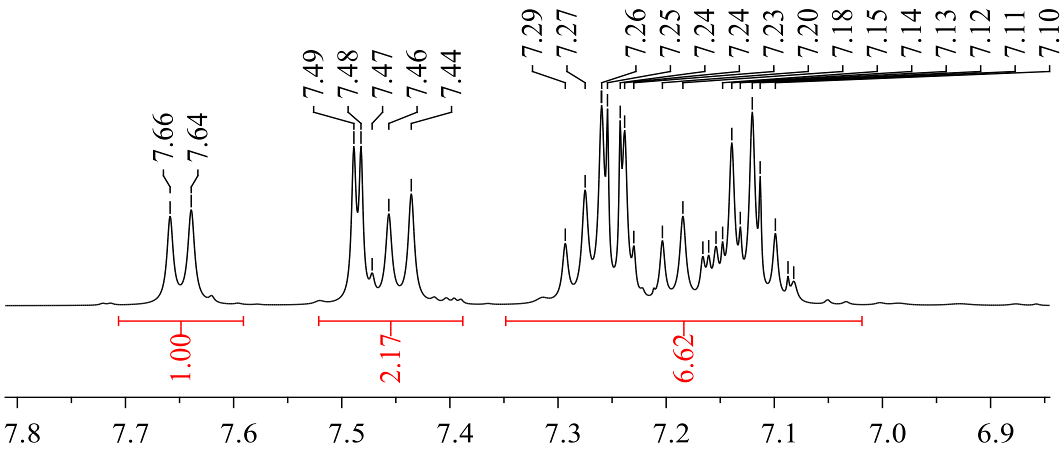

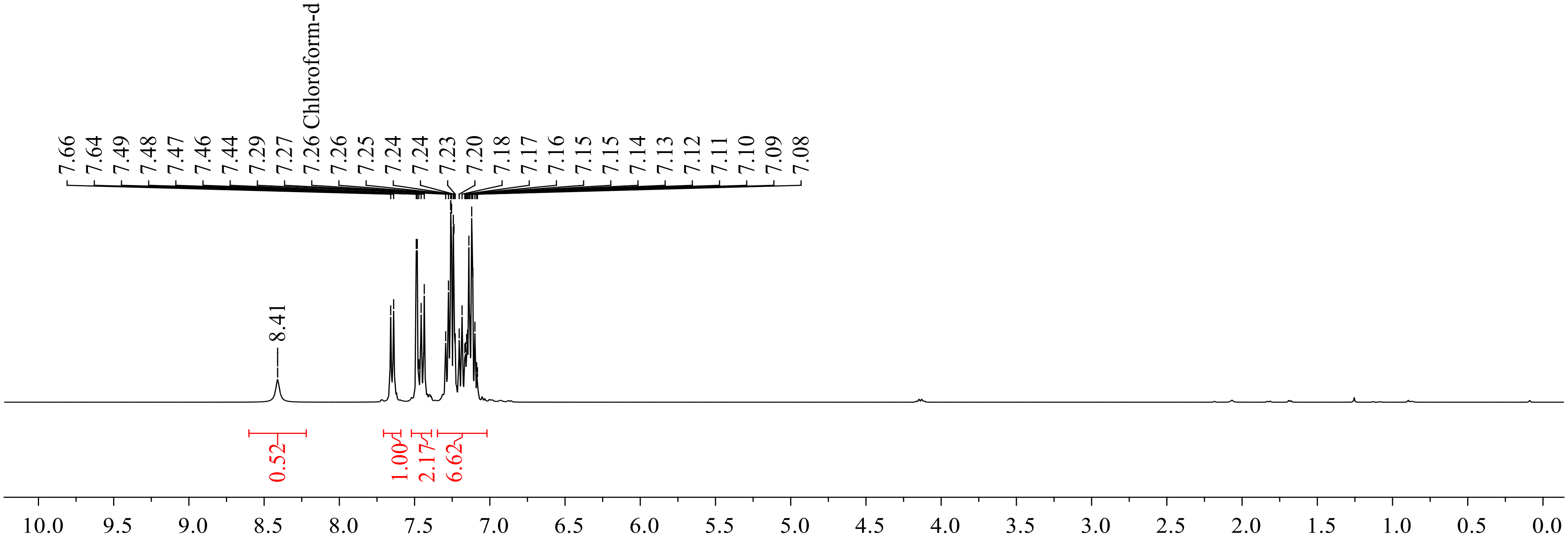

^{13}C NMR - 3-(phenylselanyl)-1H-indole (3a)

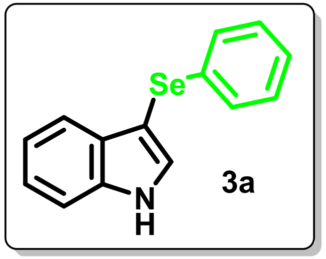

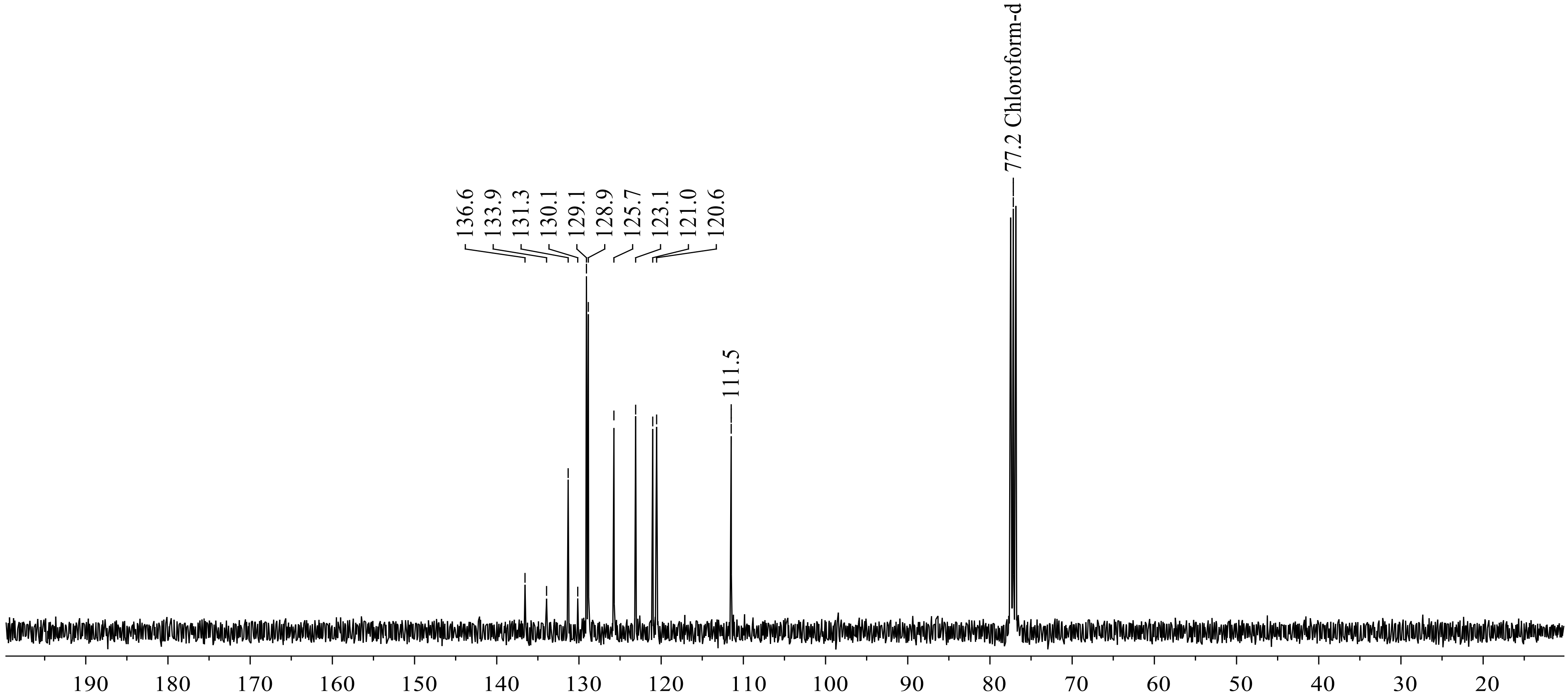

^{77}Se NMR - 3-(phenylselanyl)-1H-indole (3a)

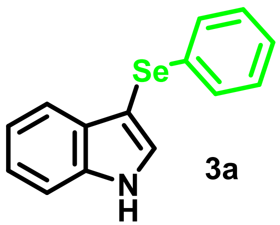

3a

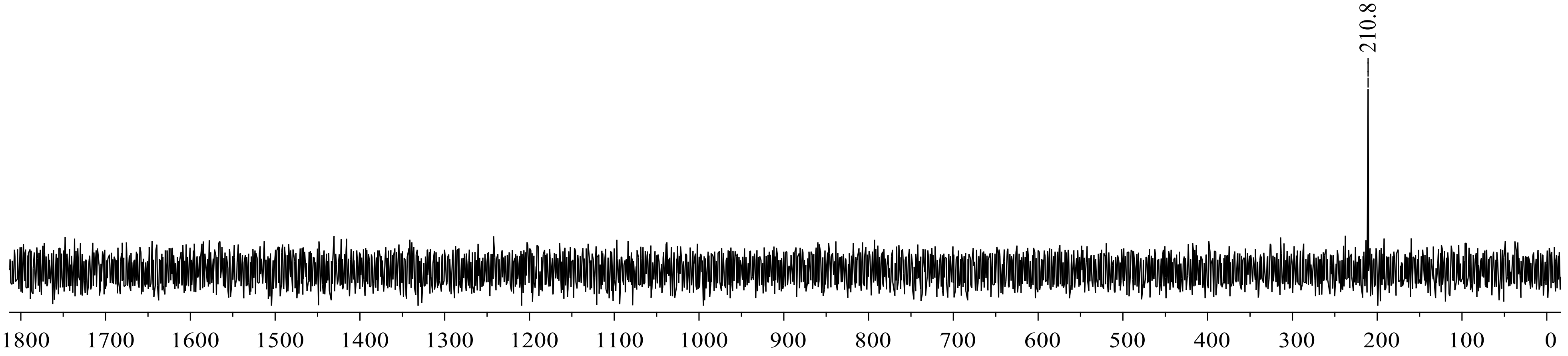

^{1}H NMR - 3-(naphthalen-2-ylselanyl)-1H-indole (3b)

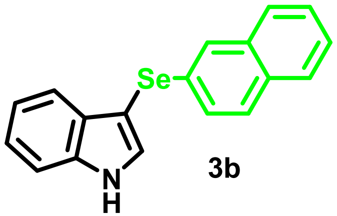

3b

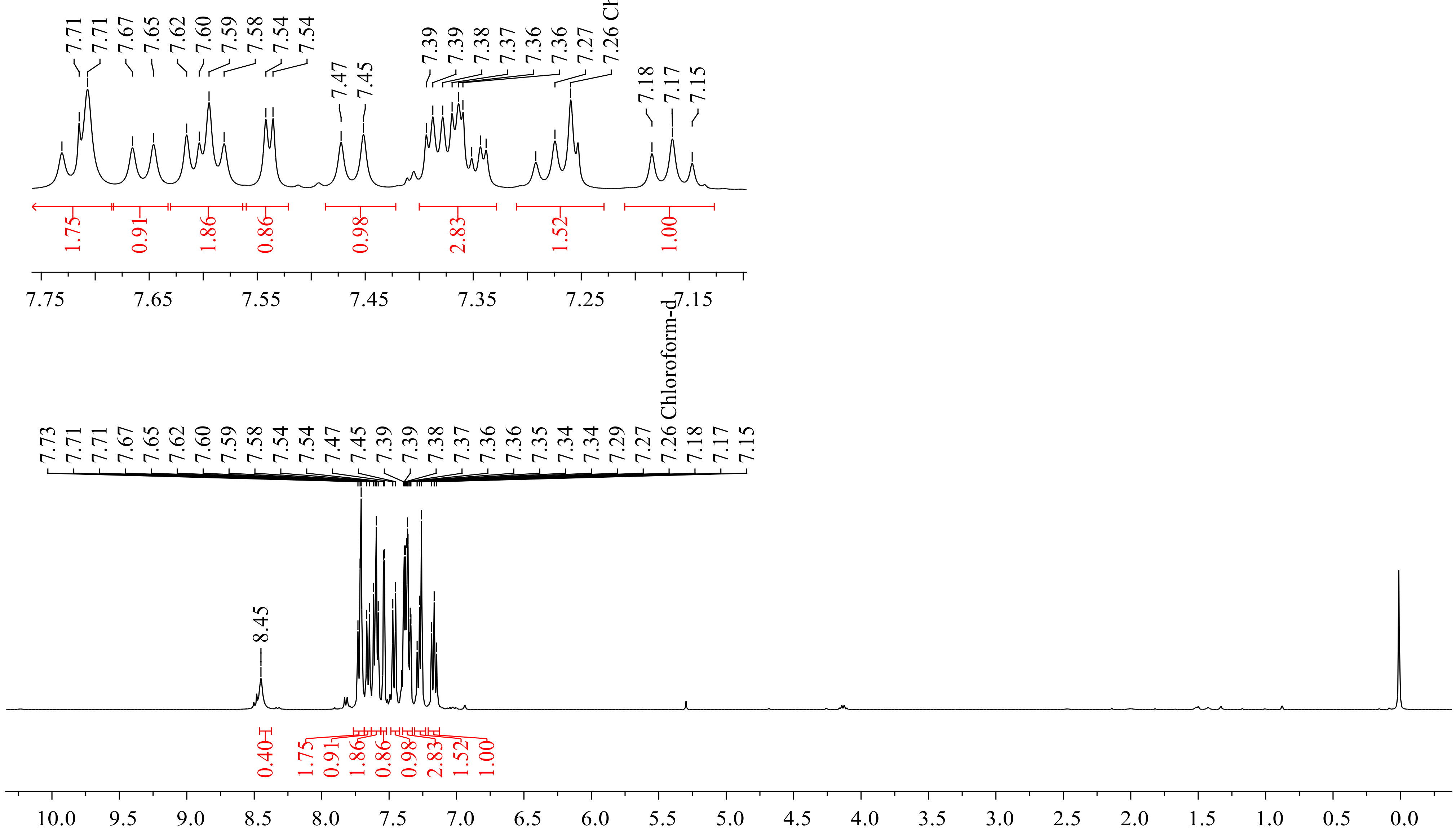

^{13}C NMR - 3-(naphthalen-2-ylselanyl)-1H-indole (3b)

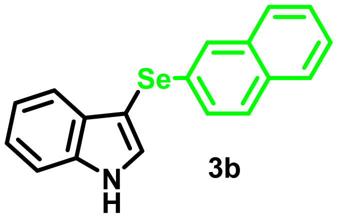

3b

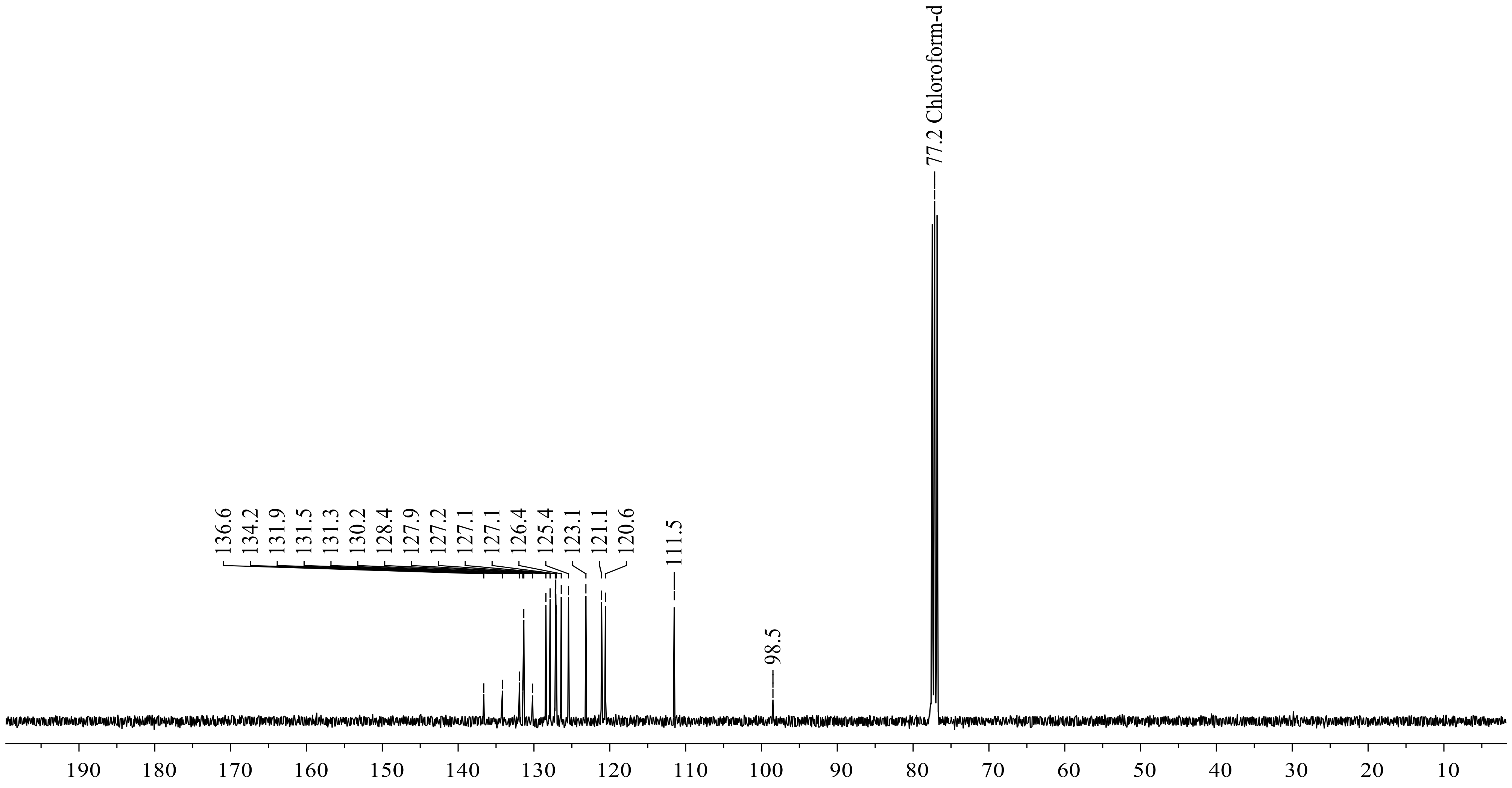

^{77}Se NMR - 3-(naphthalen-2-ylselanyl)-1H-indole (3b)

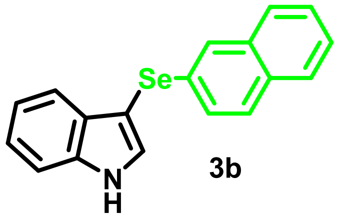

3b

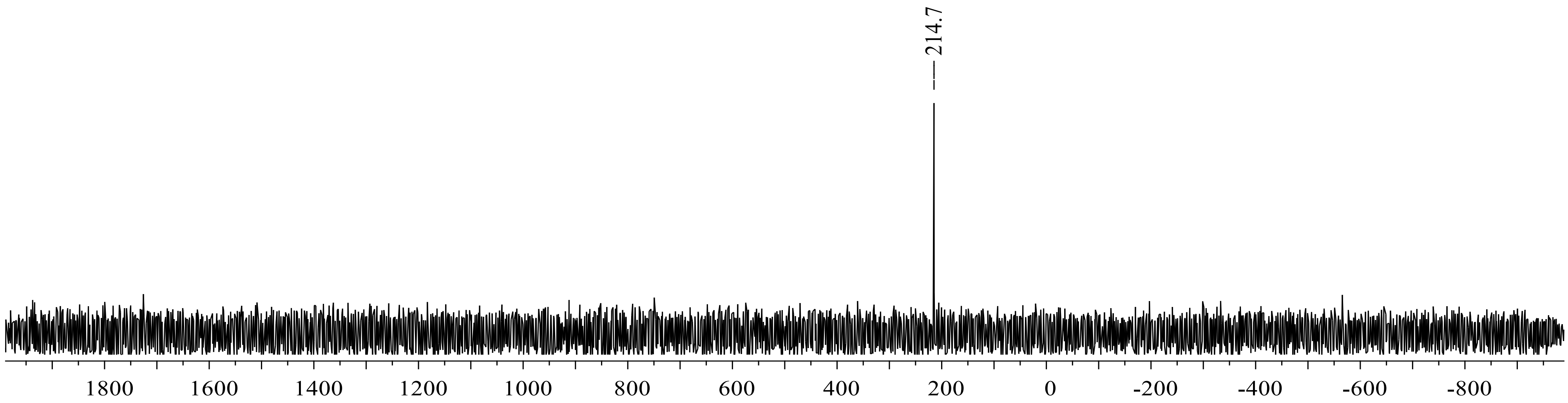

^{1}H NMR - 3-((4-chlorophenyl)selanyl)-1H-indole (3c)

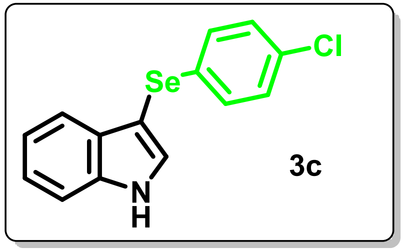

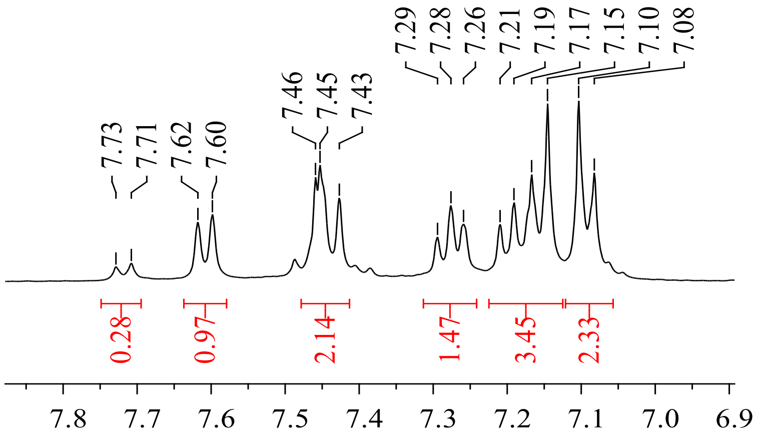

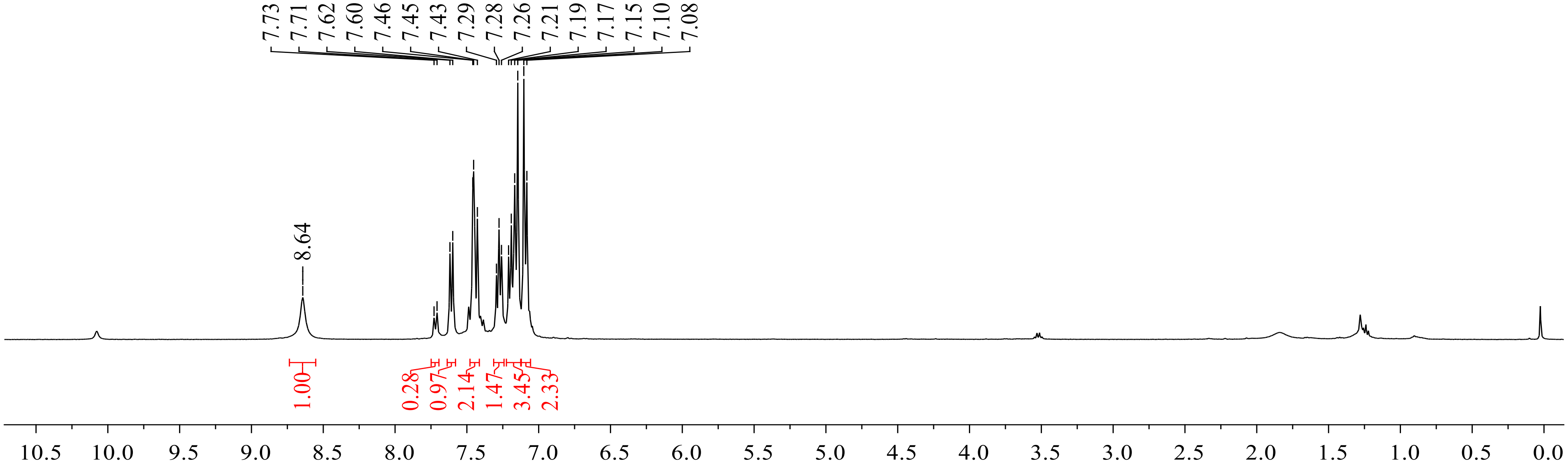

^{13}C NMR - 3-((4-chlorophenyl)selanyl)-1H-indole (3c)

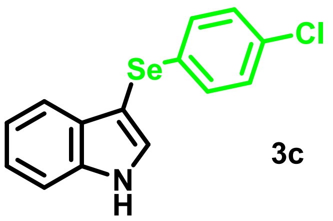

3c

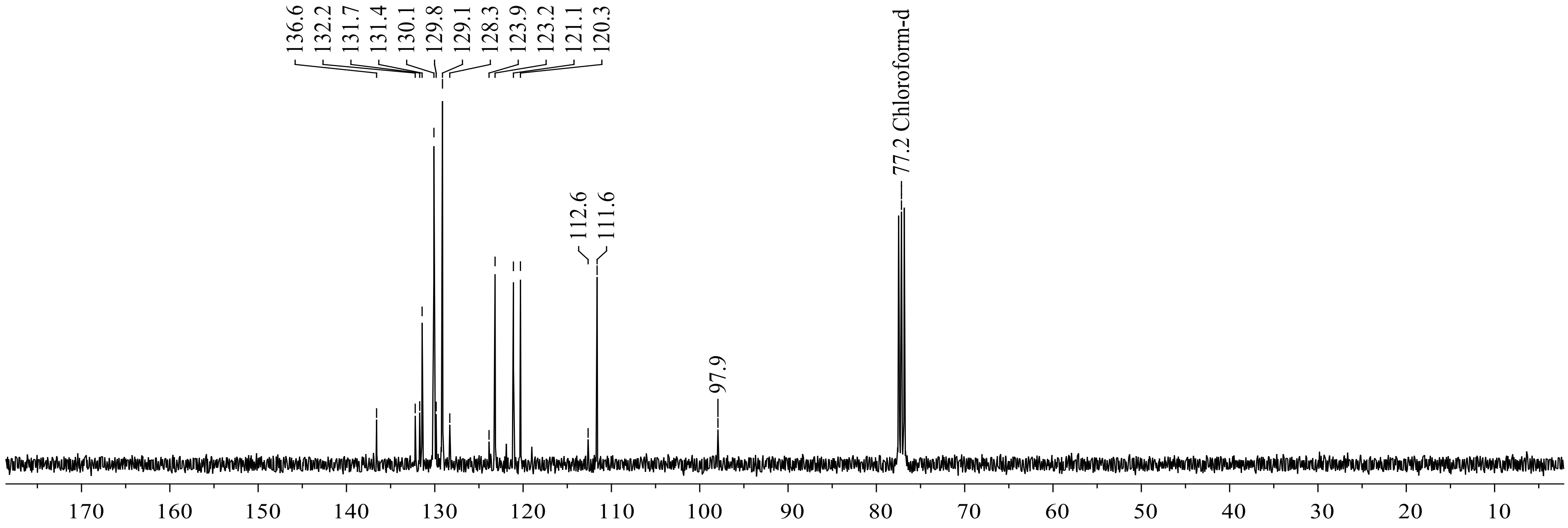

^{77}Se NMR - 3-((4-chlorophenyl)selanyl)-1H-indole (3c)

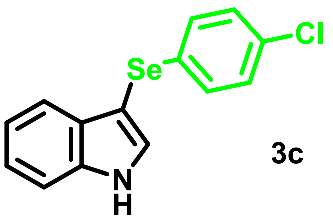

3c

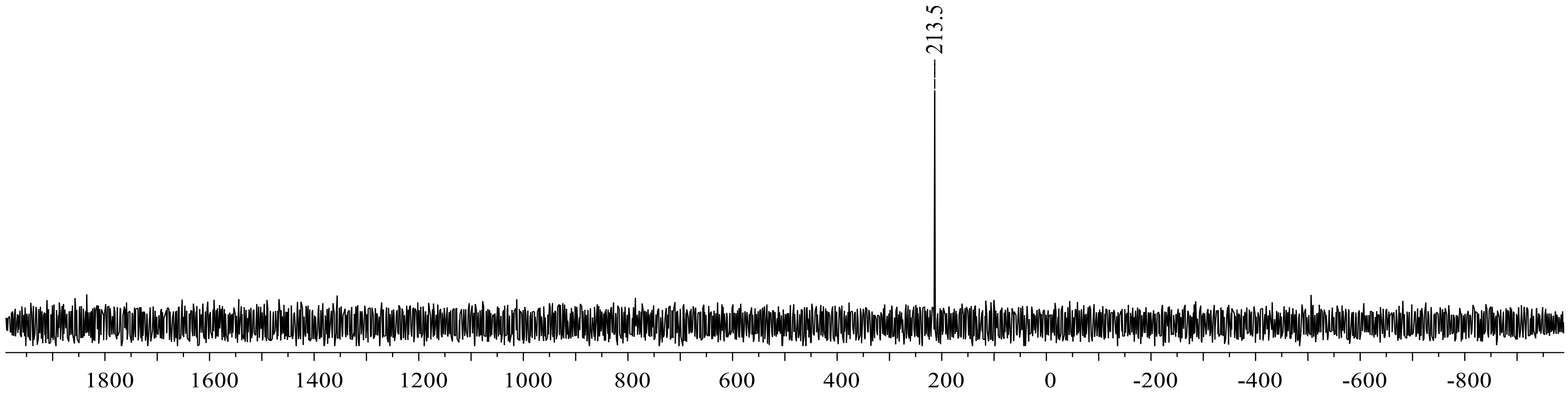

^{1}H NMR - 3-((4-methoxyphenyl)selanyl)-1H-indole (3d)

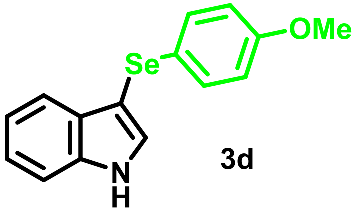

3d

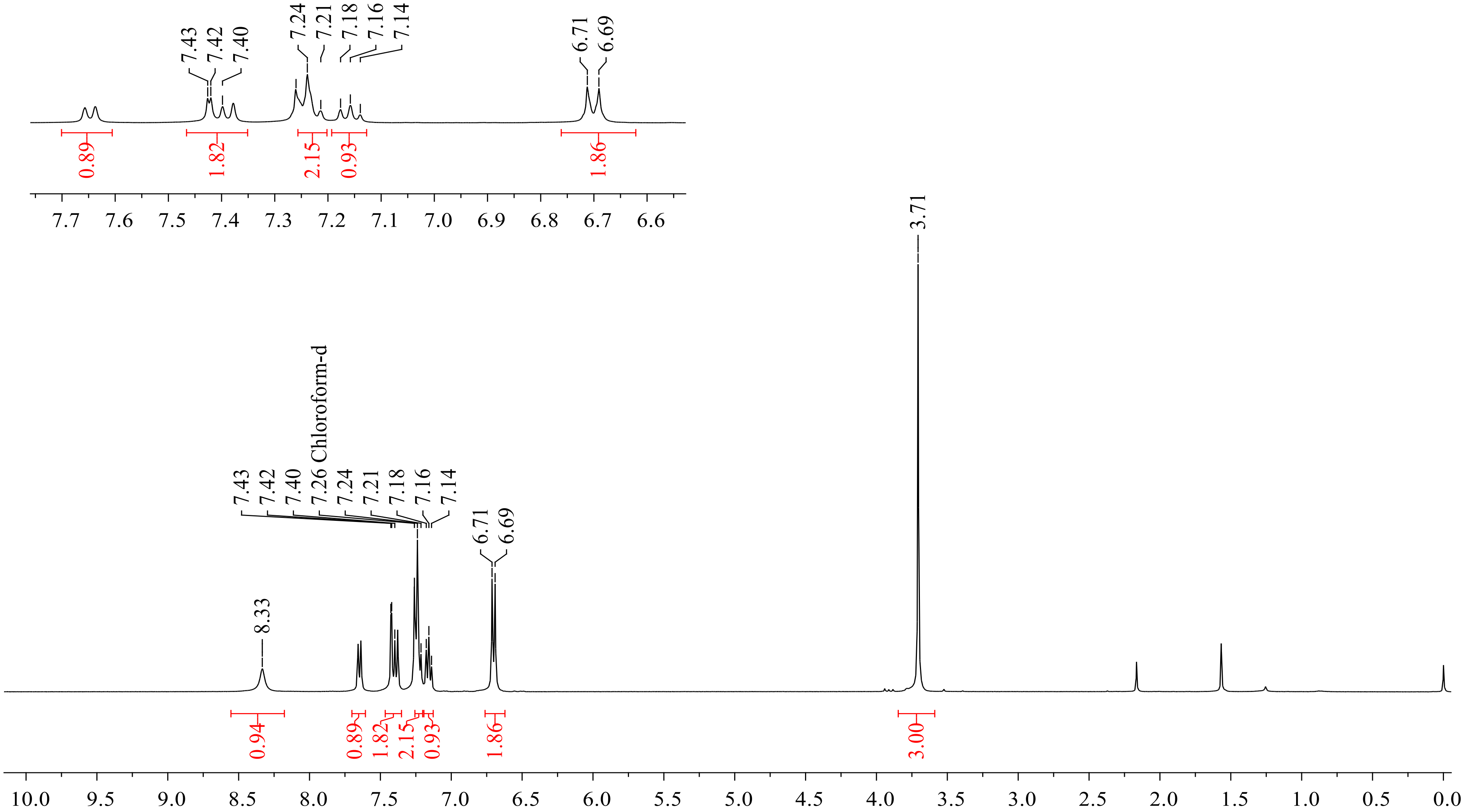

^{13}C NMR - 3-((4-methoxyphenyl)selanyl)-1H-indole (3d)

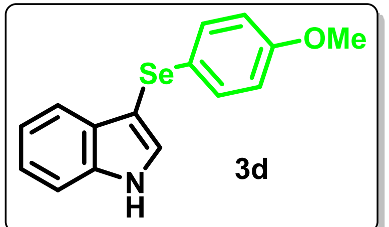

3d

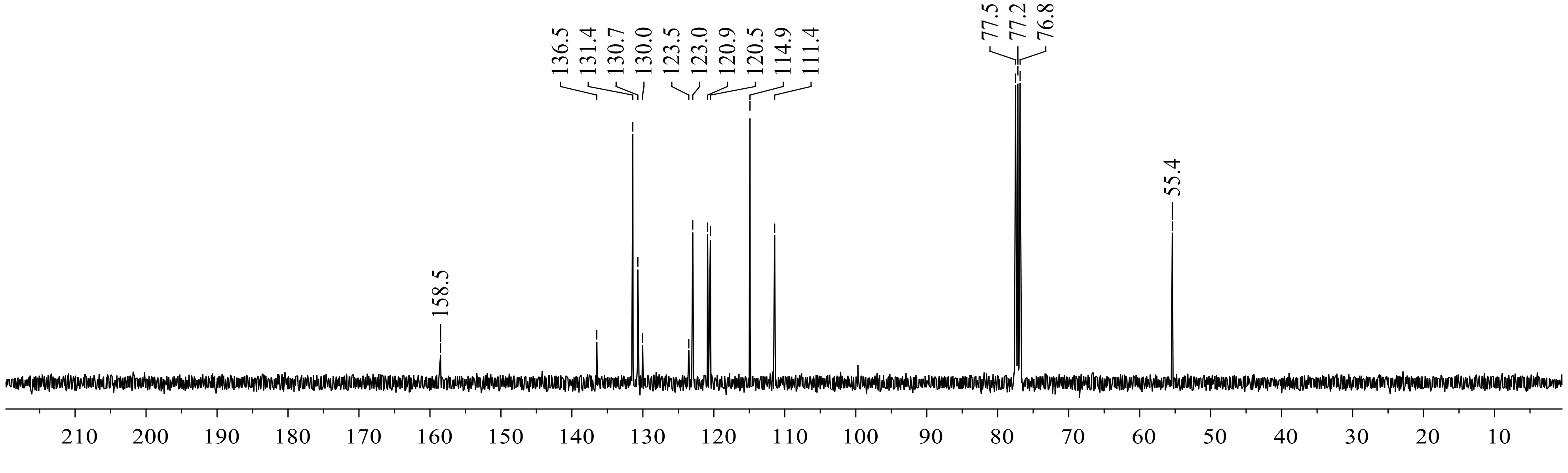

^{77}Se NMR - 3-((4-methoxyphenyl)selanyl)-1H-indole (3d)

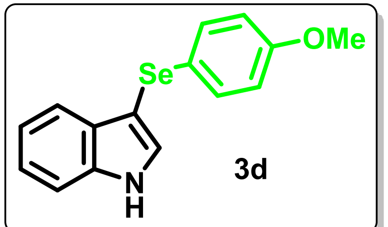

3d

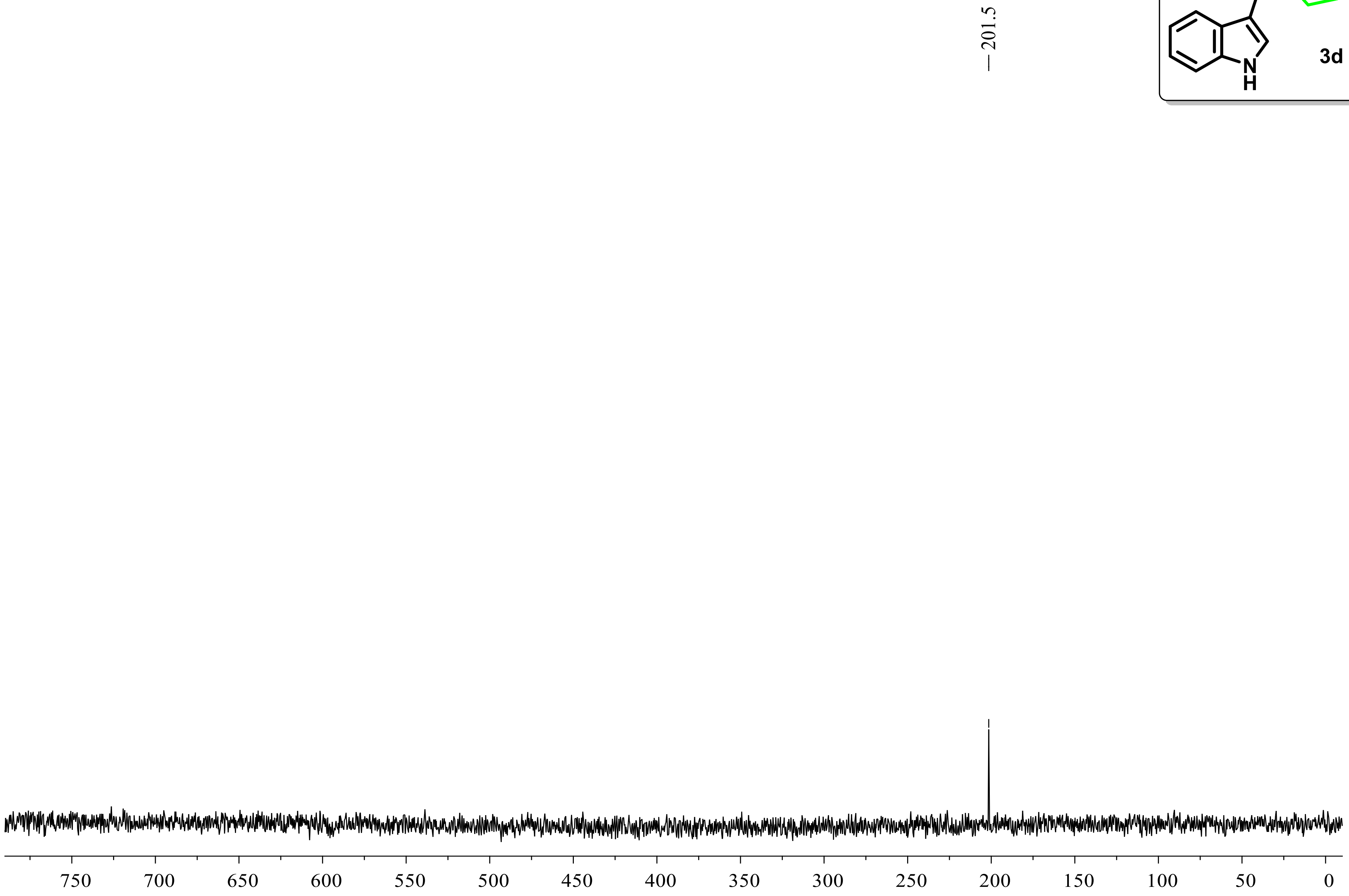

^{1}H NMR - 3-((4-(trifluoromethyl)phenyl)selanyl)-1H-indole (3e)

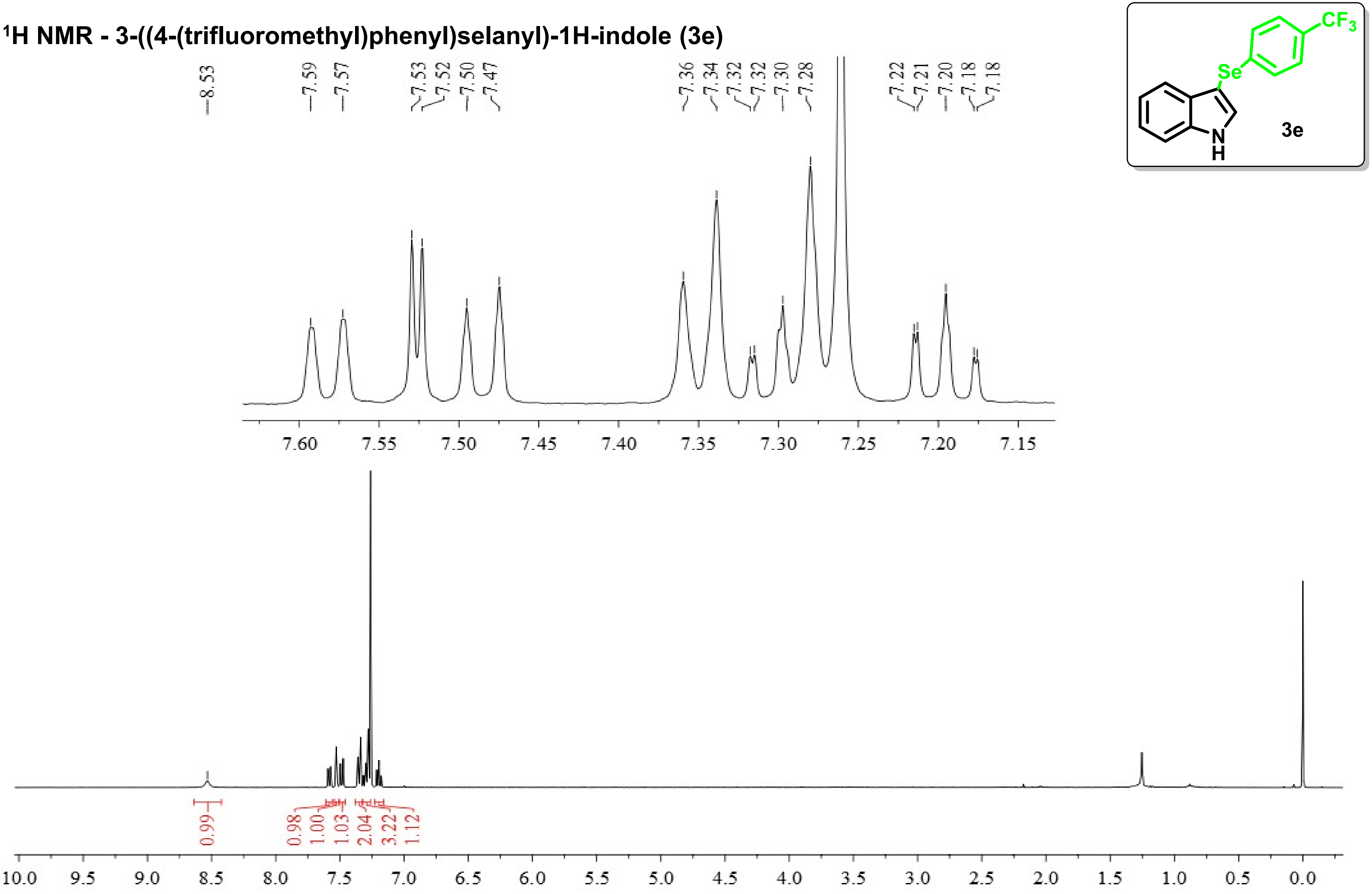

^{13}C NMR - 3-((4-(trifluoromethyl)phenyl)selanyl)-1H-indole (3e)

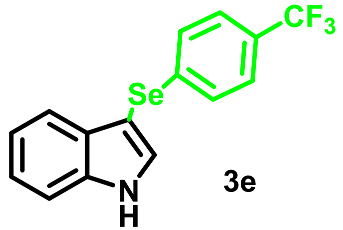

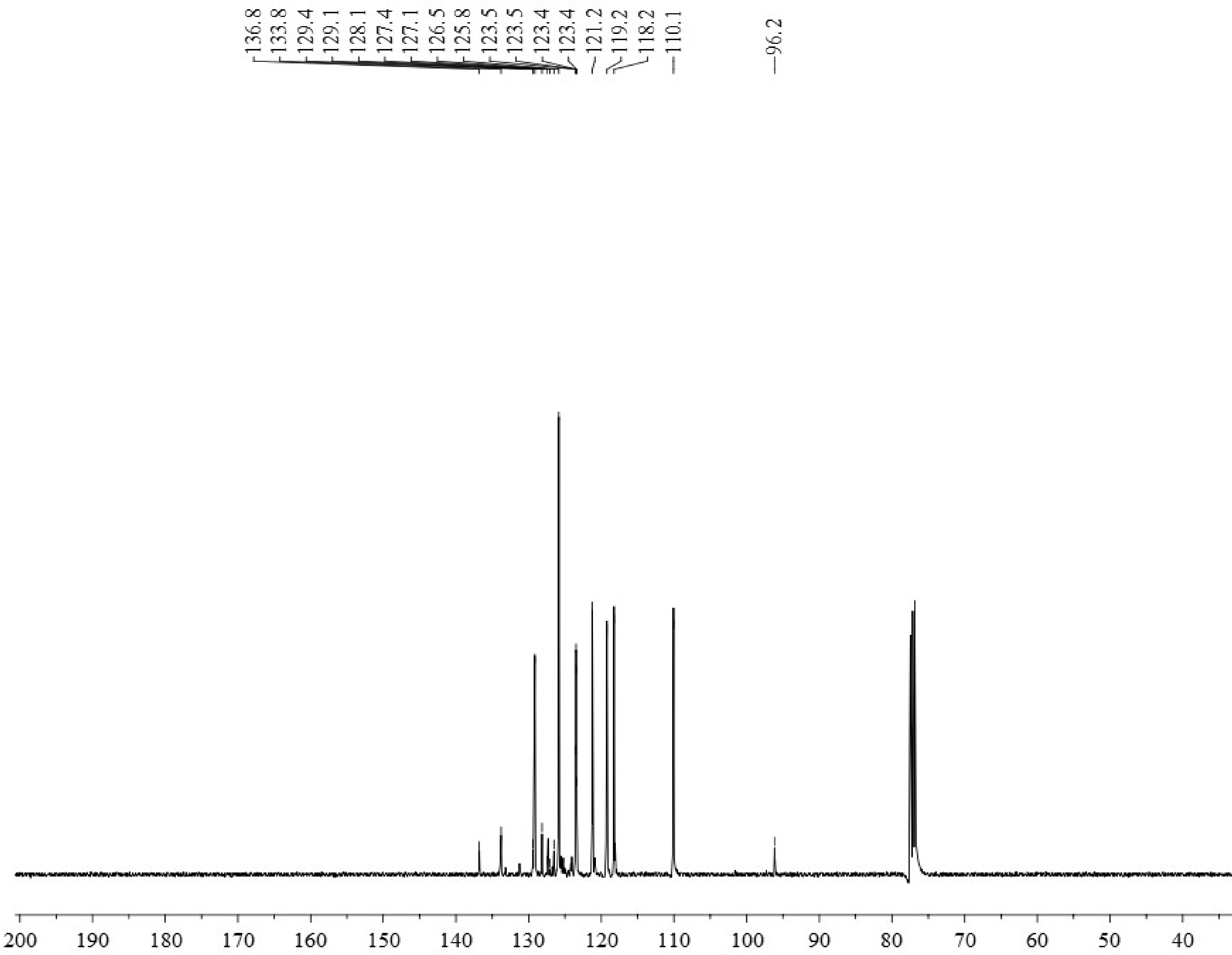

^{77}Se NMR - 3-((4-(trifluoromethyl)phenyl)selanyl)-1H-indole (3e)

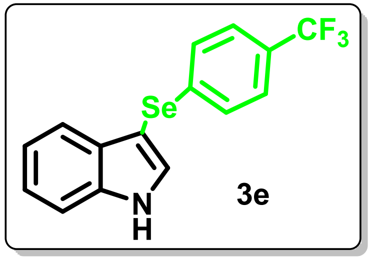

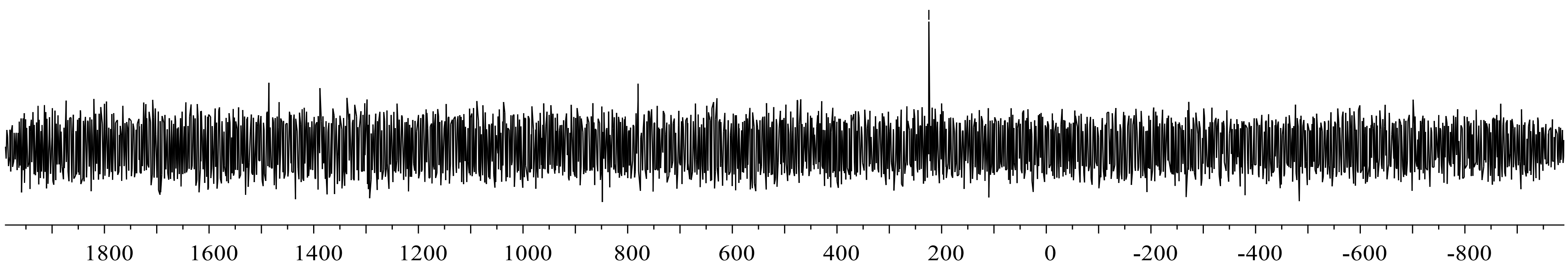

^{19}F NMR - 3-((4-(trifluoromethyl)phenyl)selanyl)-1H-indole (3e)

-62.44

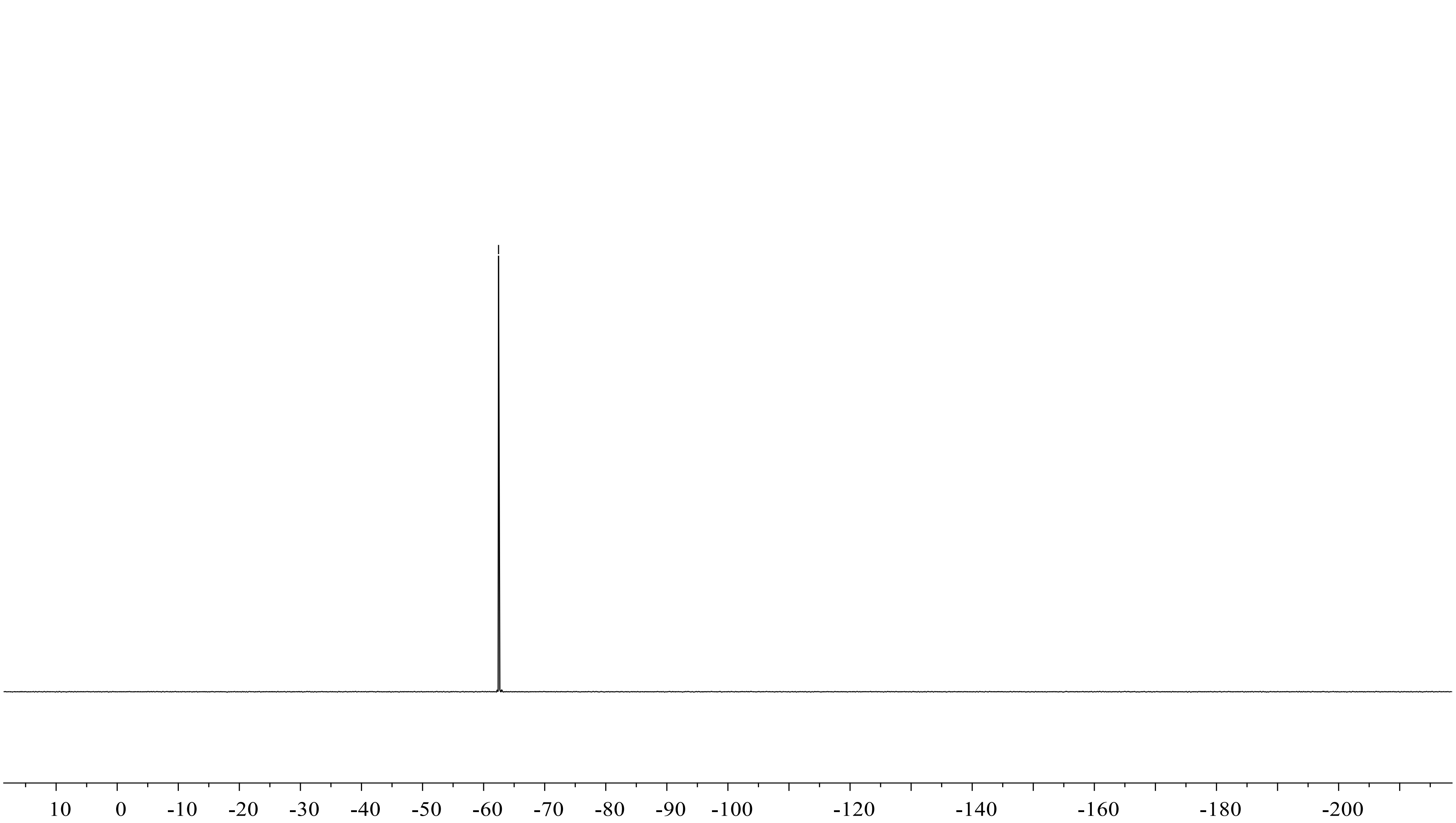

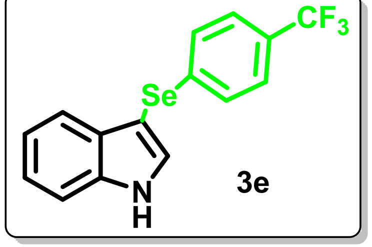

3e

^{1}H NMR - 3-((4-methylselanyl)-1H-indole (3f)

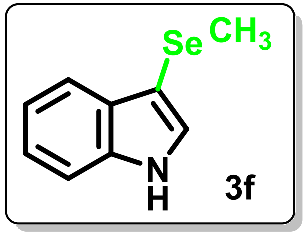

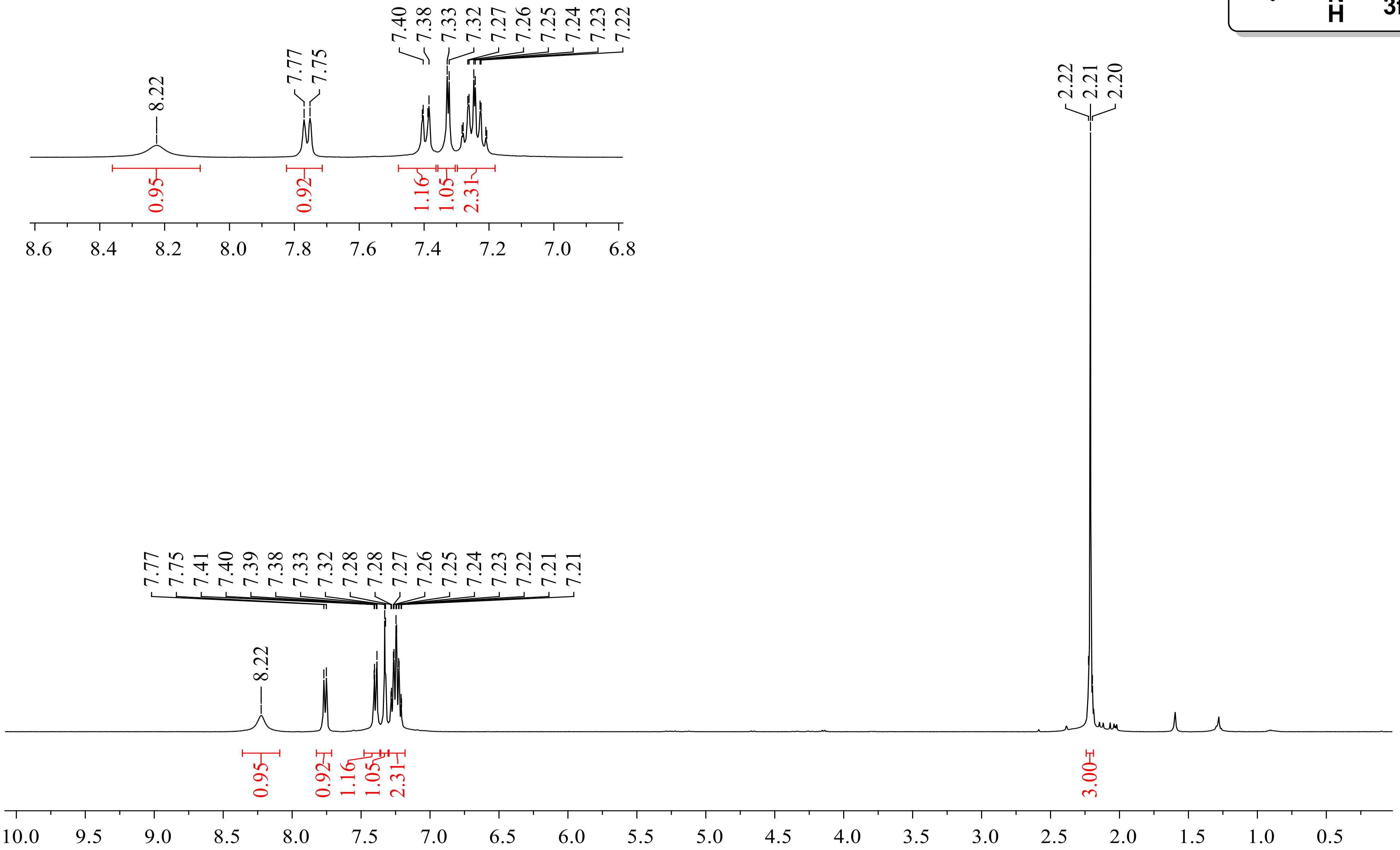

^{13}C NMR - 3-((4-methylselanyl)-1H-indole (3f)

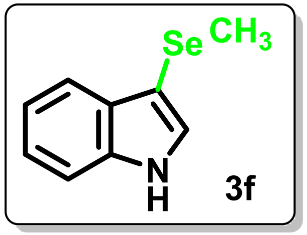

3f

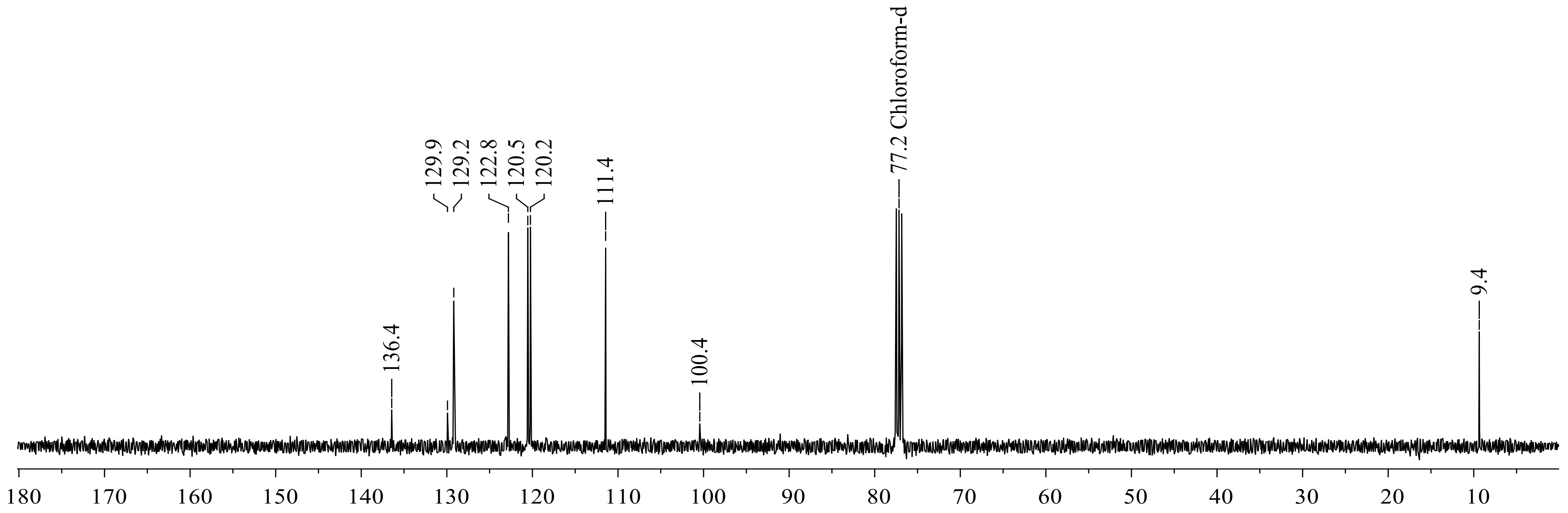

^{77}Se NMR - 3-((4-methylselanyl)-1H-indole (3f)

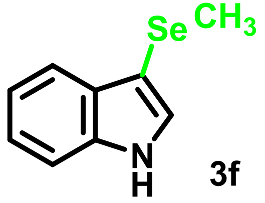

3f

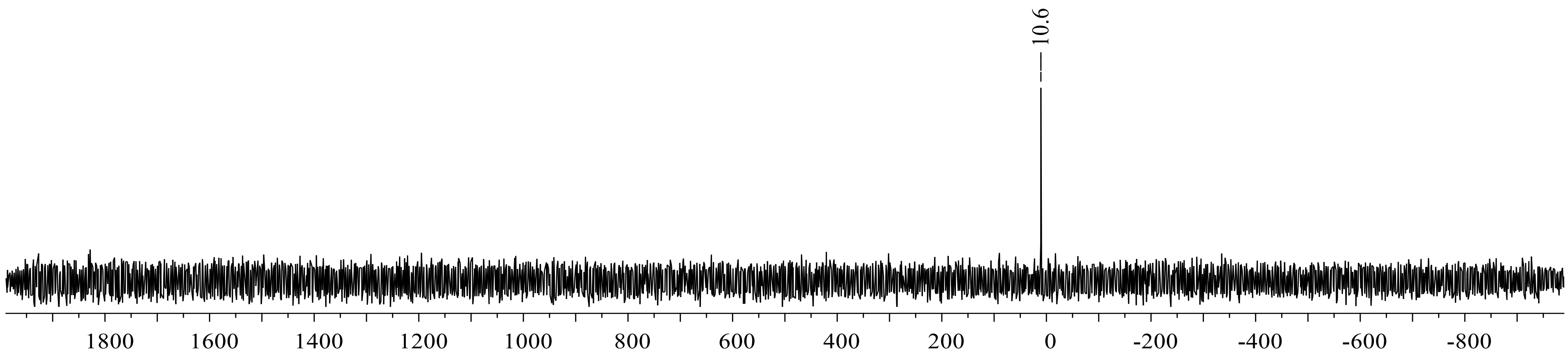

^{1}H NMR - 3-(benzylselanyl)-1H-indole (3g)

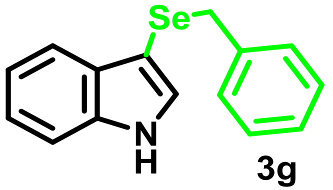

3g

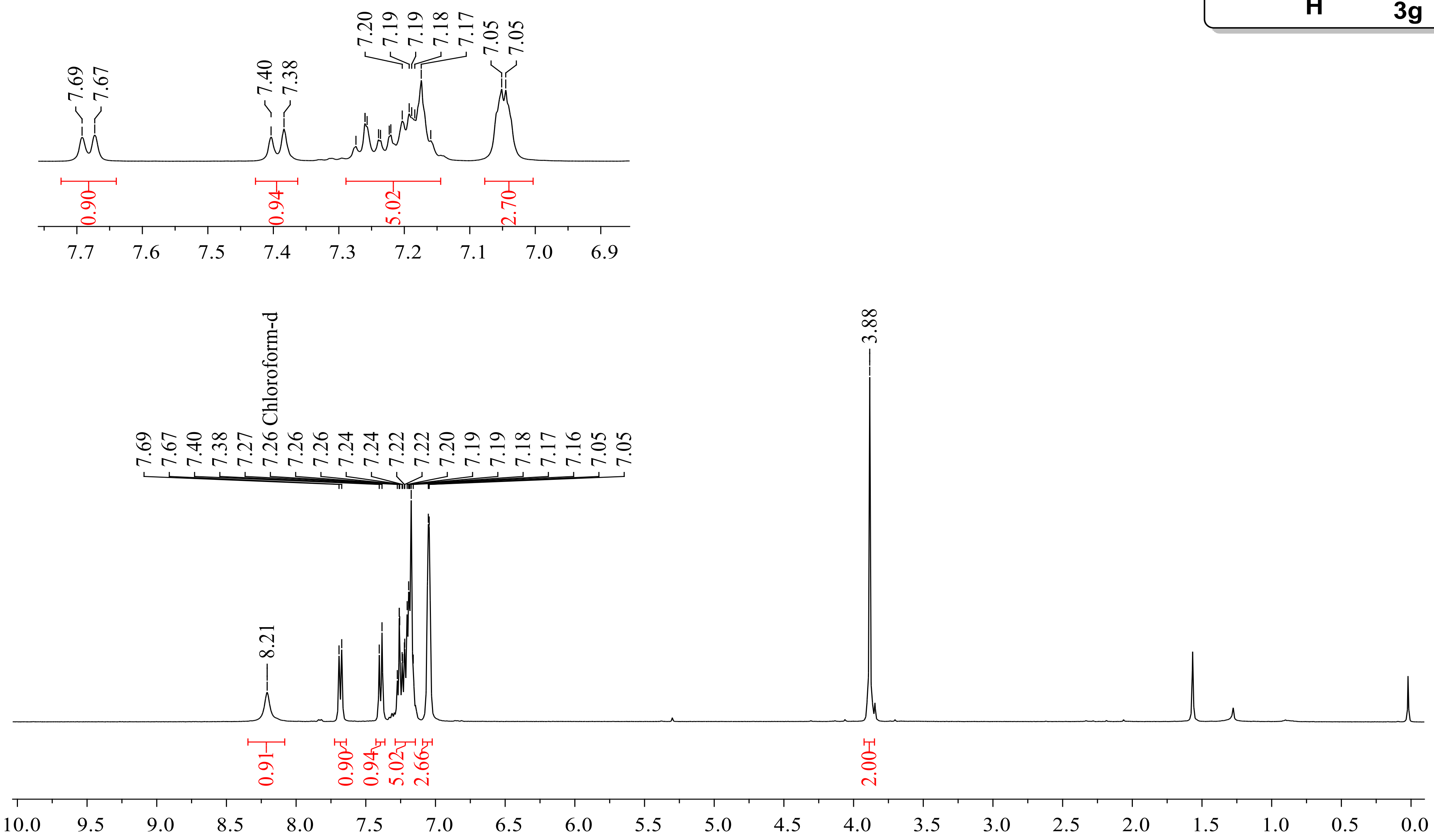

^{13}C NMR - 3-(benzylselanyl)-1H-indole (3g)

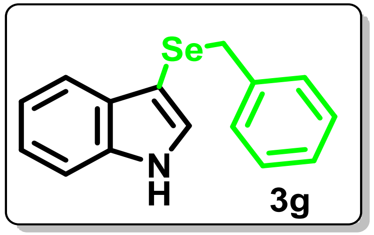

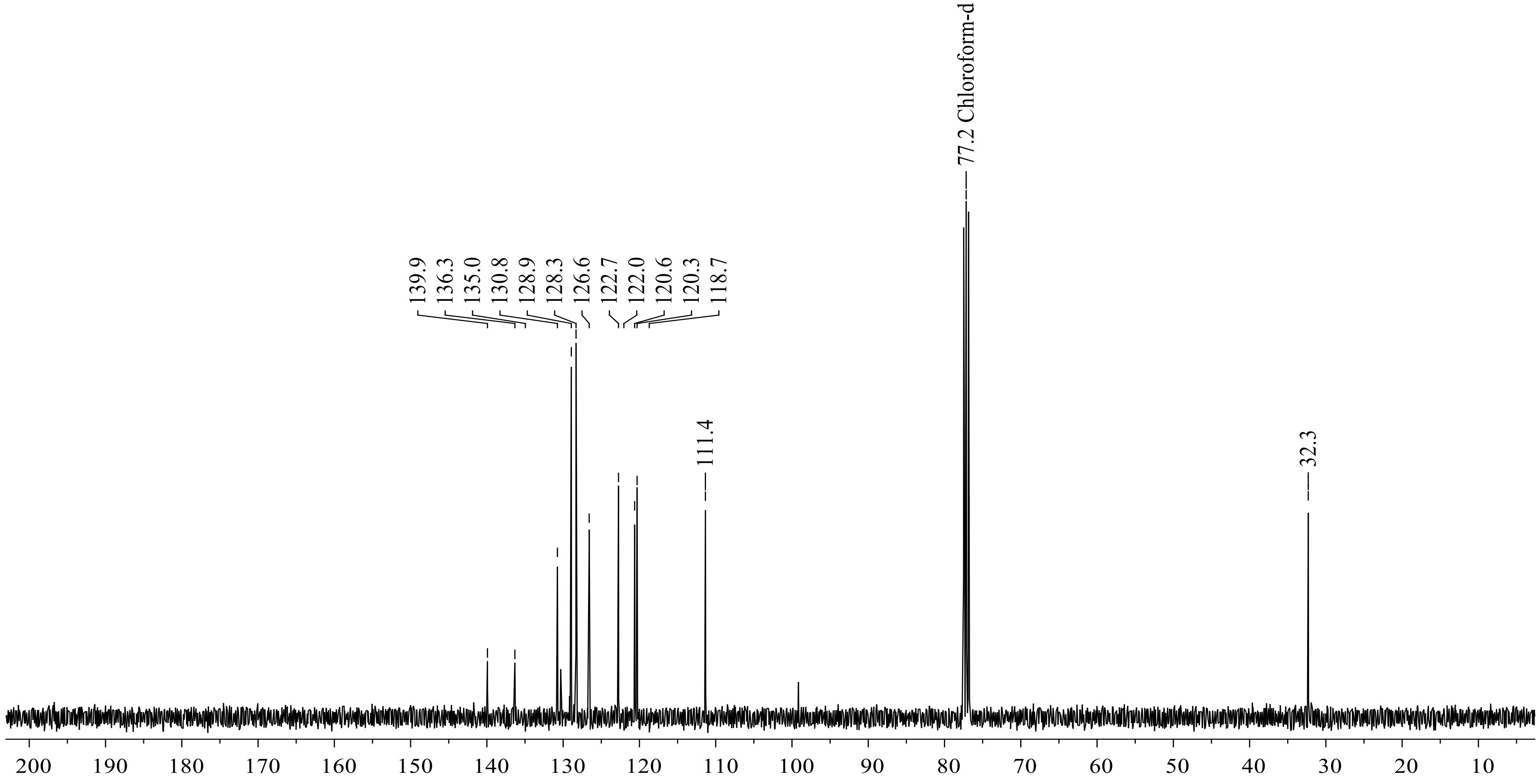

^{77}Se NMR - 3-(benzylselanyl)-1H-indole (3g)

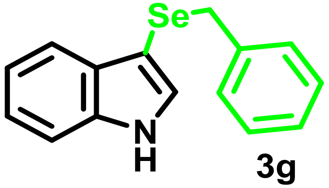

3g

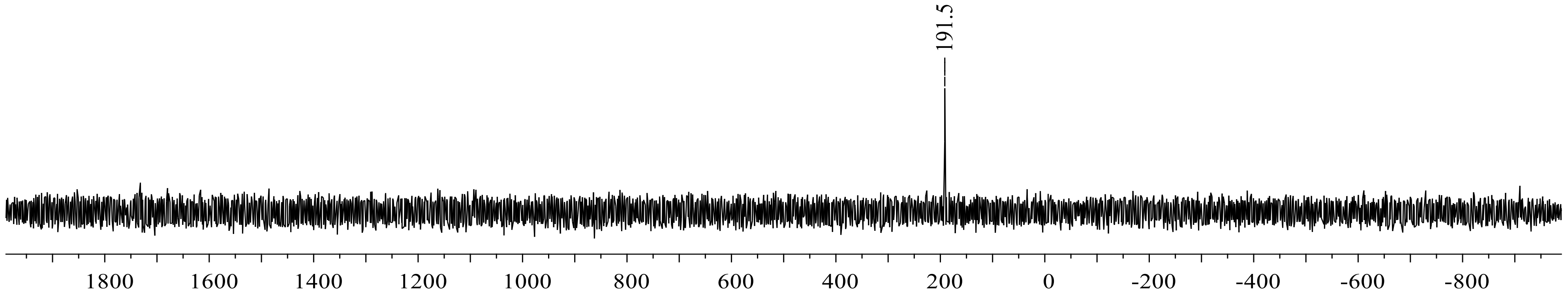

^{1}H NMR - 3-((4-methylbenzyl)selanyl)-1H-indole (3h)

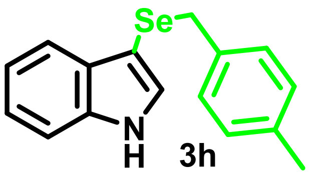

3h

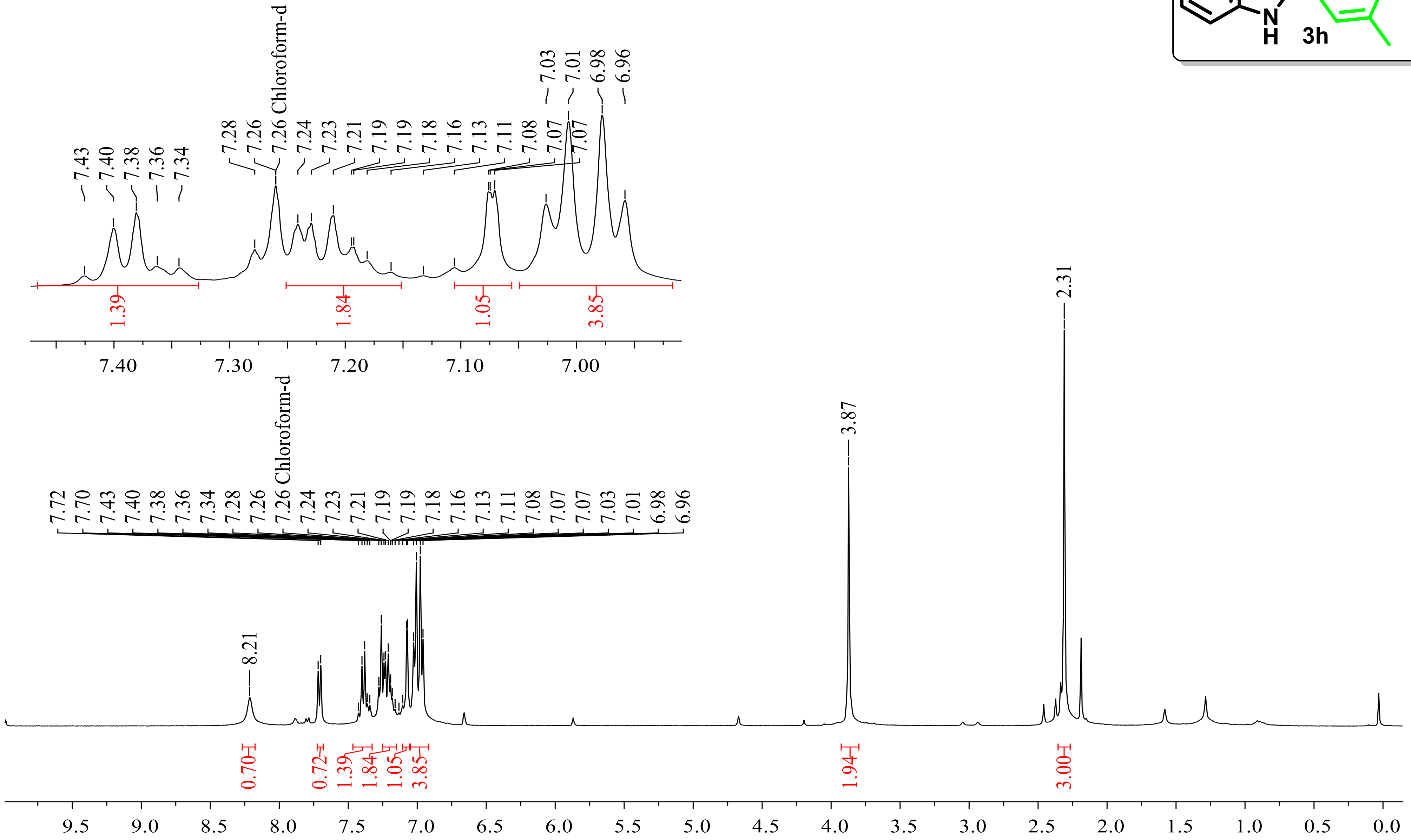

^{13}C NMR - 3-((4-methylbenzyl)selanyl)-1H-indole (3h)

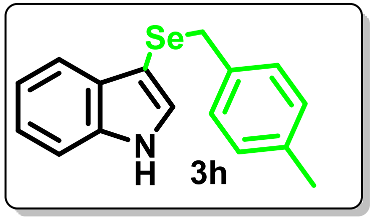

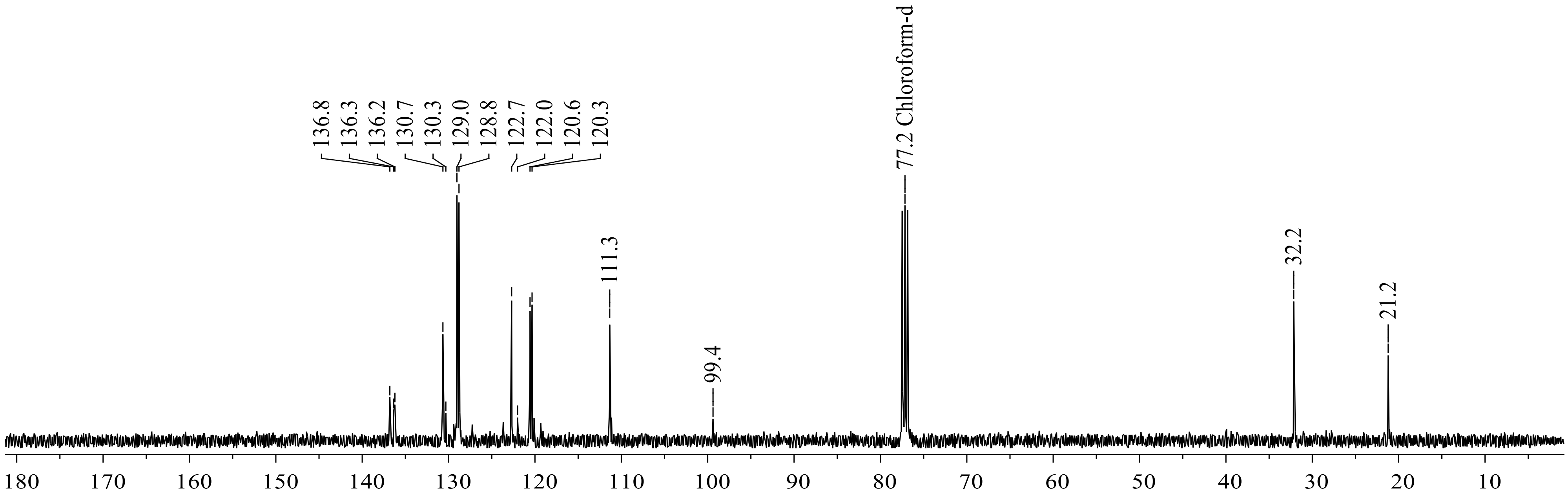

^{77}Se NMR - 3-((4-methylbenzyl)selanyl)-1H-indole (3h)

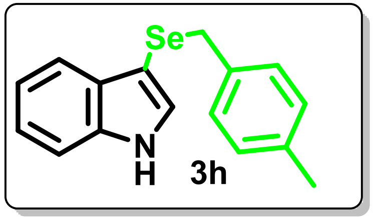

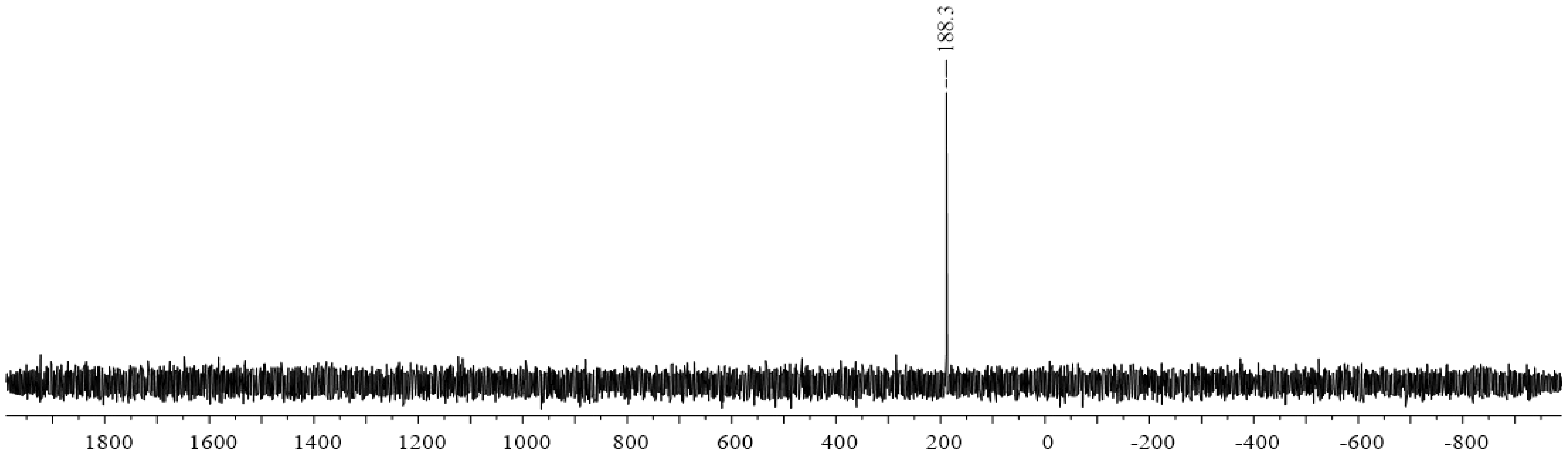

^{1}H NMR - 3-((4-(trifluoromethyl)benzyl)selanyl)-1H-indole (3i)

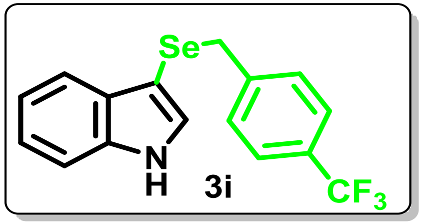

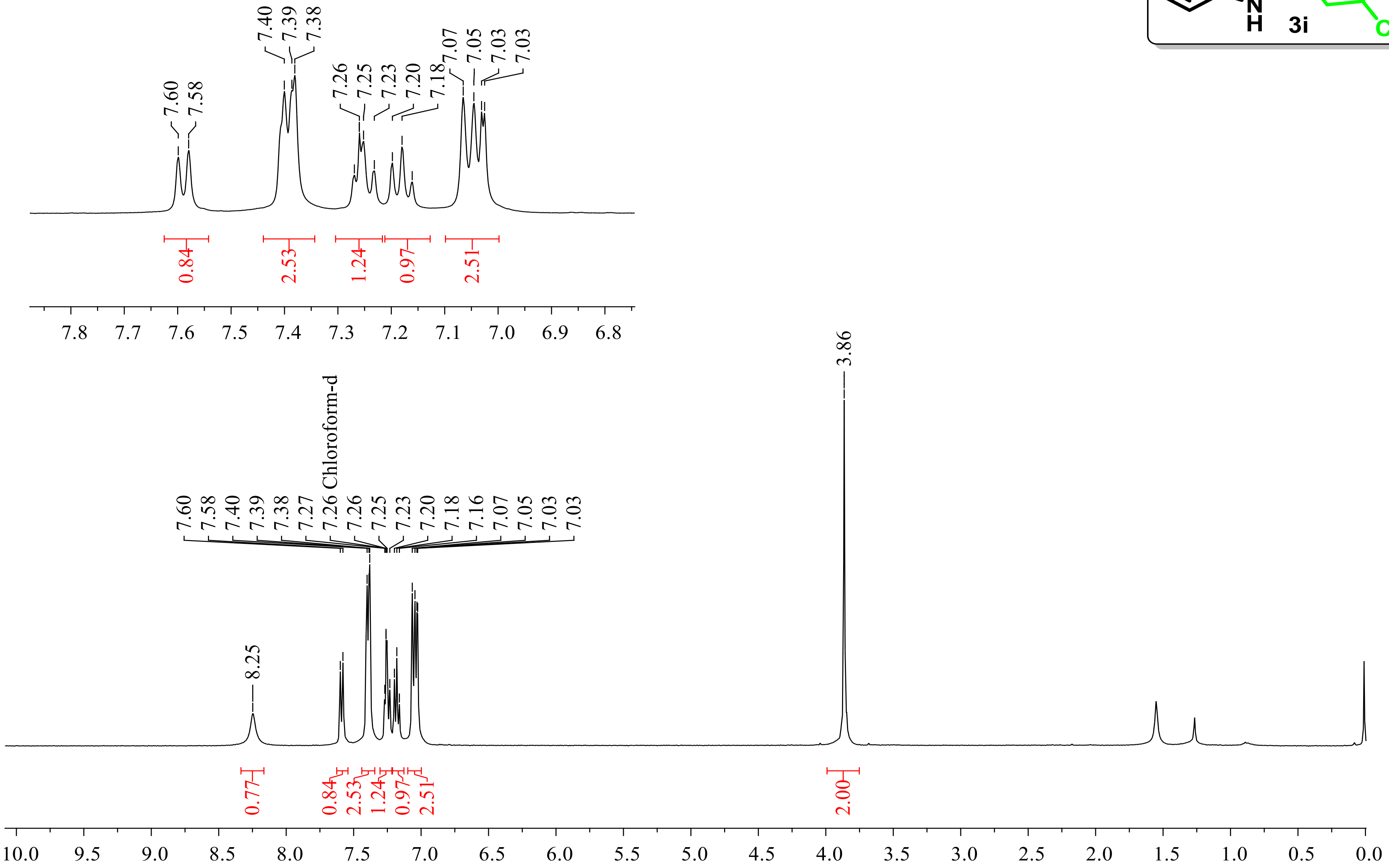

^{13}C NMR - 3-((4-(trifluoromethyl)benzyl)selanyl)-1H-indole (3i)

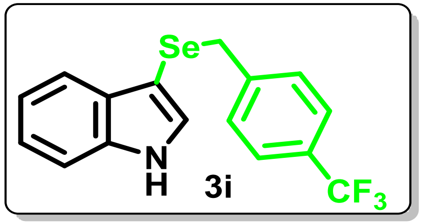

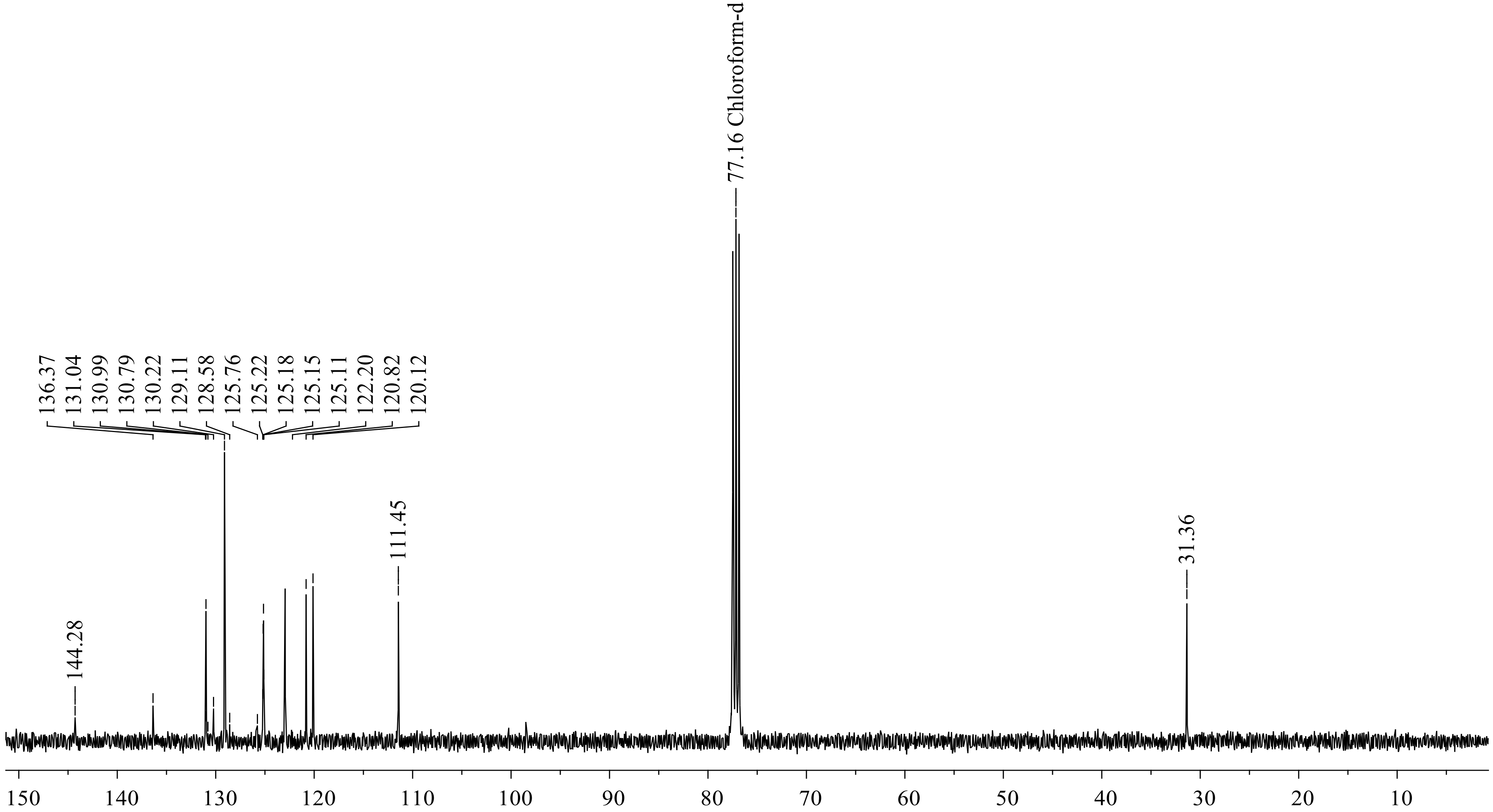

^{77}Se NMR - 3-((4-(trifluoromethyl)benzyl)selanyl)-1H-indole (3i)

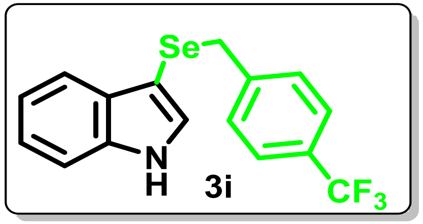

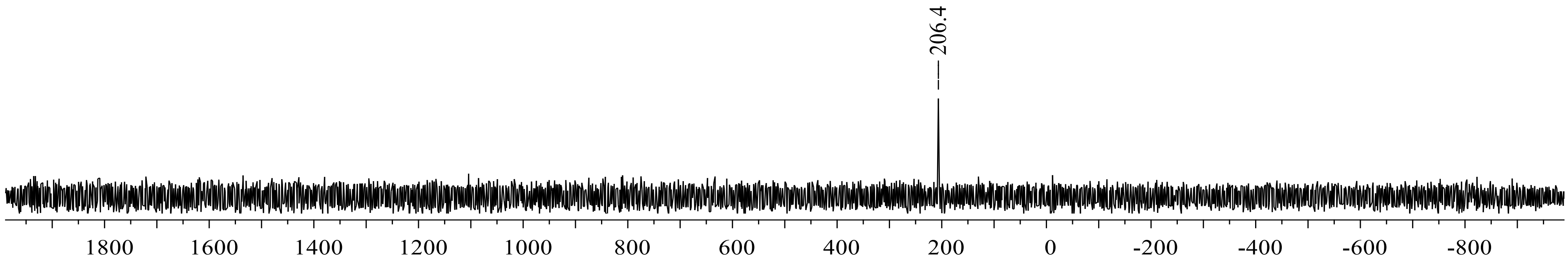

^{19}F NMR - 3-((4-(trifluoromethyl)benzyl)selanyl)-1H-indole (3i)

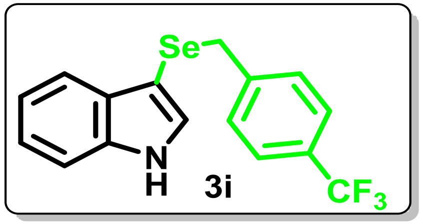

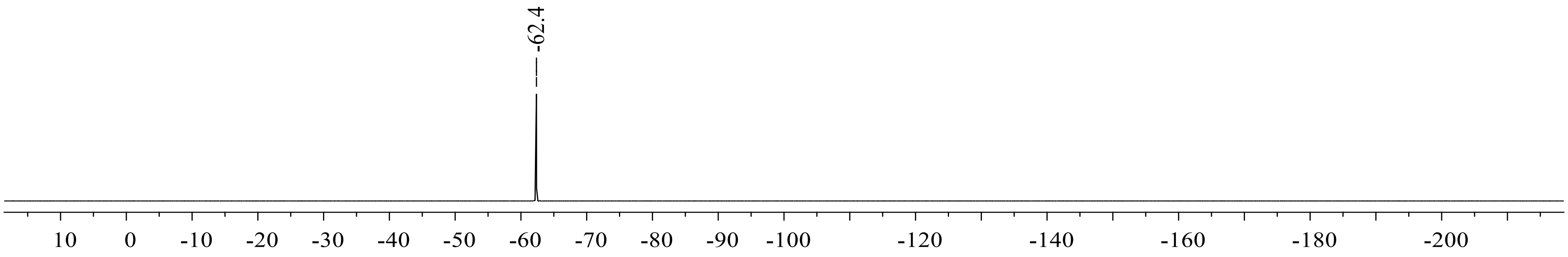

^{1}H NMR - 3-((4-fluorobenzyl)selanyl)-1H-indole (3j)

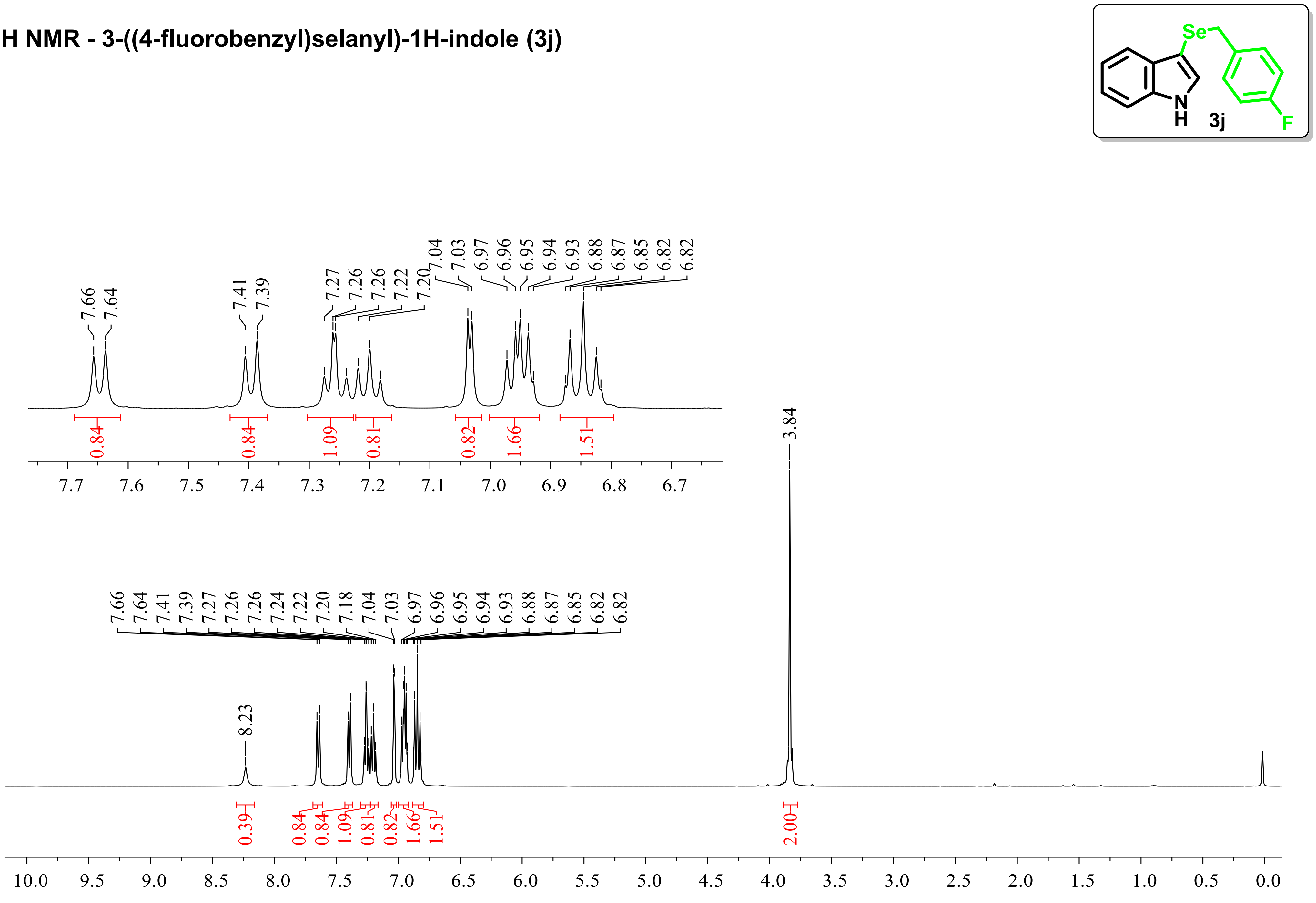

^{13}C NMR - 3-((4-fluorobenzyl)selanyl)-1H-indole (3j)

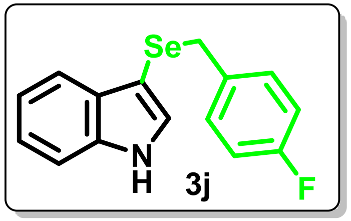

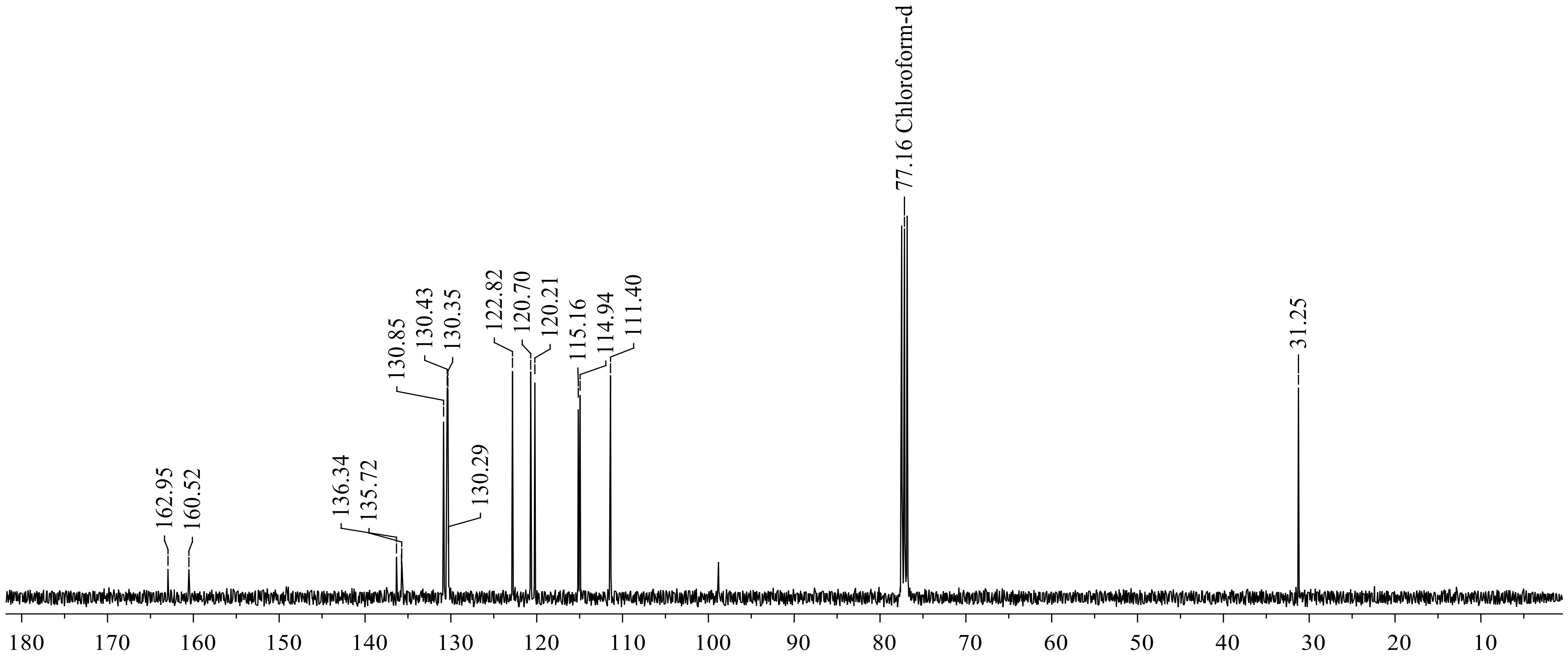

^{77}Se NMR - 3-((4-fluorobenzyl)selanyl)-1H-indole (3j)

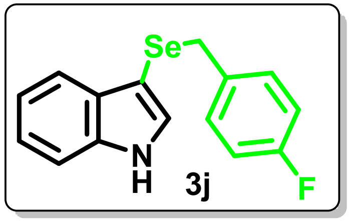

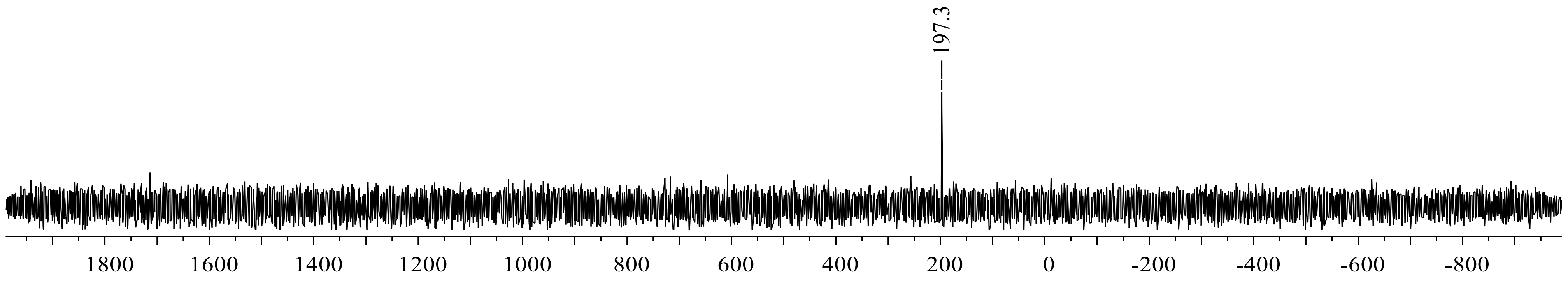

^{19}F NMR - 3-((4-fluorobenzyl)selanyl)-1H-indole (3j)

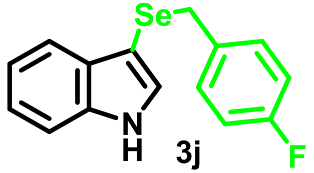

3j

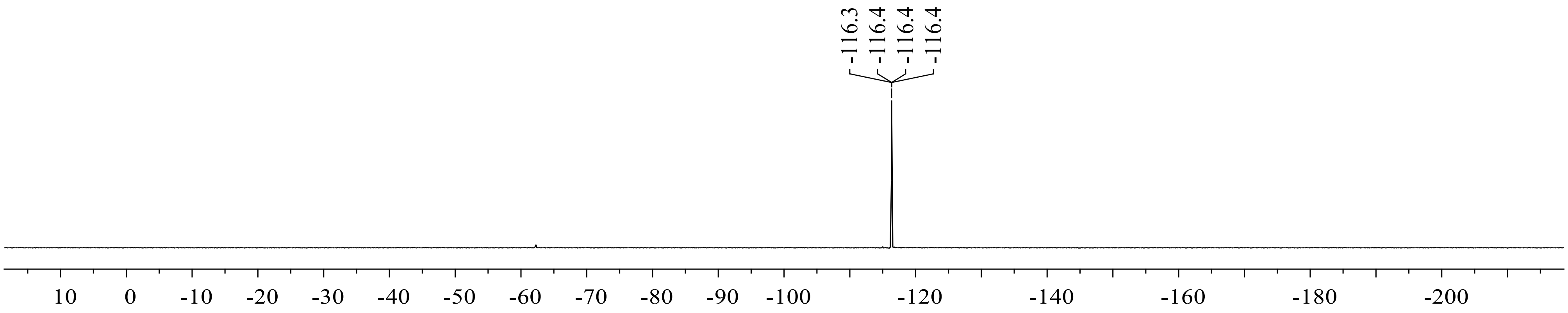

^{1}H NMR - 2-methyl-3-(phenylselanyl)-1H-indole (3k).

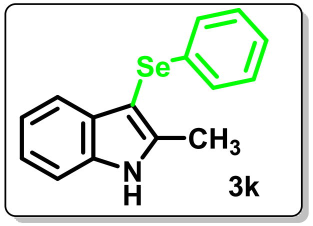

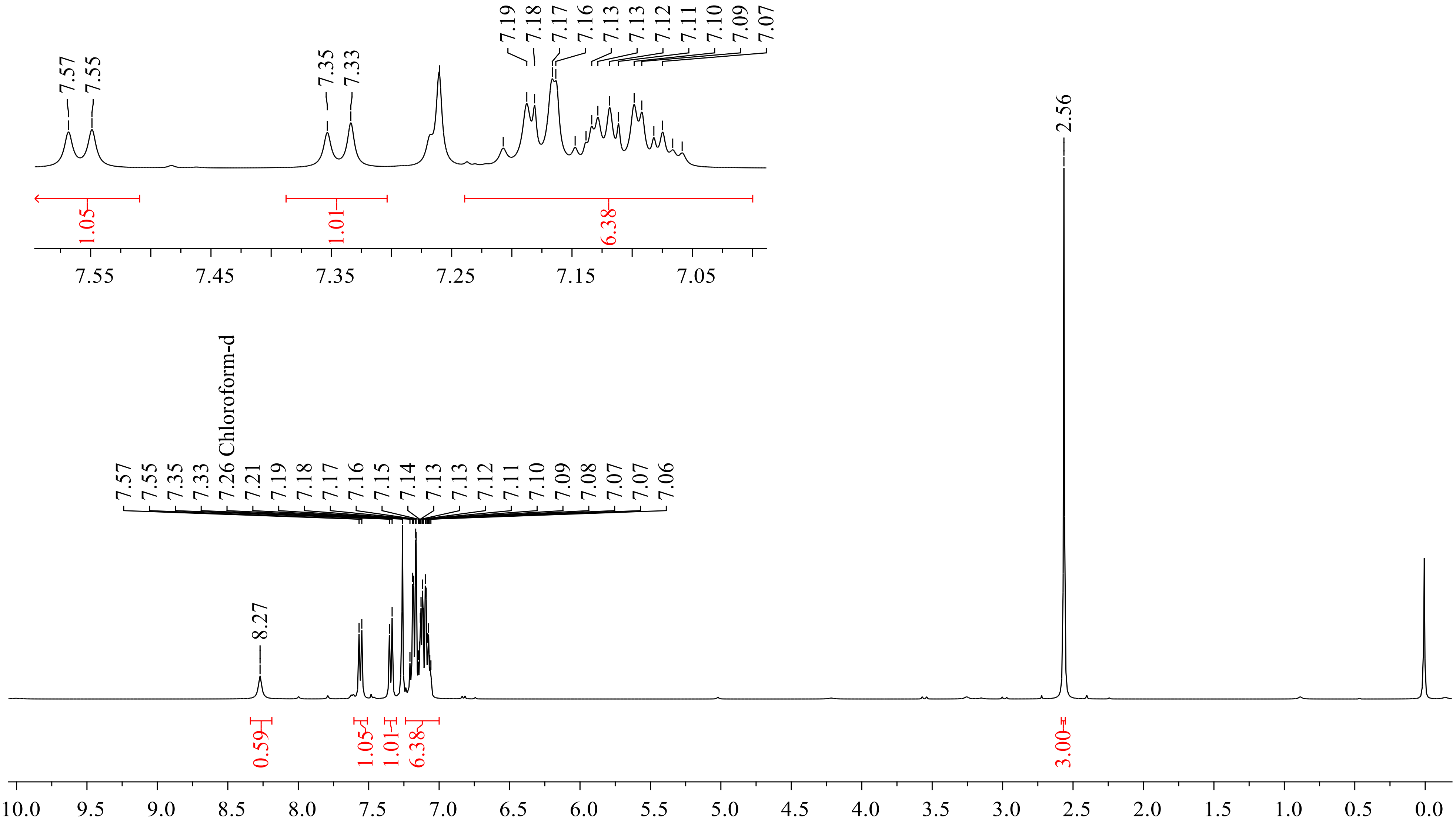

^{13}C NMR - 2-methyl-3-(phenylselanyl)-1H-indole (3k).

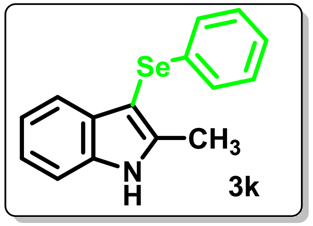

3k

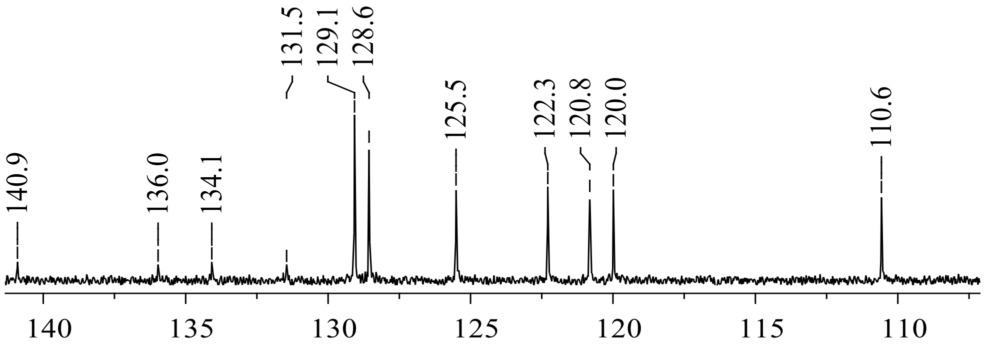

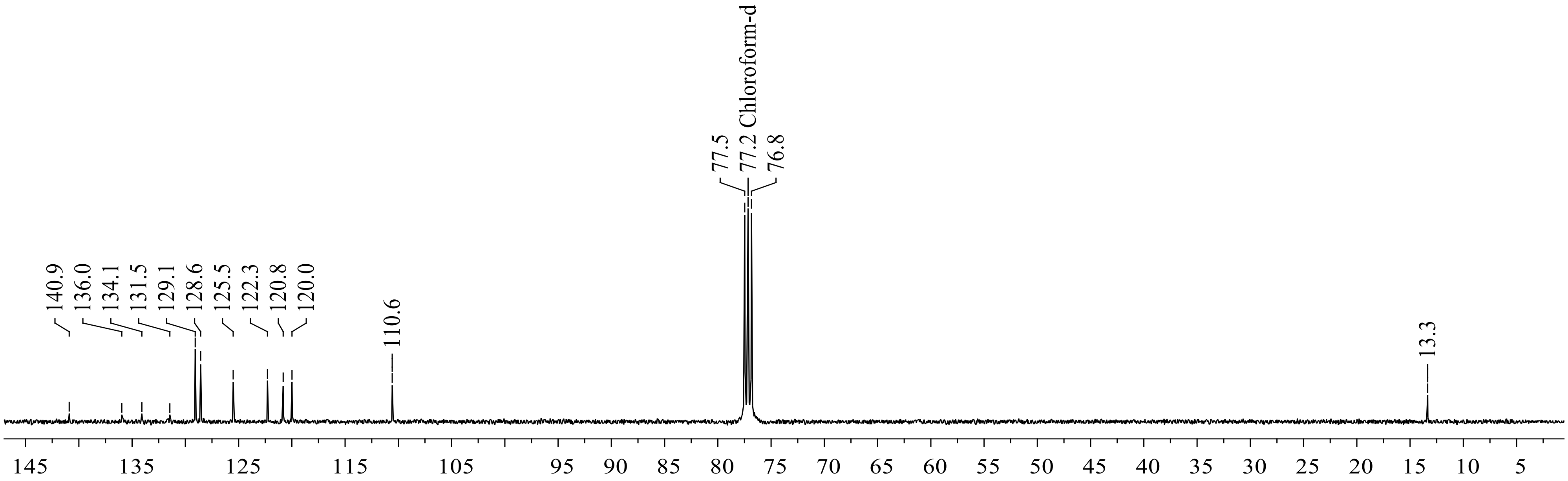

^{77}Se NMR - 2-methyl-3-(phenylselanyl)-1H-indole (3k).

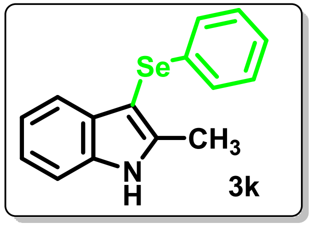

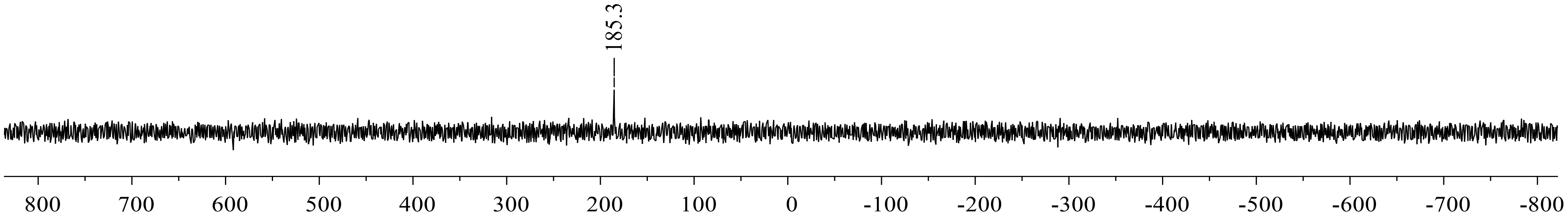

^{1}H NMR - 1-methyl-3-(phenylselanyl)-1H-indole (3l).

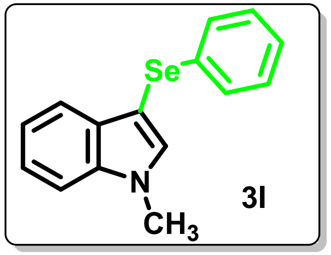

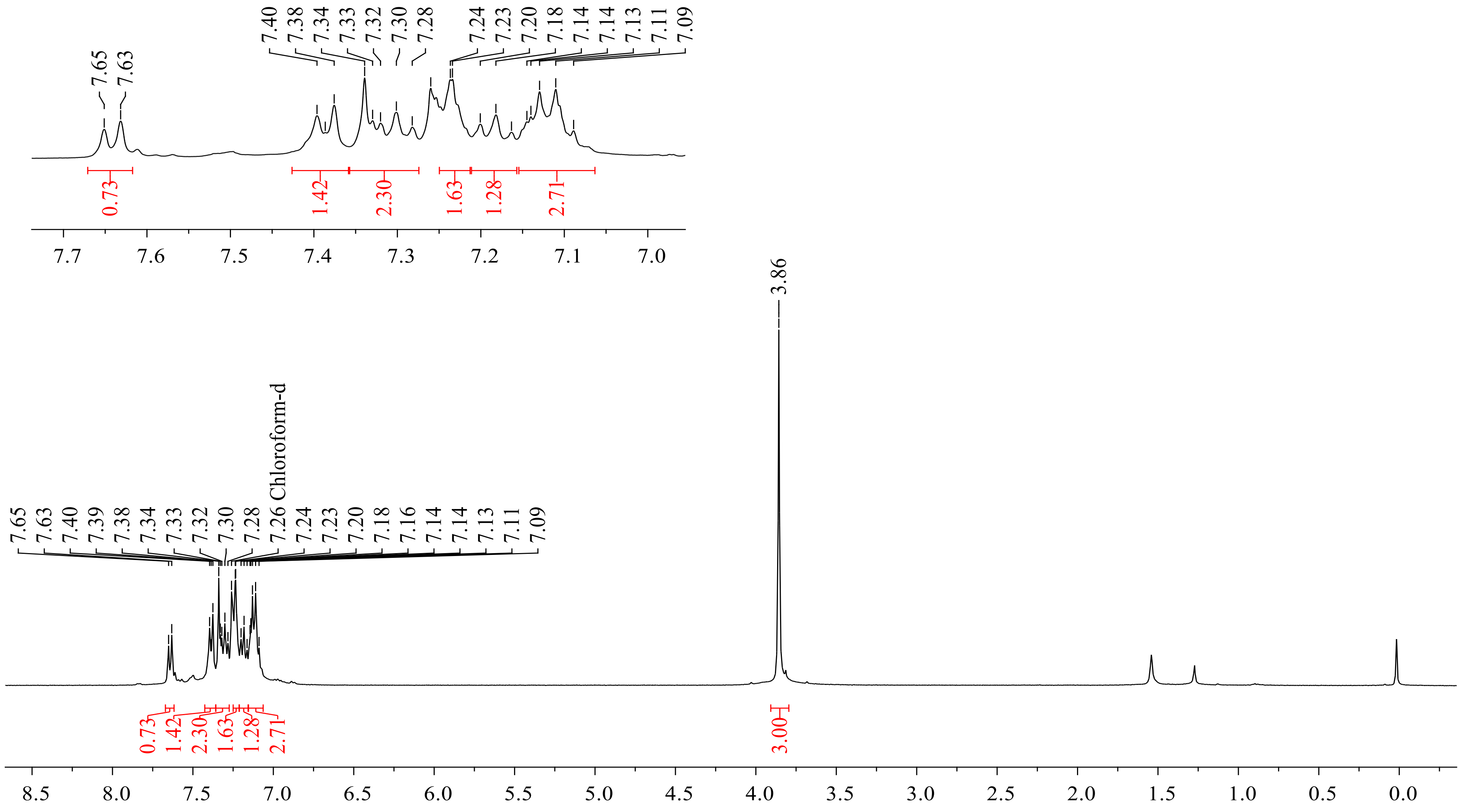

^{13}C NMR - 1-methyl-3-(phenylselanyl)-1H-indole (3l).

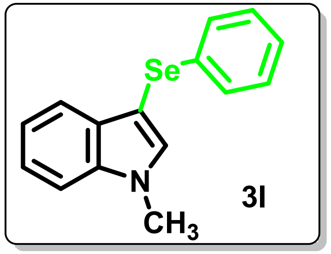

3l

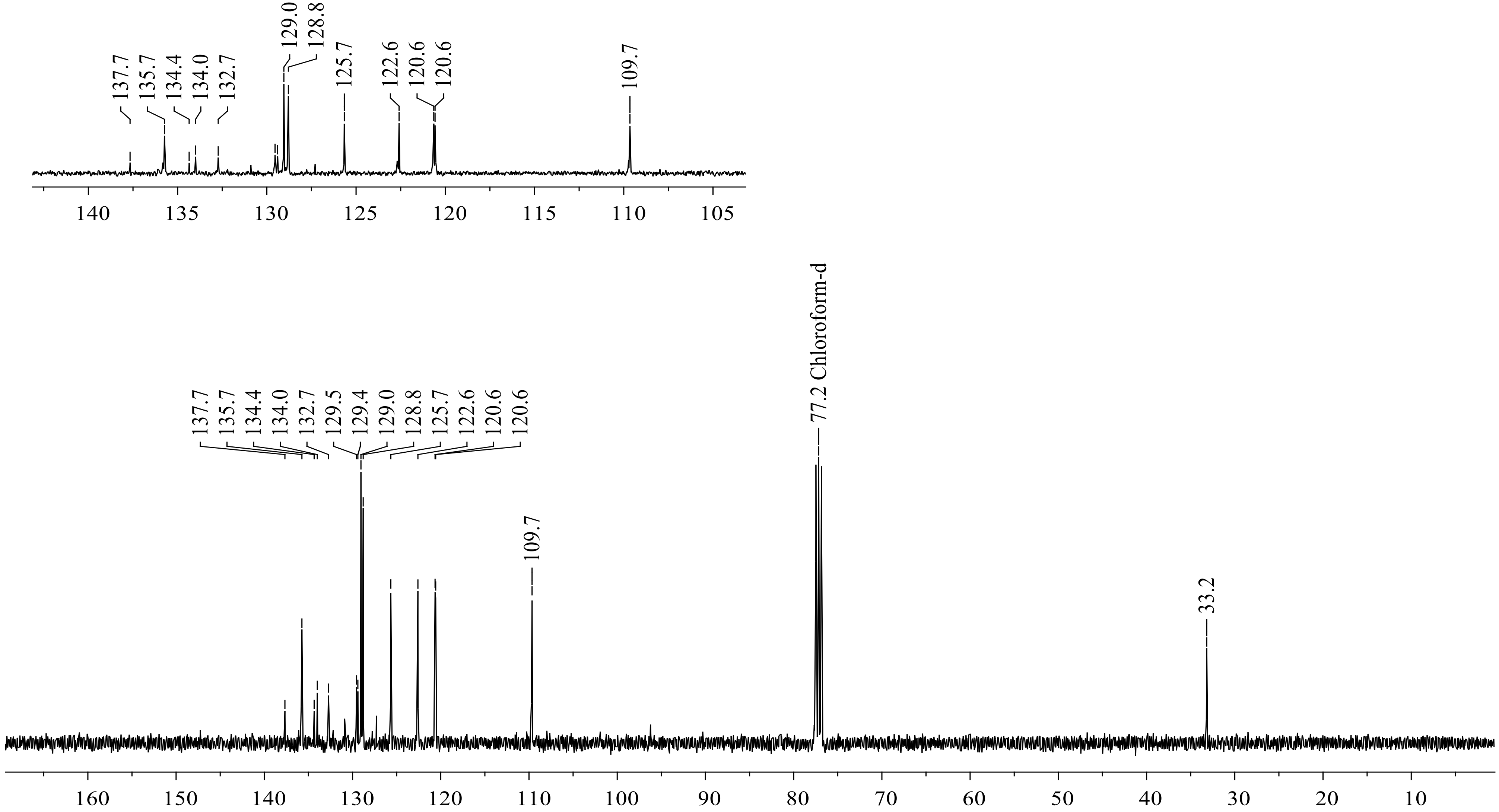

^{77}Se NMR - 1-methyl-3-(phenylselanyl)-1H-indole (3l).

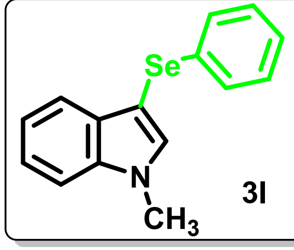

3l

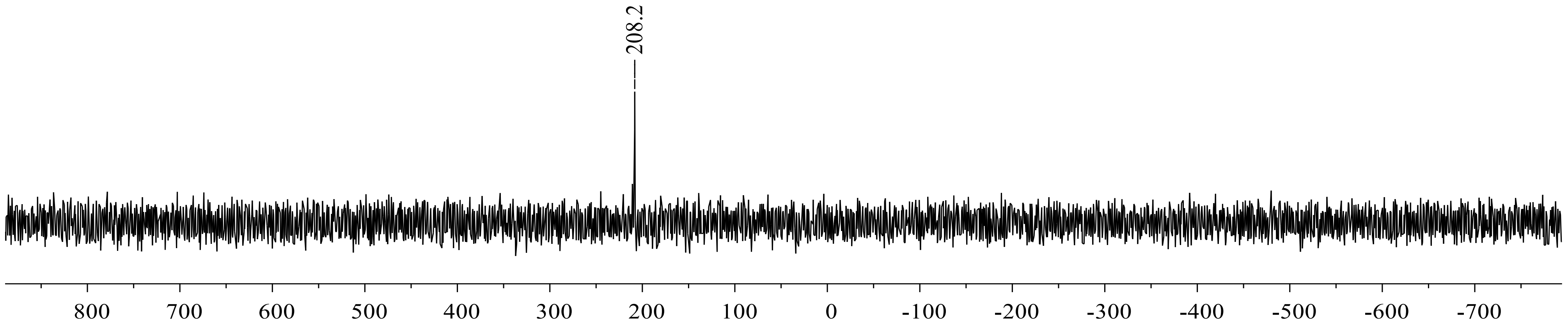

^{1}H NMR - 1-(but-3-en-1-yl)-3-(phenylselanyl)-1H-indole (3m).

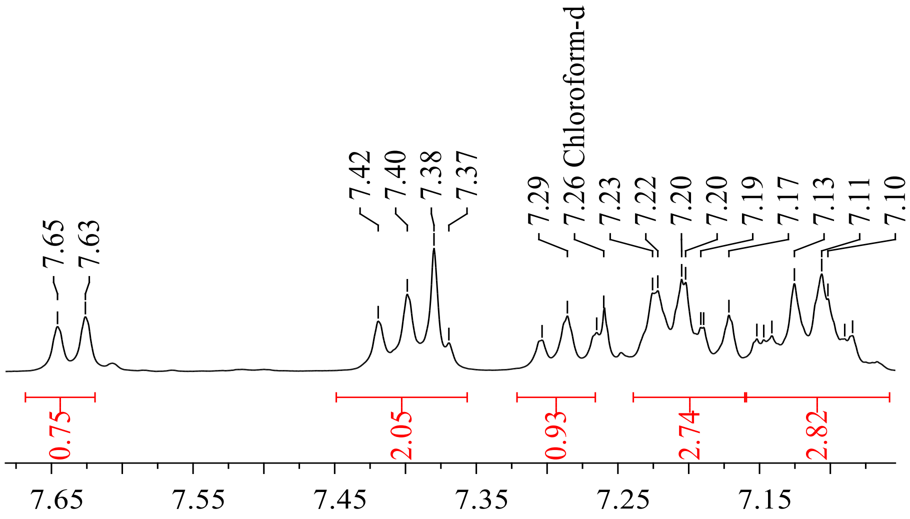

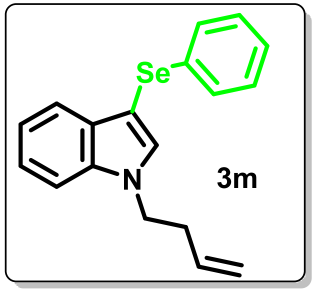

3m

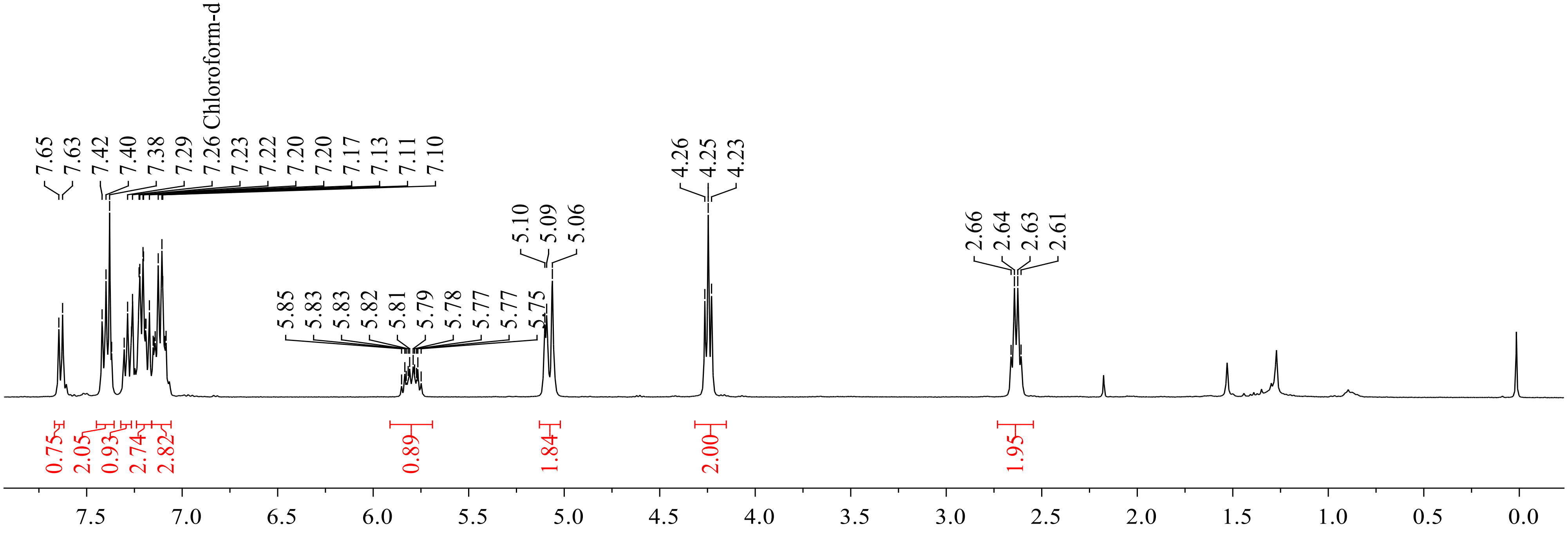

^{13}C NMR - 1-(but-3-en-1-yl)-3-(phenylselanyl)-1H-indole (3m).

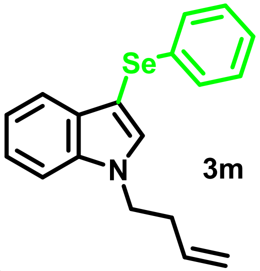

3m

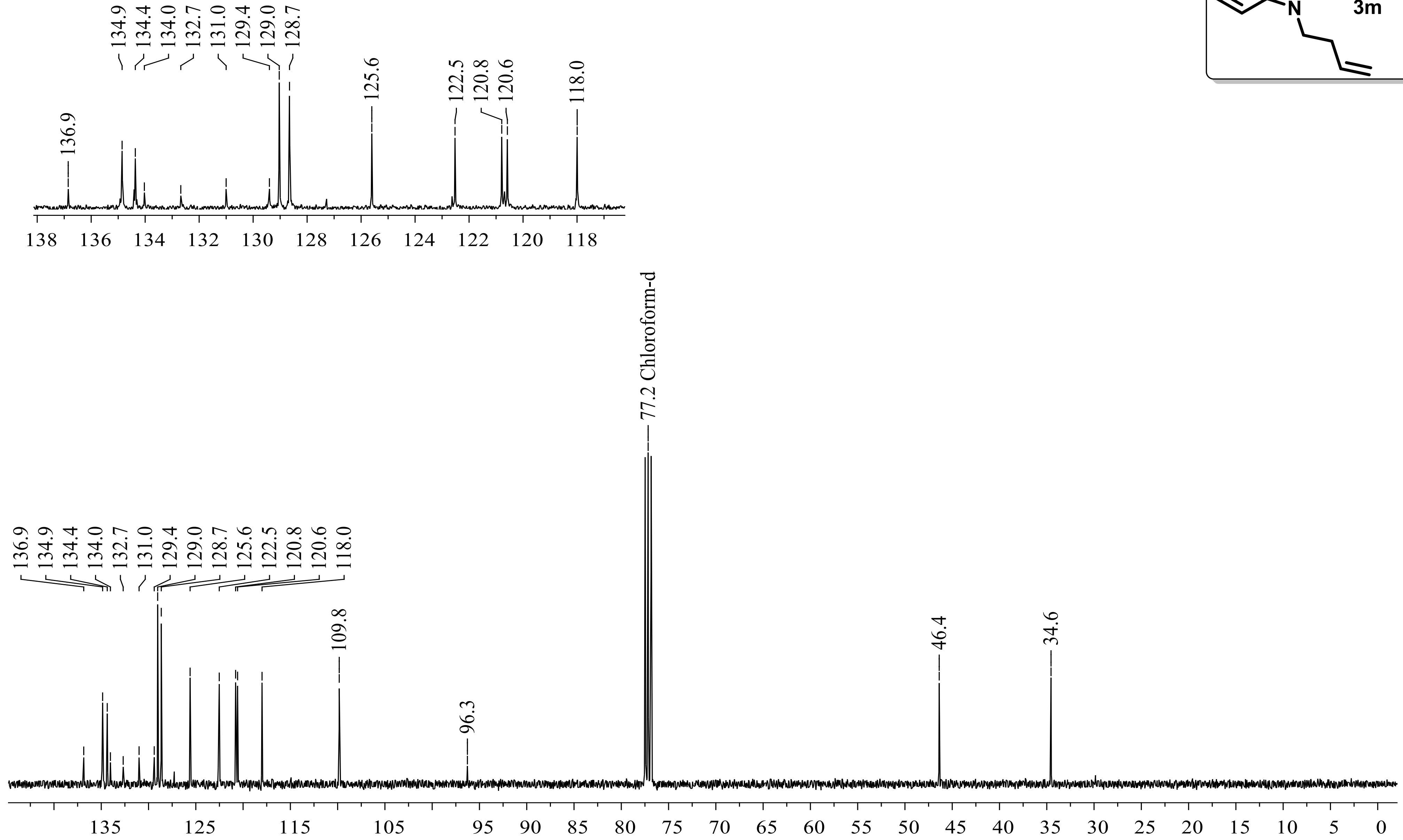

^{77}Se NMR - 1-(but-3-en-1-yl)-3-(phenylselanyl)-1H-indole (3m).

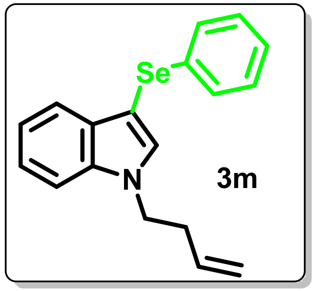

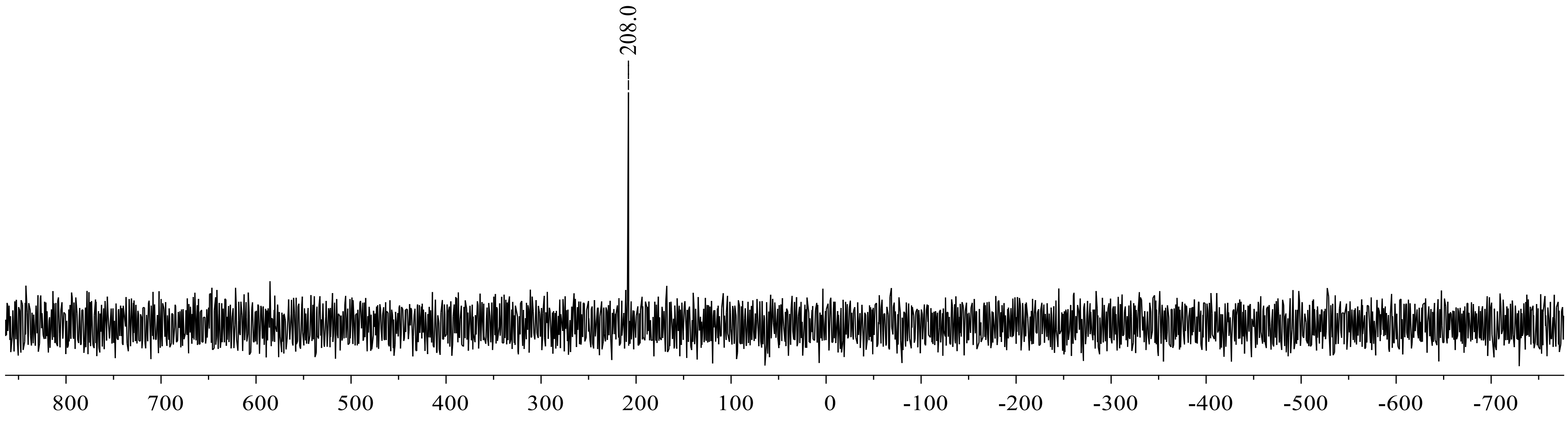

^{1}H NMR - *N,N*-dimethyl-4-(phenylselanyl)aniline (5a)

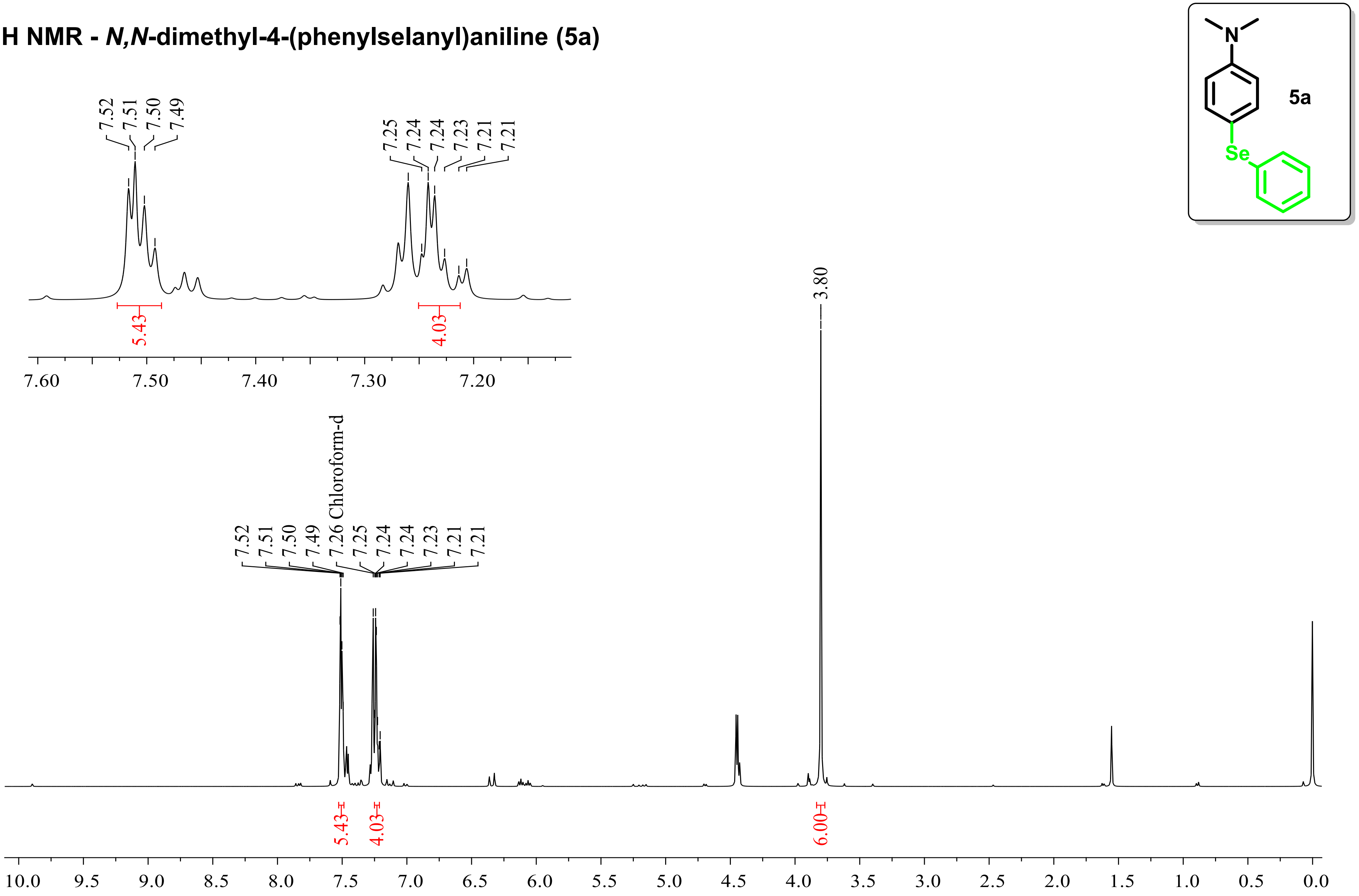

^{13}C NMR - *N,N*-dimethyl-4-(phenylselanyl)aniline (5a)

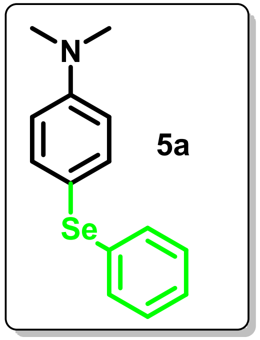

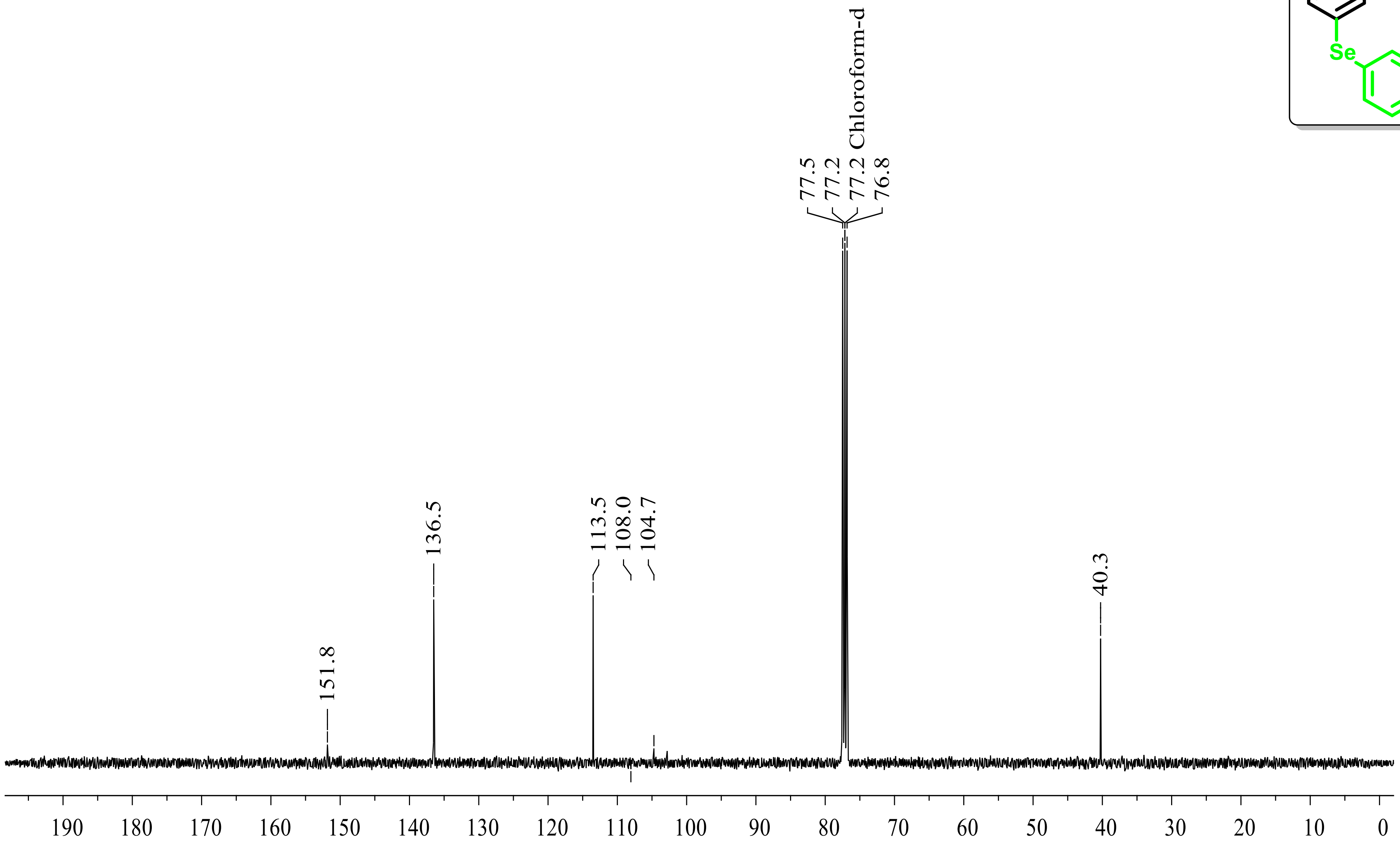

^{77}Se NMR - *N,N*-dimethyl-4-(phenylselanyl)aniline (5a)

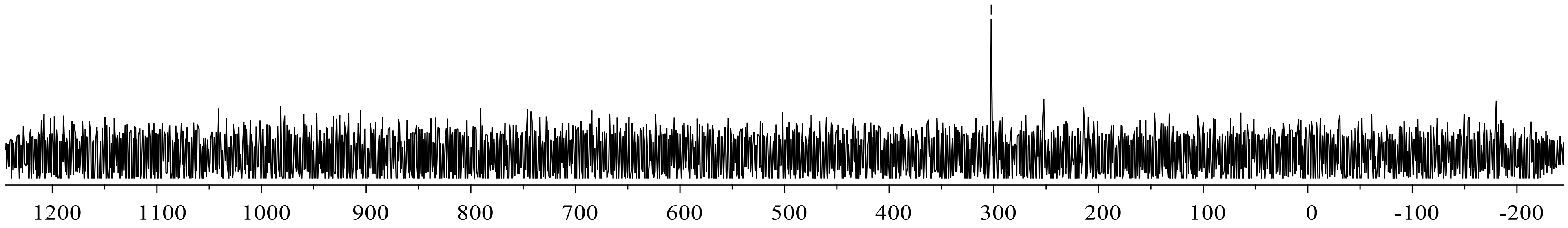

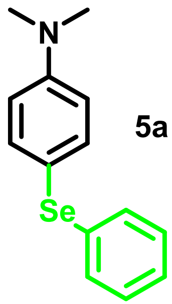

5a

^{1}H NMR - 2-(phenylselanyl)benzene-1,3,5-triol (5b).

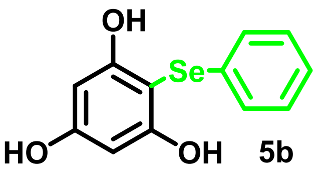

5b

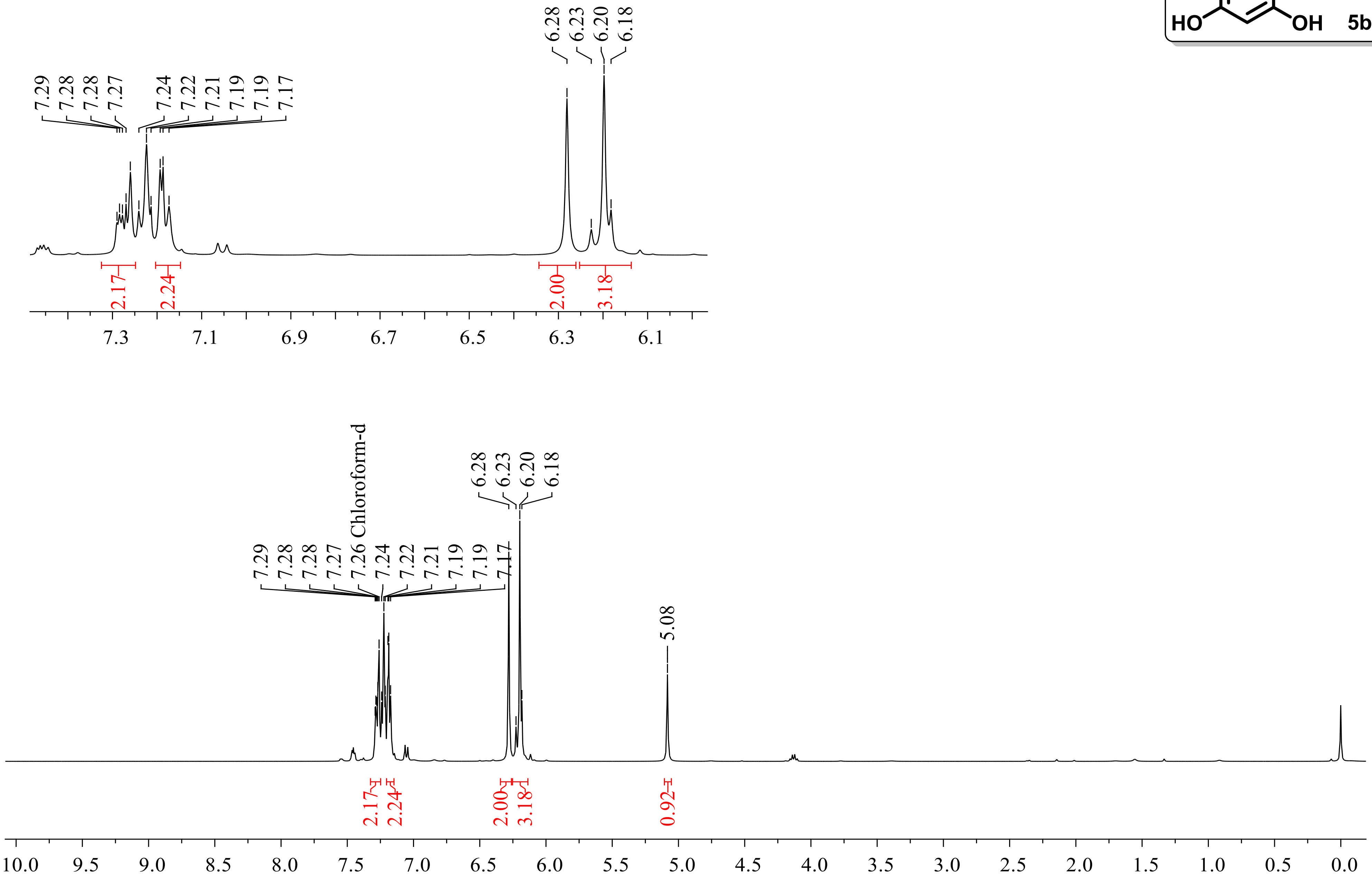

^{13}C NMR - 2-(phenylselanyl)benzene-1,3,5-triol (5b).

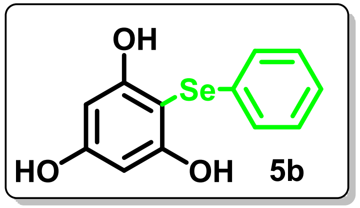

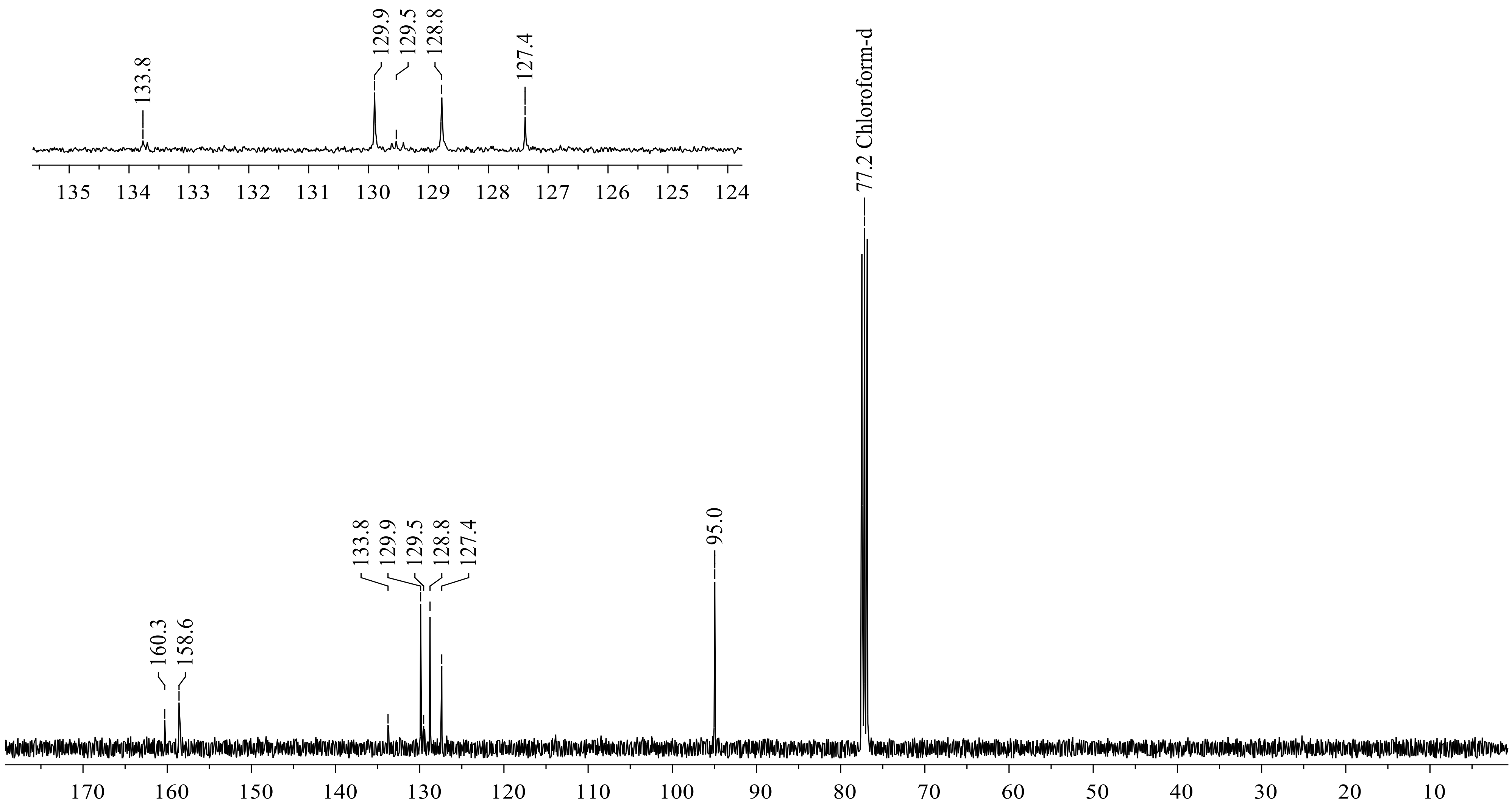

^{77}Se NMR - 2-(phenylselanyl)benzene-1,3,5-triol (5b).

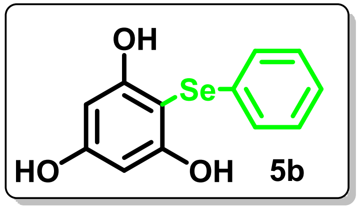

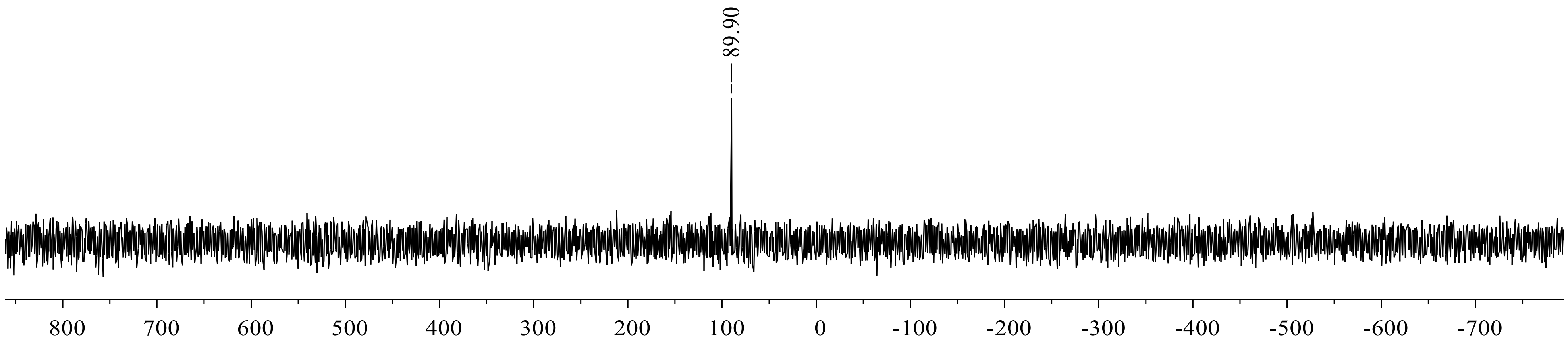

^{1}H NMR - 2,4-bis(phenylselanyl)benzene-1,3,5-triol (5c).

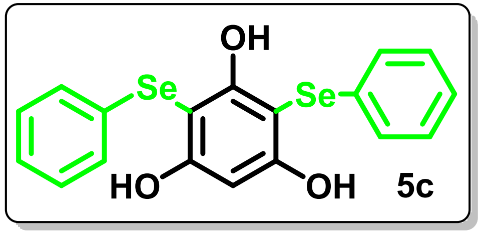

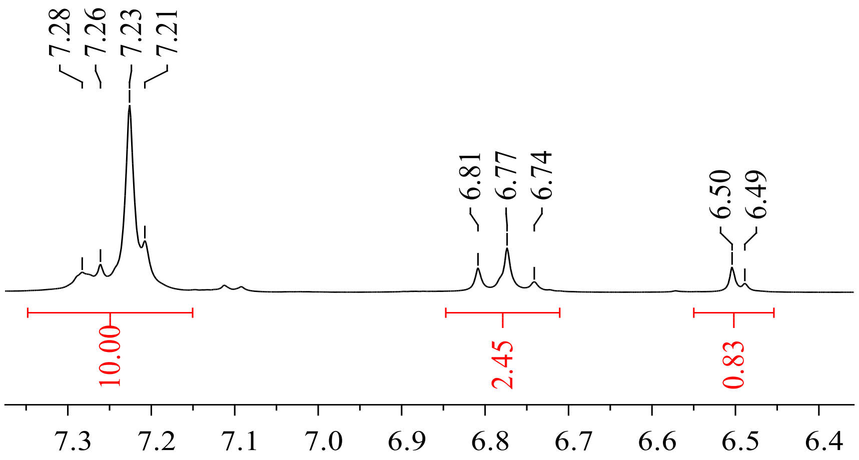

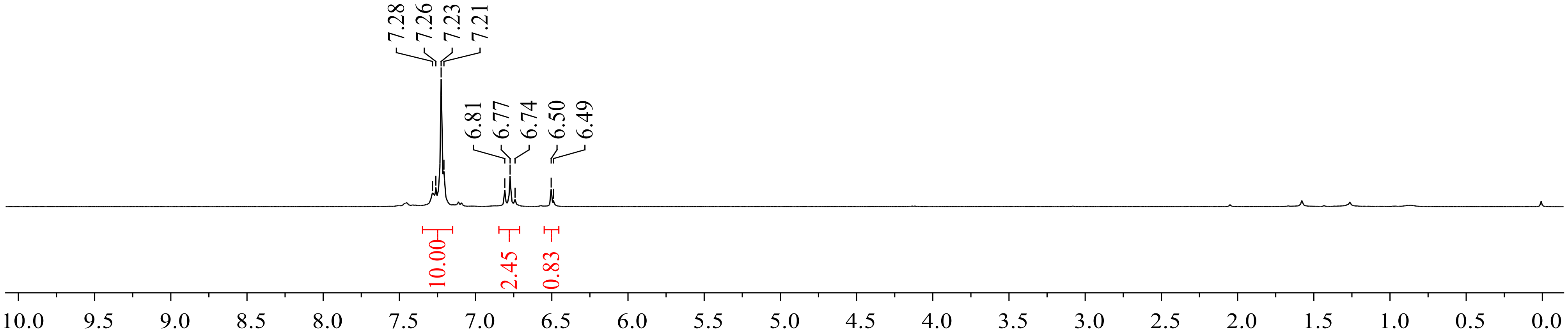

^{13}C NMR - 2,4-bis(phenylselanyl)benzene-1,3,5-triol (5c).

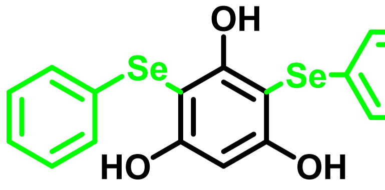

5c

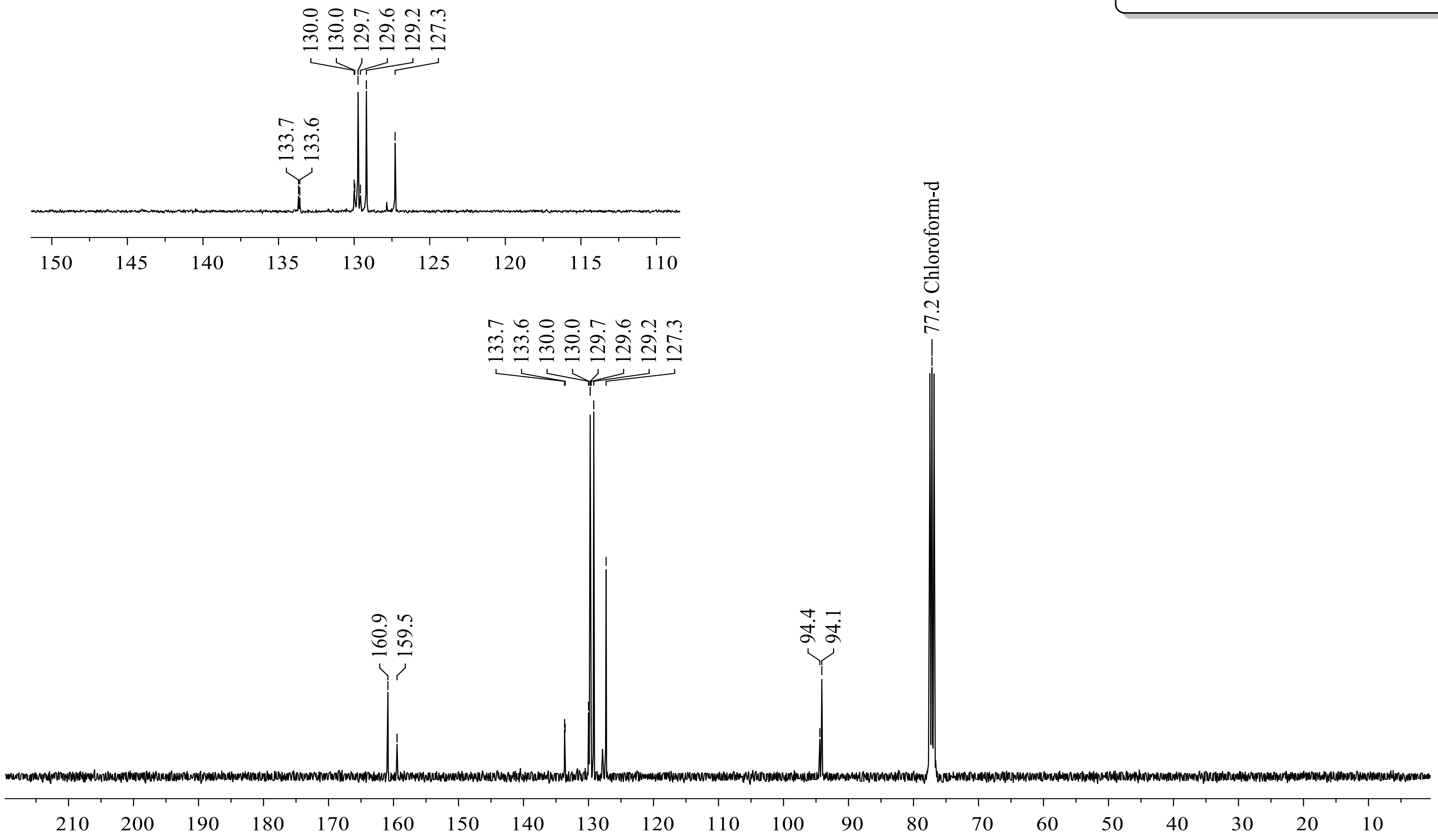

^{77}Se NMR - 2,4-bis(phenylselanyl)benzene-1,3,5-triol (5c).

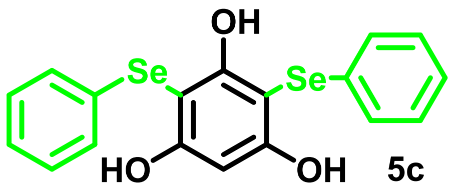

5c

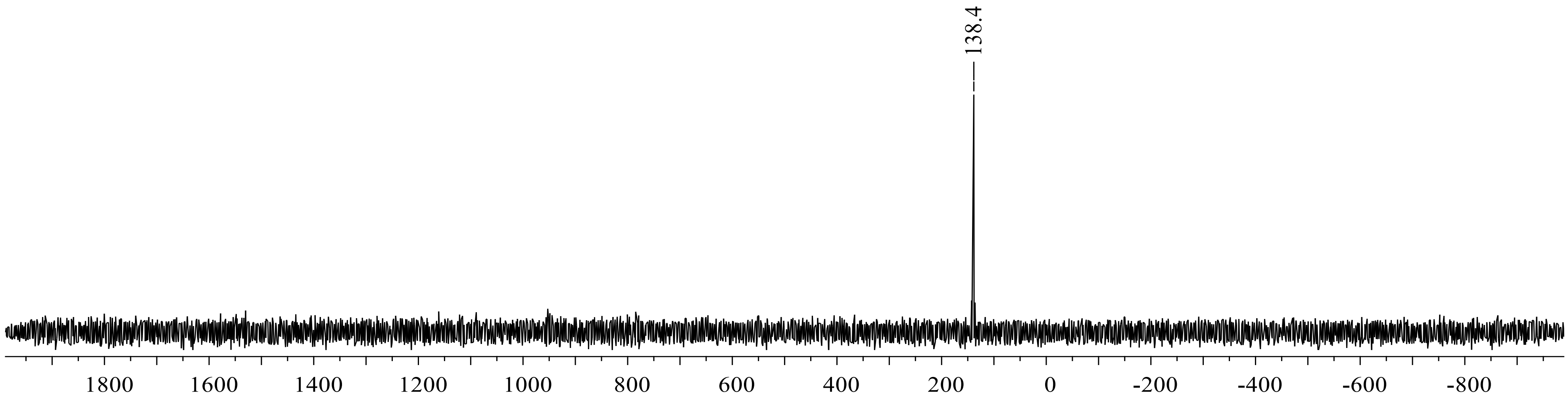

^{1}H NMR - 4-phenyl-5-(phenylselanyl)thiazol-2-amine (5d).

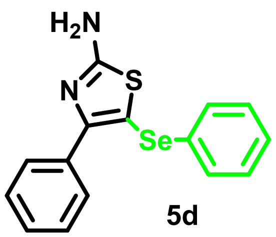

5d

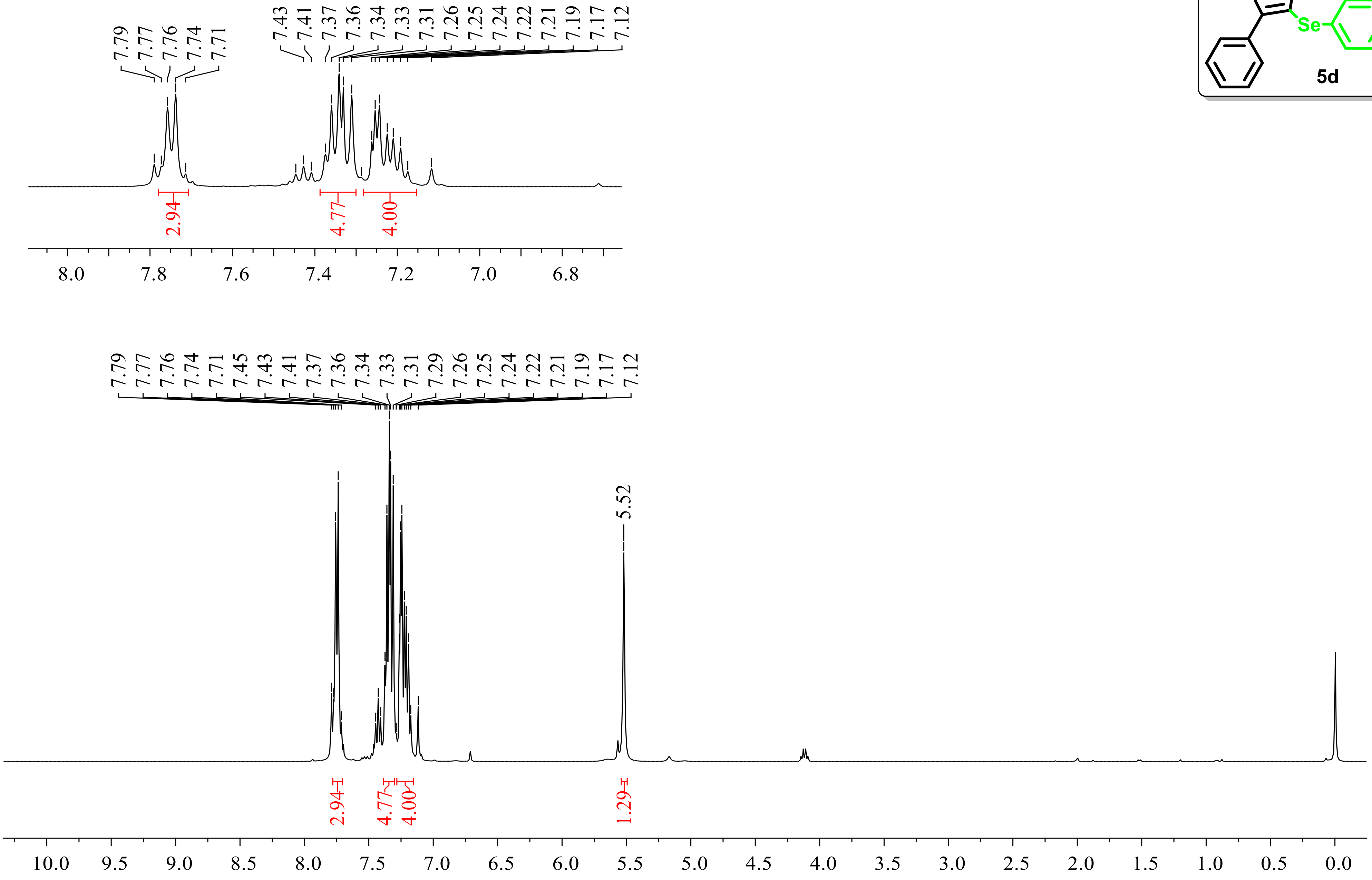

^{13}C NMR - 4-phenyl-5-(phenylselanyl)thiazol-2-amine (5d).

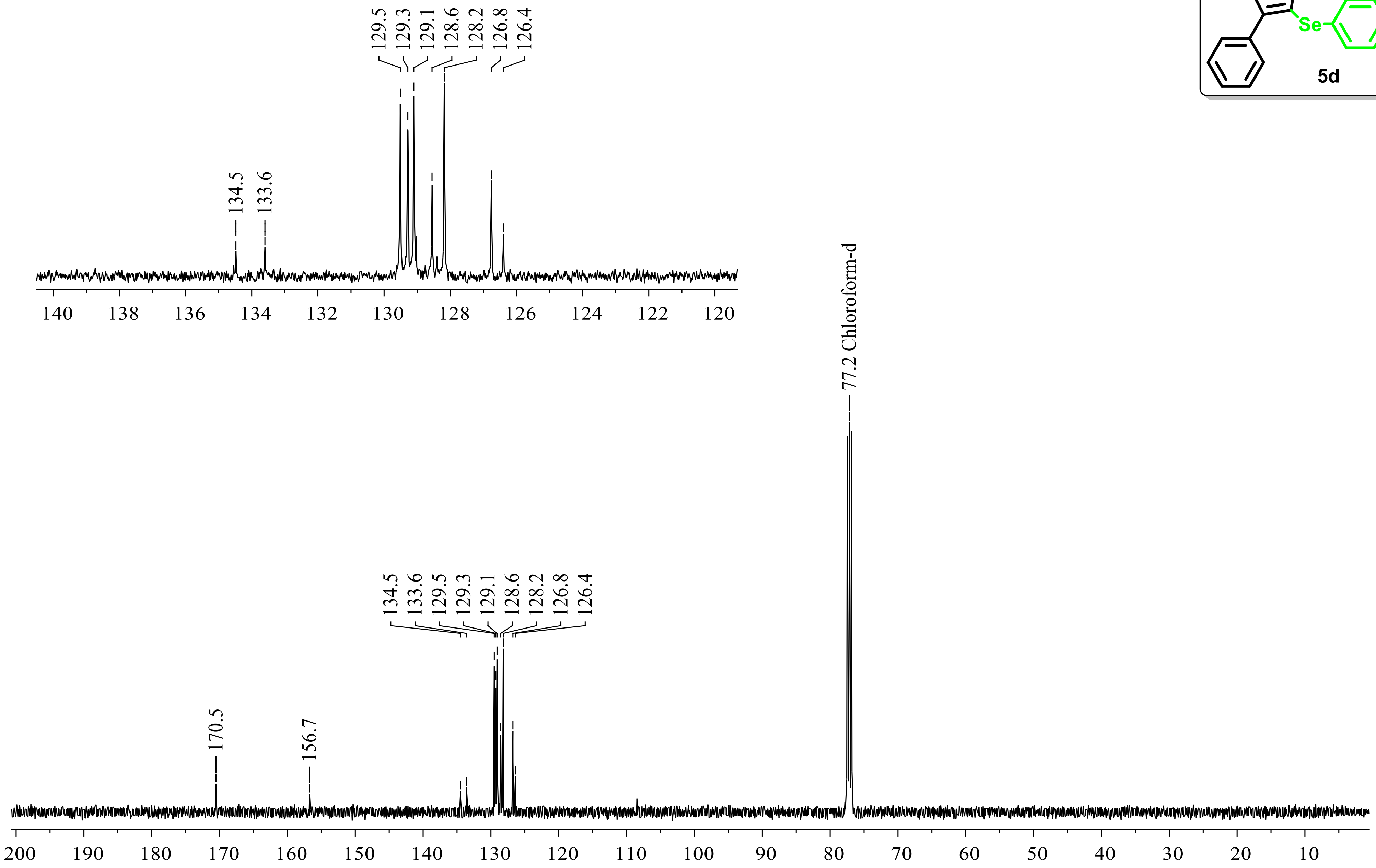

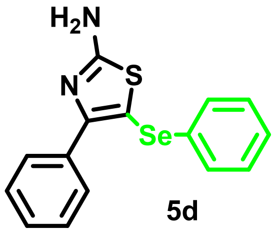

5d

^{77}Se NMR - 4-phenyl-5-(phenylselanyl)thiazol-2-amine (5d).

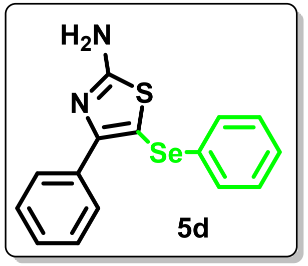

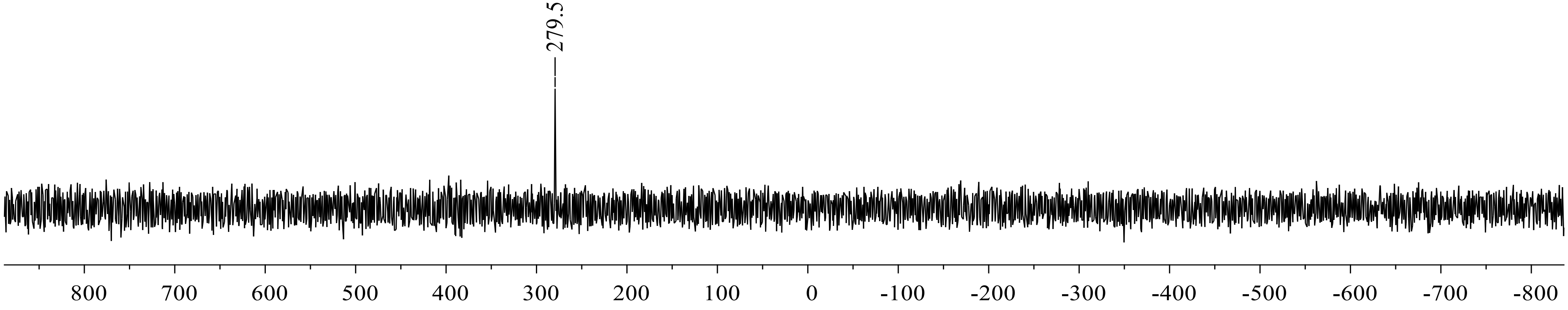

^{1}H NMR - (1-ethoxy-1-(4-methoxyphenyl)propan-2-yl)(phenyl)selane (5e).

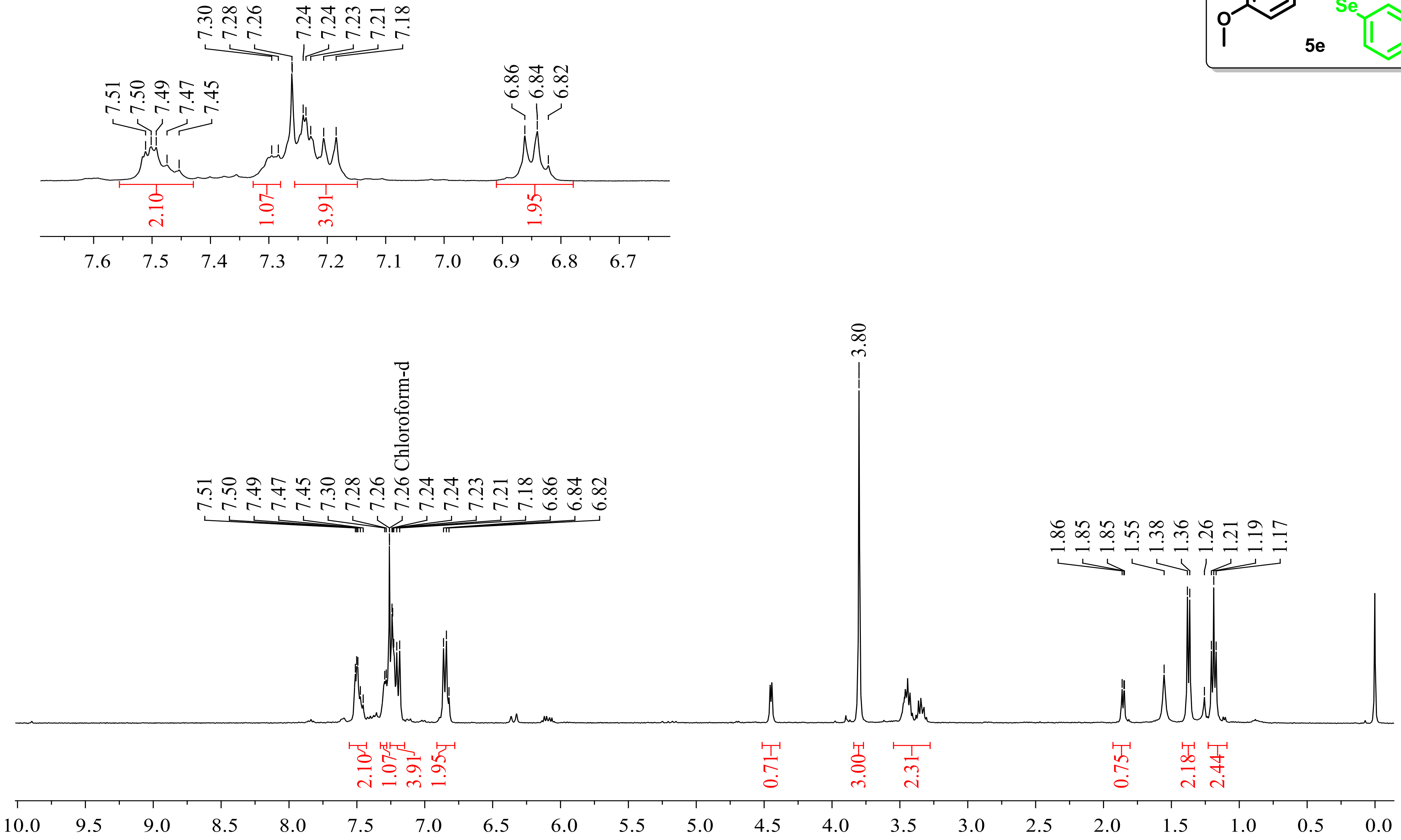

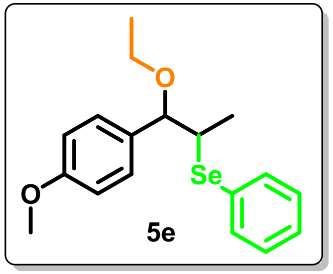

^{13}C NMR - (1-ethoxy-1-(4-methoxyphenyl)propan-2-yl)(phenyl)selane (5e).

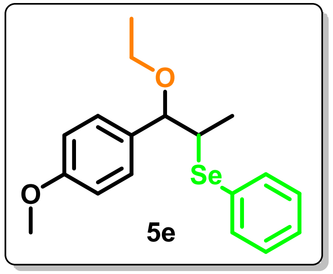

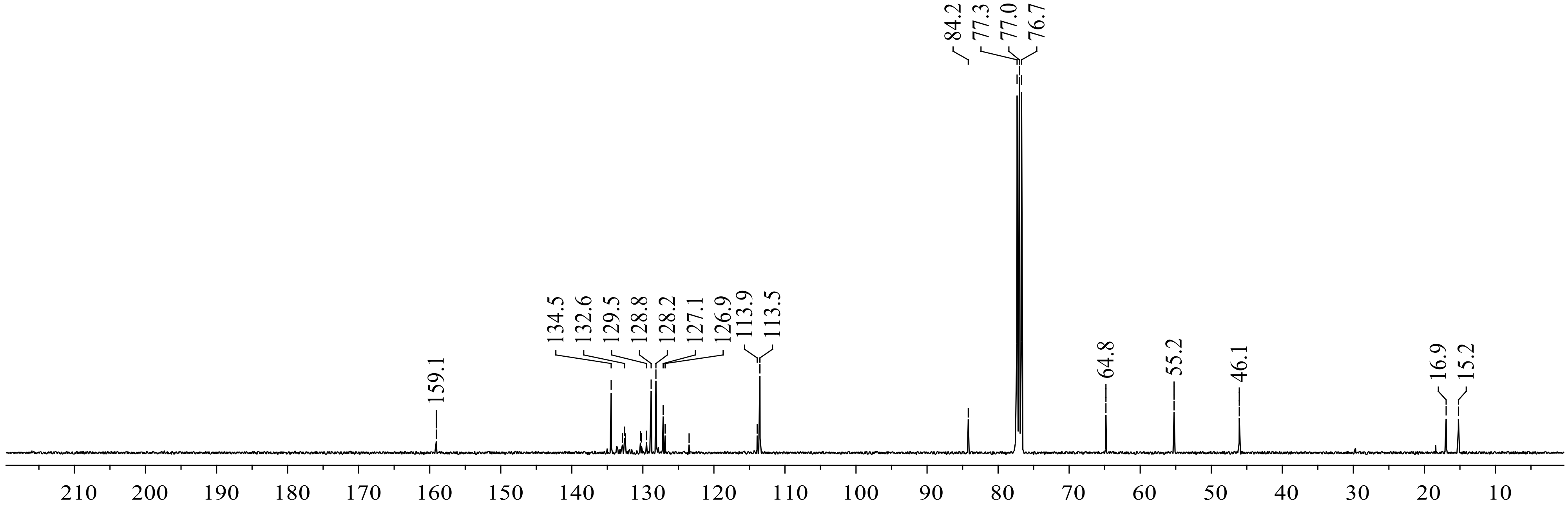

^{77}Se NMR - (1-ethoxy-1-(4-methoxyphenyl)propan-2-yl)(phenyl)selane (5e).

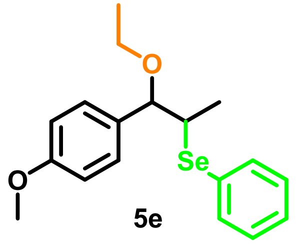

5e

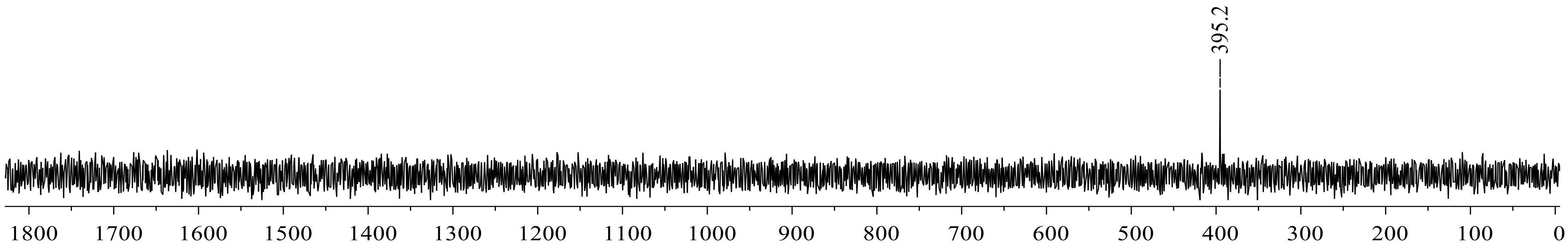

Supplement: RA-009-C9RA03642C-s001 [file RA-009-C9RA03642C-s001.pdf]
